# Effects of acupuncture-related therapies on endocrine and metabolic outcomes in obese women with polycystic ovary syndrome: a systematic review and network meta-analysis

**DOI:** 10.3389/fmed.2026.1758475

**Published:** 2026-05-28

**Authors:** Yi Liu, Pei Sheng, Jiakun Li, Erjia Weng, Shengqian Wang, Man Lu, Xiaofei An

**Affiliations:** 1 Affiliated Hospital of Nanjing University of Chinese Medicine, Nanjing, China; 2 The Second Clinical College of Guangzhou University of Chinese Medicine, Guangzhou, China; 3 Department of Endocrinology, Jiangsu Province Hospital of Chinese Medicine, Nanjing, China

**Keywords:** acupuncture, endocrine system, metabolism, network meta-analysis, obesity, polycystic ovary syndrome

## Abstract

**Background:**

Evidence on the effectiveness of acupuncture and combination therapies for metabolic and hormonal disturbances in obese women with PCOS remains limited. This NMA compared the relative efficacy of acupuncture-related interventions.

**Methods:**

RCTs evaluating acupuncture-based therapies for metabolic outcomes (BMI, HOMA-IR, TG) and endocrine parameters (T, LH/FSH ratio) in obese women with PCOS were included. A Bayesian NMA assessed the comparative effects of ten modalities. Continuous outcomes were reported as MD with 95% CrIs. SUCRA was used to rank the relative efficacy of each intervention, and certainty of evidence was evaluated using CINeMA. The study was registered in PROSPERO (CRD420251118032).

**Results:**

Forty-one RCTs involving 3,500 obese women with PCOS were included. Methodological quality was generally moderate, and certainty of evidence was low. Acupuncture and related therapies produced greater metabolic and hormonal improvements than Western medication. Mox_ACE showed the strongest metabolic benefits across BMI, HOMA-IR, and TG, while ACWM provided the most favorable hormonal regulation, improving both T and LH/FSH ratio. AC ranked within the top three for HOMA-IR, LH/FSH ratio, and TG, and AbdA ranked second to ACWM in reducing T.

**Conclusion:**

Acupuncture and related therapies may offer meaningful improvements in metabolic and endocrine profiles in obese women with PCOS. Low-certainty evidence suggests that acupuncture-related therapies may improve metabolic and endocrine profiles in obese women with PCOS. However, the clinical relevance of these findings remains uncertain pending high-quality trials.

**Systematic review registration:**

PROSPERO (CRD420251118032): https://www.crd.york.ac.uk/PROSPERO/view/CRD420

## 1 Introduction

Polycystic ovary syndrome (PCOS) is one of the most common endocrine and metabolic disorders among women of reproductive age, characterized by menstrual irregularities, hyperandrogenism, and polycystic ovarian morphology (1). It represents a leading cause of anovulatory infertility and early-onset type 2 diabetes in women. Obesity, a growing global health concern, has risen sharply from 6.4% to 14.9% among adult women between 1975 and 2014, imposing a significant socioeconomic and healthcare burden worldwide (2). Among the heterogeneous phenotypes of PCOS, the metabolic subtype, primarily associated with obesity, is recognized as the most prevalent form, accounting for approximately 37%–39% of all cases (3). Obesity contributes to PCOS by reducing sex hormone–binding globulin, increasing bioavailable androgens, and enhancing peripheral aromatization of estrogens, which disrupt the hypothalamic–pituitary–ovarian axis. It also exacerbates insulin resistance, creating a feedback loop that reinforces hyperandrogenism and metabolic dysfunction (4). It has been speculated that a bidirectional interaction may exist between obesity and PCOS. Women with PCOS have been shown to experience persistent overweight or obesity, with body mass index (BMI) trajectory deviations emerging as early as age 5 (5). Conversely, elevated BMI can trigger the onset of PCOS (6). Epidemiological data indicate that approximately 60% of women with PCOS are overweight or obese (7), and the prevalence of PCOS among obese women can reach up to 28.3%, compared with only 10%–13% in non-obese women of reproductive age (8). These findings suggest that obesity plays a pivotal role in the pathogenesis and clinical management of PCOS. Compared with non-obese individuals, obese women with PCOS tend to exhibit more pronounced insulin resistance, hyperandrogenism, and metabolic complications. They also face higher risks of cardiovascular disease and reproductive dysfunction, as well as a significantly greater incidence of emotional disorders (9). With the increasing prevalence of obesity-related PCOS, together with socioeconomic development and heightened public awareness of health and body composition, the prevention and management of obese PCOS have gradually become key focuses in both clinical practice and scientific research.

PCOS is primarily caused by dysregulation of ovarian androgen secretion, leading to functional ovarian hyperandrogenism. Insulin resistance, endocrine and metabolic abnormalities, and environmental and genetic factors contribute to the development of PCOS (10). PCOS exhibits significant clinical heterogeneity, resulting in highly varied and goal-oriented treatment approaches. Individualized symptomatic interventions must be tailored to patient complaints, treatment needs, and metabolic alterations. Overall, the therapeutic objectives of PCOS extend beyond the management of current symptoms and encompass the reduction of long-term complication risks. Short-term goals focus on alleviating existing clinical manifestations, including regulation of menstrual cycles, improvement of ovulatory function and fertility, and treatment of hyperandrogenic features such as acne and hirsutism. In parallel, long-term management aims to reduce metabolic and cardiometabolic risks, including insulin resistance, type 2 diabetes, and cardiovascular disease, while also addressing sleep disturbances, psychological features, and long-term weight management (1). Importantly, PCOS is increasingly recognized as a lifelong condition, making sustained lifestyle management throughout the lifespan a core focus in PCOS treatment (11). Lifestyle management should be recommended for all women with PCOS, incorporating behavioral strategies, dietary intervention, and exercise intervention. In non-obese PCOS patients, lifestyle management focuses on a healthy lifestyle and the prevention of excess weight gain (12). However, poor long-term adherence to lifestyle interventions often limits their clinical efficacy (5). Consequently, pharmacological treatment is frequently employed as a targeted therapeutic approach to address specific reproductive, metabolic, or hyperandrogenic manifestations. Metformin, the most widely used insulin sensitizer, improves insulin sensitivity and reduces androgen levels but frequently causes gastrointestinal discomfort and may induce vitamin B₁₂ deficiency with prolonged use (12). Thiazolidinediones and inositols (13) also have limitations, as thiazolidinediones may lead to fluid retention and inositols provide only modest improvements in ovulation, hirsutism, and weight control (1). Newer agents such as GLP-1 receptor agonists, SGLT-2 inhibitors, and *α*-glucosidase inhibitors show metabolic benefits and promote weight loss, although their use is restricted by gastrointestinal symptoms, urinary tract infections, and hypotension (14, 15). Orlistat can further assist weight reduction but often causes steatorrhea, diarrhea, and elevated liver enzymes (16). Reproductive medications, including letrozole, clomiphene citrate (CC), and combined oral contraceptives (COCs), are effective but associated with menopausal-like symptoms, contraindications, and increased risks such as venous thromboembolism (1, 5, 17), making long-term use unsuitable. Overall, although Western pharmacological therapies improve metabolic and reproductive outcomes, their adverse effects, limited tolerability, and uncertain long-term safety contribute to poor adherence in many obese women with PCOS.

In traditional Chinese medicine (TCM), there is no specific disease entity corresponding to PCOS. Based on its clinical manifestations, PCOS is classified under the TCM categories of “amenorrhea,” “metrorrhagia,” and “infertility,” (18). The therapeutic principle focuses on resolving phlegm, eliminating dampness, and regulating menstruation, with modified formulas such as *Cangfu Daotan Wan* commonly employed in clinical practice. However, traditional Chinese herbal medicine requires long-term administration, and the preparation and consumption processes are often cumbersome, resulting in limited patient adherence. In TCM, it is suggested that acupuncture unblocks energy routes (meridians) to regulate vital energy (qi) and blood, and harmonizes the body and mind (yin and yang). Evidence suggests that acupuncture may exert beneficial effects on PCOS by modulating the hypothalamic–pituitary–ovarian axis, improving insulin resistance, promoting ovulation, and restoring normal menstrual cycles (19, 20). Although acupuncture may cause minor adverse reactions such as bleeding, bruising, and transient pain, serious complications are rare. Compared with oral medications, acupuncture is generally considered safe and well tolerated and is increasingly used as a complementary approach in the comprehensive management of obese PCOS.

An increasing number of patients are turning to acupuncture as a therapeutic option for various conditions, including PCOS. An epidemiological study in the United States reported that about 22% of patients had used acupuncture for infertility treatment (21). With the growing number of clinical trials and meta-analyses, evidence supporting acupuncture’s efficacy in managing PCOS continues to expand. Several meta-analyses have shown that acupuncture can improve fertility outcomes by increasing pregnancy and ovulation rates, along with other reproductive indicators in PCOS patients (22, 23). Other studies have also found that acupuncture can improve metabolic abnormalities such as insulin resistance and hyperandrogenism (24–26). However, some reports have questioned these findings (27, 28). Although many meta-analysis have confirmed the therapeutic benefits of acupuncture in PCOS, most compared only one or two acupuncture modalities or examined acupuncture versus conventional treatments. In clinical practice, however, acupuncture and related therapies are often used in combination. Previous studies have not fully reflected the diversity of treatment regimens used in real-world settings (29, 30). In addition, many comparative studies did not clearly distinguish acupuncture from other TCM interventions, such as herbal decoctions or patent medicines (31, 32). Few studies have compared the efficacy of different acupuncture-related modalities for PCOS, and meta-analyses focusing on metabolic and hormonal outcomes in obese PCOS patients are still limited. To address these gaps, we conducted a systematic review and Network Meta-Analysis (NMA) to compare the relative efficacy of multiple acupuncture-related interventions, including Manual Acupuncture (AC), Abdominal Acupuncture (AbdA), EA(Electroacupuncture), Acupoint Catgut Embedding (ACE), and several combination therapies such as Moxibustion plus Acupoint Catgut Embedding (Mox_ACE), Acupuncture plus Acupoint Catgut Embedding (AC_ACE), and Acupoint Catgut Embedding plus Auricular Acupressure (ACE_AAp). The study evaluated their effects on endocrine indicators (T, LH/FSH ratio) and metabolic parameters (BMI, HOMA-IR, TG), and also explored potential underlying mechanisms. The findings provide updated evidence on the therapeutic value of acupuncture and its combinations in obese women with PCOS and offer meaningful insights for future research and clinical practice.

## 2 Methods

This systematic review and NMA was pre-registered in the PROSPERO database of systematic reviews (CRD420251118032; https://www.crd.york.ac.uk/PROSPERO/view/CRD420251118032), and the findings of this meta-analysis adhere to the Preferred Reporting Items for Systematic Reviews and Meta-Analysis (PRISMA) guidelines (Supporting Information S1 PRISMA 2020 Checklist).

### 2.1 Search strategy

Electronic searches were conducted using four literature databases: PubMed, Web of Science, Cochrane Library, Embase, Chinese National Knowledge Infrastructure (CNKI), Wanfang Database, Chinese Science and Technique Journals Database (VIP) and SinoMed. The search language was English or Chinese, and the search period for each database was extended from its establishment to August 8, 2025. The method employed to search for entailed amalgamating subject terms using free text terms. The search terms used were: “(Polycystic Ovary Syndrome) AND acupuncture AND (Randomized Controlled Trial).” The specific search contents are detailed in Supporting Information S2; The screening process was cross-verified by two researchers, and any inconsistencies were resolved through discussion to reach a consensus.

### 2.2 Eligibility criteria

A (Patients): Inclusion criteria were defined as follows: (1) Diagnosis of PCOS according to one of the following recognized clinical criteria: Rotterdam criteria (ESHRE/ASRM, 2003) (33); The “Chinese guideline for diagnosis and management of polycystic ovary syndrome” published by the Chinese Journal of Obstetrics and Gynecology in 2008 (18), as well as the “Diagnostic criteria for polycystic ovary syndrome: health industry standard of the People’s Republic of China (34)”; (2) Diagnosis of obesity based on the World Health Organization (WHO) Asia-Pacific guidelines (2000): Body mass index (BMI) ≥ 25 kg/m^2^. Among Asian populations, multiple consensuses and epidemiological studies have adopted BMI ≥ 23 kg/m^2^ as the cut-off for overweight or “high-risk obesity,” including the Korean Obesity Society Guidelines and the WHO Asia-Pacific Perspective (35, 36). Therefore, in addition to studies that defined obesity as BMI ≥ 25 kg/m^2^, this meta-analysis also accepted studies that used BMI ≥ 23 kg/m^2^ as the inclusion criterion; (3) Age ≥ 18 years; (4) Signed informed consent form.

Exclusion criteria were as follows: (1) Hyperandrogenism caused by hyperprolactinemia, thyroid disorders, congenital adrenal hyperplasia, or Cushing’s syndrome; (2) Genital tract malformations, gonadal dysgenesis, or fallopian tube obstruction; (3) Pathological endometrial conditions, including uterine malformations or uterine fibroids; (4) Severe cardiac, hepatic, renal, pulmonary, hematological, or psychiatric disorders; (5) Known allergic conditions; (6) Pregnant women; There was no pregnancy at baseline and the outcome metrics needed to be measured in a non-pregnant period. If pregnancy occurred during follow-up and the outcome was measured only after pregnancy or pre-pregnancy data could not be differentiated, the outcome was not included in the analysis.

B (Interventions): Includes acupuncture alone, requiring insertion of acupuncture needles into the skin, such as: AC, EA, AbdA, ACE; moxibustion alone, requiring the use of mugwort or its derivatives for moxibustion, such as: Moxibustion; Combined two acupuncture methods or acupuncture combined with moxibustion interventions, such as: Mox_ACE, AC_ACE, ACE_AAp, Electroacupuncture plus Auricular Acupressure (EA_AAp), Electroacupuncture plus Acupoint Catgut Embedding(EA_ACE); As well as the Acupuncture Combined with Medication group (ACWM), which combines the aforementioned acupuncture methods with Western medications (e.g., Acupoint Catgut Embedding plus metformin, or Moxibustion plus metformin). Acupoint selection, manipulation techniques, and needle retention time were all left unrestricted.

C (Controls): The control group was the western medicine (WM) group. According to the 2023 International Evidence-Based Guideline for the Assessment and Management of PCOS, conventional western medicine treatments comprise: (1) agents targeting metabolic abnormalities, such as metformin and acarbose; (2) COCs, including formulations containing cyproterone acetate with ethinylestradiol, such as Diane-35, or drospirenone with ethinylestradiol, such as Yasmin, which are recommended for menstrual regulation and reduction of hyperandrogenic symptoms; (3) ovulation-inducing agents, most notably letrozole and CC; and (4) the gastrointestinal lipase inhibitor orlistat.

D (Outcomes): Primary outcome measures were BMI (kg/m^2^), homeostatic model assessment of insulin resistance (HOMA-IR), and total testosterone (T, nmol/L). Fasting glucose was expressed in mmol/L and fasting insulin in mU/L (μU/mL); HOMA-IR was calculated as [glucose × insulin]/22.5.

Secondary outcome measures included the luteinizing hormone to follicle-stimulating hormone ratio (LH/FSH ratio), in which LH and FSH were expressed in IU/L and the ratio is unitless, and triglycerides (TG, mmol/L). BMI reflects body weight status; HOMA-IR indicates the degree of insulin resistance; T reflects hyperandrogenism. The LH/FSH ratio is used to assess ovulatory dysfunction and endocrine imbalance, while TG serves as a marker of metabolic disturbance and dyslipidemia.

E (Studies): RCTs (Randomized Controlled Trials) with double-blind, single-blind, or open-label designs were included.

### 2.3 Data extraction

The relevant information was extracted by two authors using a predefined set of characteristics for the studies. The following details were extracted from each included study: first author and publication year, patient characteristics, sample size, disease duration, specific intervention measures, acupuncture intervention details (including: specific acupoints, techniques, needle insertion depth, treatment frequency, treatment duration, course of treatment, etc.), period of treatment, and corresponding outcome measures. Any disagreements were resolved through discussion with the third author. We assessed the risk of bias in the included studies using the Cochrane Collaboration’s Risk of Bias (ROB) tool (37). We assessed the confidence in the NMA results using the Confidence in Network Meta-Analysis (CINeMA) framework, which evaluates six domains: within-study bias, across-studies bias, indirectness, imprecision, heterogeneity, and incoherence (38, 39). For continuous outcomes, imprecision was judged using the concept of the minimally clinically important difference (MCID). In the absence of established MCIDs for the included outcomes (BMI, HOMA-IR, LH/FSH ratio and T), a distribution-based approach was applied. Baseline standard deviations (SDs) were extracted from all eligible RCTs. Study-level *SDs* were subsequently pooled using a weighted formula that accounted for sample size and within-study variance to obtain a combined standard deviation *SD_pooled_*, where *n_i_* denotes the number of participants in each study group and *SD_i_* denotes the standard deviation associated with the mean change or baseline measurement for that group (40).

$$SD_{\text{pooled}}=\sqrt{\frac{\sum (n_{i}-1)SD_{i}^{2}}{\sum (n_{i}-1)}}$$

The primary MCID threshold was defined as 0.5 × *SD_pooled_*, in accordance with the conventional distribution-based criterion (41). In addition, the clinical relevance of treatment effects was evaluated by comparing the magnitude of mean differences (MDs) with the predefined MCID thresholds.

### 2.4 Statistical analysis

We performed Bayesian NMA using the “GeMTC” package in R software (version 4.4.3) and JAGS (version 4.3.1). We used a Bayesian Markov chain Monte Carlo (MCMC) approach to estimate the posterior distributions of relative treatment effects among acupuncture-related therapies. The estimation employed three independent chains, each subjected to an initial burn-in of 20,000 iterations, after which 50,000 iterations were retained for inference. Convergence was evaluated using the Brooks–Gelman–Rubin potential scale reduction factor (PSRF) with shrink-factor plots and by visual inspection of trace/density plots; all parameters had PSRF close to 1 (upper 97.5% ≤ 1.05), indicating adequate convergence. MDs and their corresponding 95% credible intervals (95% CrIs) were reported as effect sizes.

Inconsistency, defined as a discrepancy between direct and indirect evidence, was assessed both globally and locally. To quantify global inconsistency, we contrasted the Deviance Information Criterion (DIC) for the consistency and inconsistency model specifications. We used ΔDIC=DIC_consistency−DIC_inconsistency as the primary metric. Values ≥5 were interpreted as materially favoring the inconsistency specification and therefore suggesting potential global inconsistency. Values <5 were taken as providing insufficient evidence to favor the inconsistency model. For completeness, we also examined total residual deviance to ensure that any improvement in fit was not solely attributable to increased model complexity (42). Local inconsistency was examined through the node-splitting method, contrasting direct with indirect evidence (43).

Heterogeneity was summarised by the posterior of the common between-study variance (*τ*^2^) and interpreted on the natural scale against a prespecified MCID. Given the scale-dependence of I^2^for continuous outcomes, inference focused on τ/τ^2^, with network-level I^2^ reported only as a descriptive supplement.

Transitivity was assessed via meta-regression and sensitivity analysis. Meta-regression tested whether study-level covariates (age, sample size, publication year, acupuncture frequency and Period of treatment) modified relative treatment effects. For sensitivity analysis, we treated each ACWM group as a separate node outside the control condition and re-ran the NMA. Aggregated versus disaggregated models were compared on relative effects (MD, 95% CrIs).

Interventions were ranked by the Surface under the Cumulative Ranking Curve (SUCRA), with larger values indicating greater relative efficacy. Publication bias was assessed using funnel plots and Egger’s regression test.

## 3 Results

### 3.1 Characteristics of the included studies

**Figure 1:**
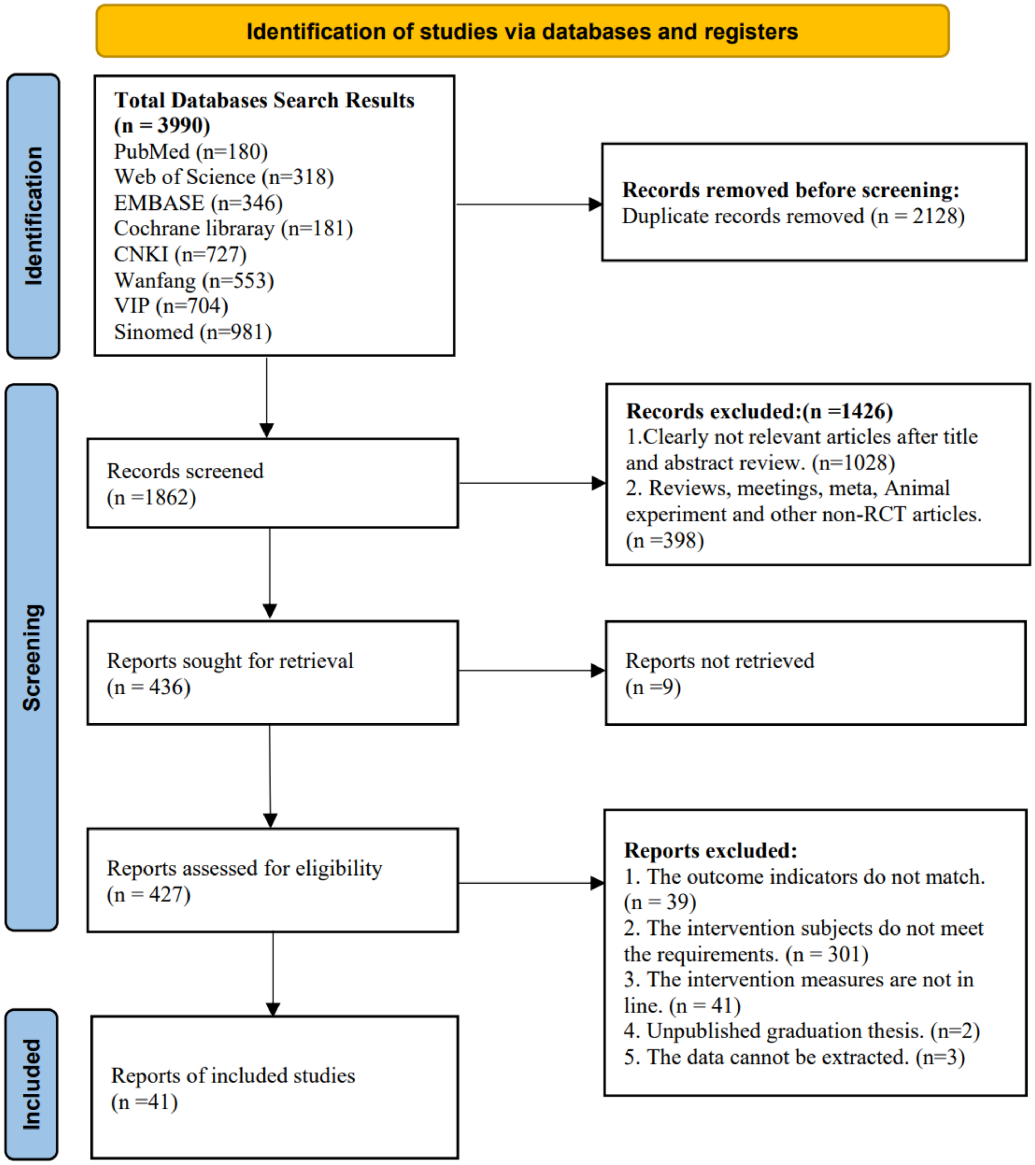
PRISMA flow diagram of study selection.

Among the 3,990 studies screened, 180 were sourced from PubMed, 318 from Web of Science, 346 from EMBASE, 181 from the Cochrane Library, 727 from CNKI, 553 from Wanfang, 704 from VIP, and 981 from Sinomed. After removing duplicates using EndNote and manual review, titles and abstracts were screened. Full-text articles were then reviewed to exclude ineligible studies, resulting in the inclusion of 41 studies. The literature search flowchart is shown in Figure 1. A total of 41 studies were included (44–84), with 1,855 participants in the experimental group and 1,645 in the control group. The experimental group size ranged from 14 to 107 participants. The mean age ranged from 23.21 ± 4.58 to 33.5 ± 3.9 years, and disease duration varied from 36 days to over 4 years. Tri-arm trials were permitted. Interventions included: WM group using conventional Western medicine alone (36 studies), ACWM group combining Western medicine with acupuncture (21 studies, encompassing any combination therapy involving Western medicine and acupuncture-related modalities), AC alone (5 studies), combined AC and ACE therapy (1 study), EA alone (5 studies), combined EA and AAp therapy (1 study), combined EA and ACE therapy (1 study), ACE alone (8 studies), combined ACE and Moxibustion therapy (3 studies), combined ACE and AAp therapy (1 study), and AbdA alone (4 studies). Detailed acupuncture points and intervention techniques are provided in Supporting Information S3. Treatment durations ranged from 1 to 6 months, with an average of 3.46 months. Regarding outcome measures, BMI was the most frequently reported indicator (36 studies), followed by T (26 studies), HOMA-IR (21 studies), LH/FSH ratio (20 studies), and TG (12 studies). Table 1 presents the characteristics of the included studies.

**Table 1:** Characteristics of the included studies.

Study (authors, year)	Experimental group					Control group				Period of treatment	Outcomes
	Sample	Age	Disease duration	Intervention A	Intervention B	Sample	Age	Disease duration	Intervention C		
Zheng et al. (2013) (44)	43	26.5 ± 3.0	42 ± 33.6	AbdA		43	24.9 ± 4.9	43.2 ± 26.4	Metformin	6 m	①②③④⑤
Cao et al. (2023) (45)	61	28.4 ± 4.42	NA	EA + Placebo		65	28.76 ± 4.08	NA	Sham EA + Metformin	4 m	①②③
Muharam et al. (2022) (46)	22	27.9 ± 4.09	NA	EA + Metformin		22	28.14 ± 3.21	NA	Sham EA + Metformin	4w	①②
Wu et al. (2024) (47)	42	26.95 ± 3.8	101.28 ± 69.36	AC + CC		39	27.28 ± 2.74	101.64 ± 69.36	CC	4 m	②③④⑤
Yue et al. (2020) (48)	40	26.68 ± 4.56	30.5(15.567.5)	AC		40	27.45 ± 4. 31	34.2(23.4,37.5)	COCs	3 m	①③④
Yue et al. (2021) (49)	30	26.6 ± 4.49	43.08 ± 26.28	AC		30	27.8 ± 4.23	45.00 ± 25.20	COCs	3 m	①③④
Zeli (2016) (50)	50	26.3 ± 4.4	38.4 ± 7.2	AC+ Metformin		50	27.2 ± 4.1	37.2 ± 6.0	Metformin	6 m	①③④
Xiaoli and Jinfang (2023) (51)	107	31.13 ± 8.90	178.56 ± 53.16	AC		106	30.2 ± 9.45	187.08 ± 51.0	Metformin	6 m	①②③④⑤
Jiali and Zhongcheng (2009) (52)	30	25.8 ± 2.6	5 ~ 96	AC_ACE		30	25.8 ± 2.6	5 ~ 96	Metformin	3 m	①
Danjuan et al. (2020) (53)	60/62	A:23.22 ± 4.47	A:23.36 ± 9.08	ACE	ACE+COCs+Metformin	62	23.35 ± 4.26	24.58 ± 10.25	COCs+Metformin	3 m	①③④
		B:23.21 ± 4.58	B:22.73 ± 9.77								
Huili et al. (2023) (54)	30	27.87 ± 5.23	56.4 ± 86.4	ACE+Orlistat		30	27.62 ± 5.35	55.2 ± 100.8	Orlistat	12w	①③
Ting et al. (2025) (55)	30	27.96 ± 3.15	38.64 ± 5.04	Mox_ACE		30	27.96 ± 3.15	38.64 ± 5.04	Metformin	3 m	①②⑤
Limei et al. (2025) (56)	40	30.38 ± 5.01	31.20 ± 13.32	ACE+ Metformin		40	29.80 ± 4.68	31.32 ± 12.96	Metformin	3 m	①②③
Gui-zhi et al. (2020) (57)	40	26 ± 2	58.8 ± 2.4	ACE+ Metformin		41	25 ± 1	61.2 ± 3.6	Metformin	3 m	①②⑤
Dan-juan et al. (2020) (58)	62/63	A:25 ± 7	A:27.14 ± 13.02	ACE	ACE+Metformin	60	25 ± 6	26.85 ± 12.92	Metformin	3 m	②⑤
		B:24 ± 6	B:28.07 ± 12.37								
Jianfeng et al. (2016) (59)	29	23.36 ± 12.36	49.44 ± 19.44	ACE		28	24.21 ± 13.13	58.08 ± 14.04	Metformin	3 m	①②
Li (2018) (60)	30	26.51 ± 5.76	56.16 ± 45.72	ACE		30	26.41 ± 5.97	56.16 ± 43.44	Metformin	3 m	①
Jing et al. (2014) (61)	20	25.64 ± 3.95	59.52 ± 4.72	ACE+COCs		12	24.75 ± 3.87	53.64 ± 45.12	COCs	3 m	①③
Yu′e et al. (2024) (62)	31	27.15 ± 5.21	52.32 ± 45.28	AC		31	26.34 ± 4.26	52.28 ± 43.13	Metformin	3 m	①②③⑤
Xiaoli and Wenhua (2022) (63)	30/30	A:33.5 ± 3.9	NA	Mox_ACE	Mox + ACE +COCs+ Metformin	30	34.5 ± 2.5	NA	COCs+ Metformin	12w	①④③⑤
		B:32.7 ± 4.2									
Dan-shan and Jian-ye (2019) (64)	30	28 ± 2	50.4 ± 31.2	Mox_ACE		30	29 ± 2	54.0 ± 22.8	Metformin + CC	3 m	①②③④
Hongmei (2021) (65)	23	29.92 ± 3.17	36.36 ± 6.72	Mox+Metformin		22	30.05 ± 3.25	39.36 ± 8.52	Metformin	3 m	①②③
Wenzhen (2021) (66)	60	24.35 ± 2.26	24.36 ± 9.08	ACE		60	23.68 ± 2.37	23.68 ± 8.68	COCs+ Metformin	3 m	①③
Ning et al. (2016) (67)	14/14	18 ~ 36	NA	AbdA	AbdA+Metformin	14	18 ~ 36	NA	Metformin	6 m	①③④
Ling et al. (2020) (68)	34	26.06 ± 8.11	NA	AbdA		34	25.55 ± 9.21	NA	Metformin	6 m	③④
Maohua et al. (2010) (69)	43	26.5 ± 3.0	42.0 ± 33.6	AbdA		43	24.9 ± 4.9	43.2 ± 26.4	Metformin	6 m	①③④⑤
Jia (2020) (70)	30	27.35 ± 2.47	38.76 ± 4.80	ACE		29	27.19 ± 2.81	37.08 ± 2.64	COCs+ Metformin	3 m	①③
Zhenyuan (2017) (71)	40	23.7 ± 2.2	52.8 ± 14.4	ACE+Metformin		40	23.1 ± 1.9	49.2 ± 16.8	Metformin	3 m	①③
Cheng-yi and Chun-ye (2024) (72)	30	27.23 ± 4.96	48(36,60)	AC+Metformin		30	27.47 ± 4.24	48(45,60)	Metformin	3 m	①
Wen-hui et al. (2020) (73)	30	27.23 ± 2.58	22.73 ± 19.76	EA + Metformin		30	27.03 ± 3.15	18.93 ± 14.52	Metformin	3 m	①③⑤
Xianbing et al. (2016) (74)	25/30	A:29.5 ± 2.3	NA	EA	ACE	25	29.5 ± 2. 3	NA	Metformin	3 m	①②③④
		B:29.5 ± 2. 3									
Yaqin (2017) (75)	64	28.3 ± 4.2	63.6 ± 30.0	AC+Metformin		64	27.9 ± 4.3	64.8 ± 32.4	Metformin	3 m	①②③④
Dan et al. (2020) (76)	56	28.64 ± 3.73	57.84 ± 27.24	AbdA+Metformin		56	29.43 ± 2.97	54.36 ± 33.00	Metformin	3 m	①④
Juan et al. (2019) (77)	80	18 ~ 30	NA	AC + Acarbose		80	18 ~ 30	NA	AC+Placebo	6 m	①②③⑤
Liangying et al. (2015) (78)	30	23.4 ± 12.4	49.2 ± 19.2	ACE+Metformin		30	24.2 ± 13.1	58.8 ± 14.4	Metformin	3 m	①④
Tong et al. (2013) (79)	27	29.56 ± 2.83	48.00 ± 20.7	ACE		25	30.16 ± 3.579	55.20 ± 21.96	EA	3 m	①②④④
Guochao et al. (2021) (80)	53	32.75 ± 6.18	42.60 ± 13.44	AC+Letrozole		53	33.45 ± 6.29	38.52 ± 12.48	Letrozole	3 m	①④
Xing et al. (2023) (81)	67	29.85 ± 5.64	NA	ACE_AAp		67	30.31 ± 5.94	NA	ACE	3 m	②
Yuchang et al. (2020) (82)	30	29.7 ± 2.6	25.2 ± 6.0	AAp+Metformin		30	29.5 ± 2.4	24.0 ± 3.6	Metformin	3 m	①②⑤
Linan et al. (2015) (83)	36	36.44 ± 3.11	43.92 ± 9.36	EA_AAp		36	36.89 ± 4.19	NA	EA	1 m	②
Jiaxin et al. (2019) (84)	27	27.19 ± 4.85	NA	EA_ACE		28	27.29 ± 4.99	NA	EA	3 m	①②③④

COCs, combined oral contraceptives; CC, clomiphene citrate; AC, manual acupuncture; EA, electroacupuncture; AbdA, abdominal acupuncture; ACE, acupoint catgut embedding; Mox, moxibustion; Mox_ACE, moxibustion plus acupoint catgut embedding; AC_ACE, acupuncture plus acupoint catgut embedding; ACE_AAp, acupoint catgut embedding plus auricular acupressure; EA_AAp, electroacupuncture plus auricular acupressure; EA_ACE, electroacupuncture plus acupoint catgut embedding; ①, BMI; ②, HOMA-IR; ③, T; ④, LH/FSH ratio; ⑤, TG.

### 3.2 Risk of bias assessment

According to the Cochrane Collaboration’s Risk of Bias (RoB) tool, the risk of bias results for the 41 included studies were as follows: For selection bias, nearly all studies reported random sequence generation, using methods such as random number tables or computer-generated random sequences. Two studies were non-randomized and were therefore rated as high risk. Because these studies did not employ random allocation, their selection bias was also judged to be high risk. For performance bias, due to the different nature of interventions between the acupuncture and conventional medicine groups, it was nearly impossible to achieve double-blinding for both participants and practitioners. Only three studies were rated as low risk, while the remaining studies were rated as unclear risk.

With respect to detection bias, two studies reported blinding in outcome assessment and were rated as low risk. One study did not report the reasons for attrition and was rated as unclear risk for attrition bias. None of the studies identified other potential sources of bias that could have affected the validity of the results. Detailed results are provided in Supporting Information S4.

#### 3.2.1 Network meta-analysis results

**Figure 2:**
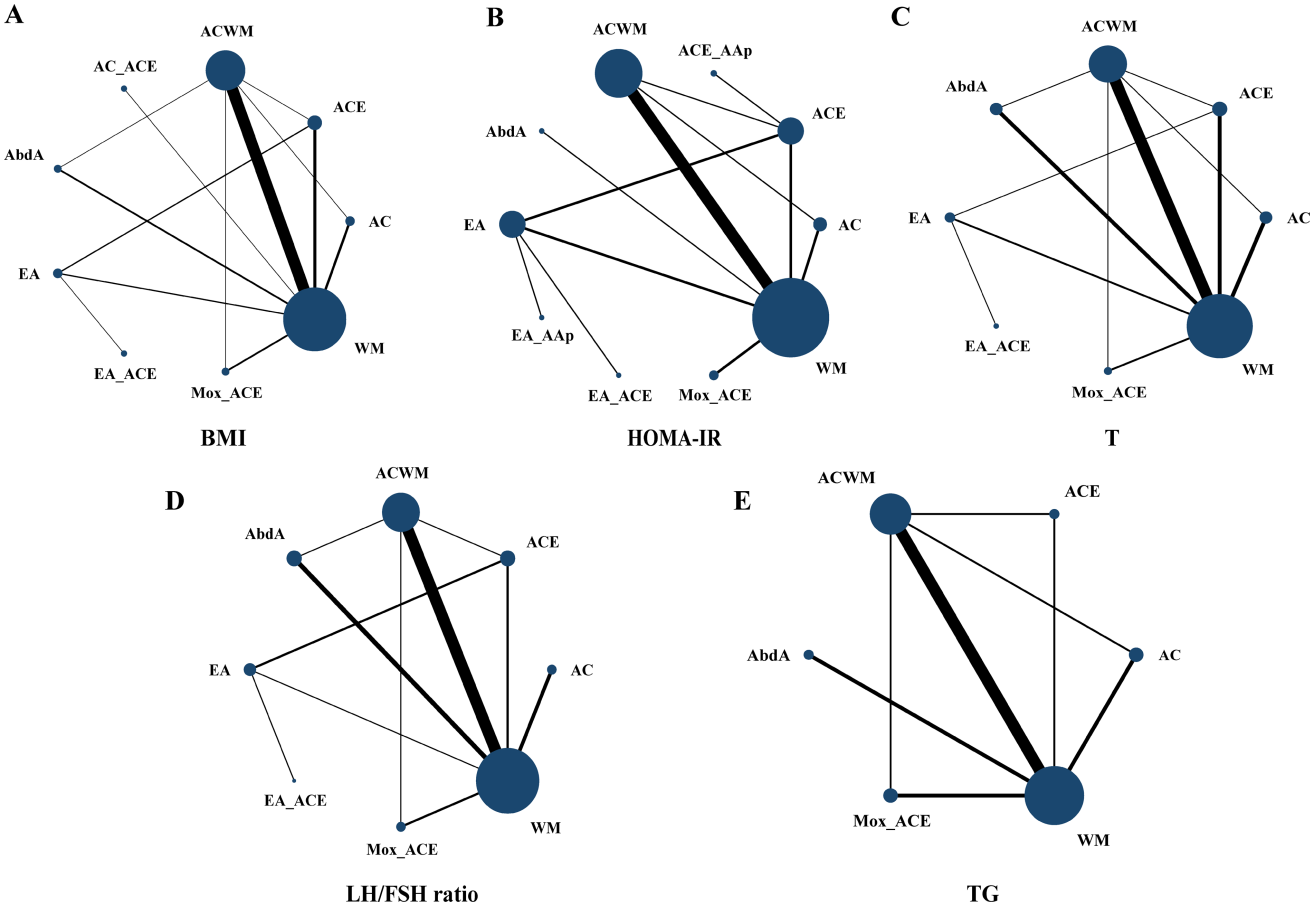
Network plots for BMI, HOMA-IR, T, LH/FSH ratio, and TG.

To visualize the direct relationships among interventions and the corresponding levels of evidence, network plots were generated for each of the five outcomes (Figure 2). In these plots, nodes represent individual interventions, with their sizes proportional to the total sample size of the contributing trials. Edges between nodes indicate head-to-head comparisons, where the thickness of each line reflects both the number of studies and the relative strength of the evidence. Most interventions centered on the WM group, which had the largest number of participants. A significant number of direct comparative studies were conducted between the ACWM group and the WM group.

#### 3.2.2 Consistency, heterogeneity, meta-regression and sensitivity analysis

The overall network model demonstrated satisfactory internal validity and coherence across all outcomes. Global inconsistency among the five outcomes was not statistically significant, as indicated by a deviance information criterion (DIC) difference of less than 5 between the consistency and inconsistency models (ΔDIC < 5). Accordingly, the consistency model was adopted for the NMA. The node-splitting analysis revealed no significant local inconsistency across most comparisons (*p* > 0.05). However, for the T outcome, local inconsistency was detected between ACWM and AbdA, whereas the remaining comparisons showed good agreement between direct and indirect estimates. Funnel plots appeared generally symmetrical, suggesting no apparent publication bias. The estimated heterogeneity was small and within an acceptable range. Meta-regression analyses identified no significant trial-level covariates influencing the relative treatment effects. Sensitivity analyses confirmed the robustness of the findings. Decomposition of ACWM into independent nodes resulted in slightly higher effect estimates for some interventions, but SUCRA rankings and relative effects remained largely unchanged. In addition, excluding the single study with a BMI ≥ 23 kg/m^2^ cut-off (46) did not materially affect effect estimates, statistical significance, or treatment rankings. Overall, results from the pooled and node-split models were comparable, and the network demonstrated good consistency and stability across outcomes (Supplementary material S5–S9).

#### 3.2.3 Results for BMI

**Figure 3:**
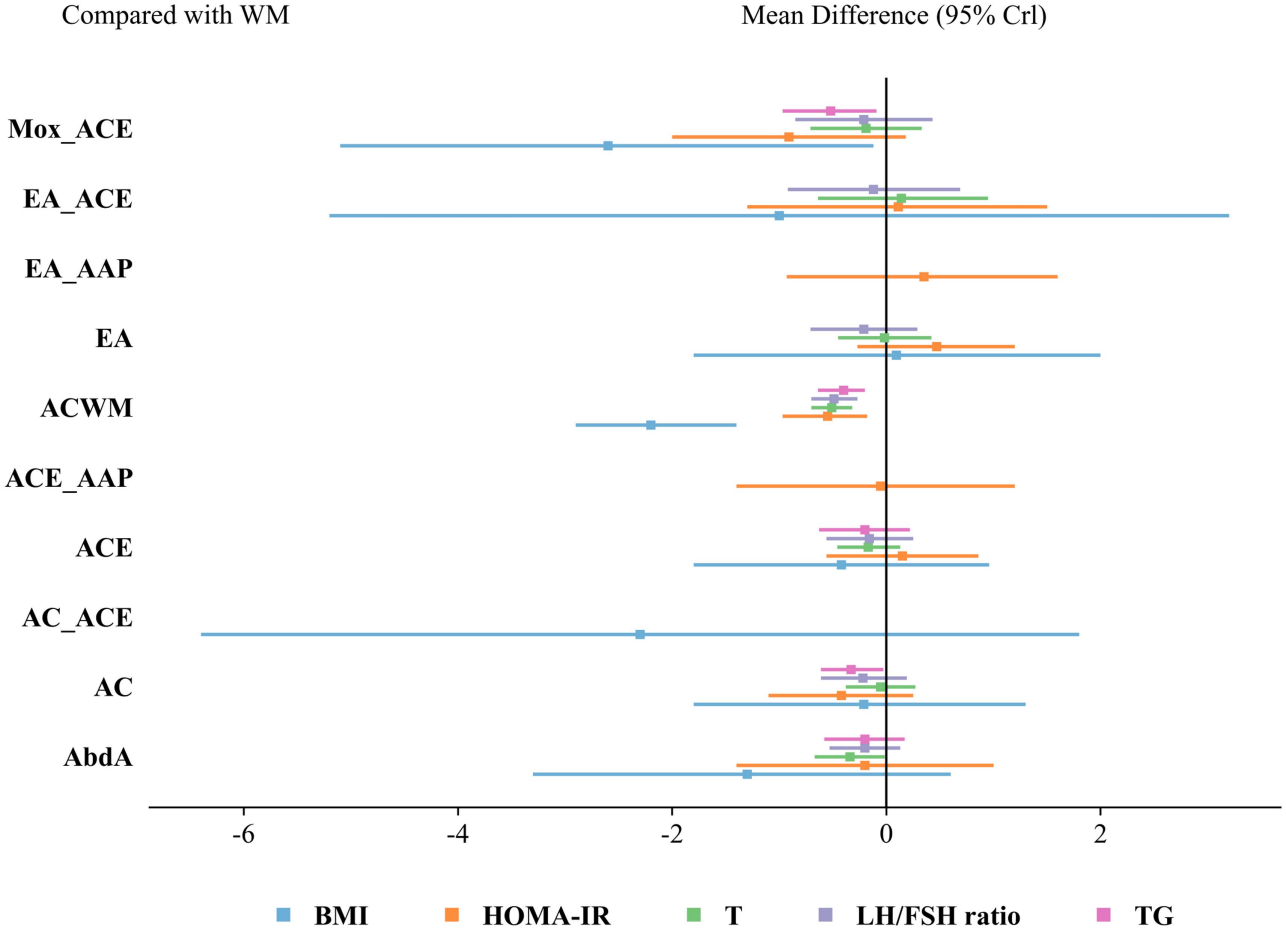
Forest plot of MDs with 95% CrIs for all interventions compared with WM. Points indicate mean differences and horizontal lines show the 95% CrIs. Results are presented for BMI, HOMA-IR, T, LH/FSH ratio, and TG.

**Figure 4:**
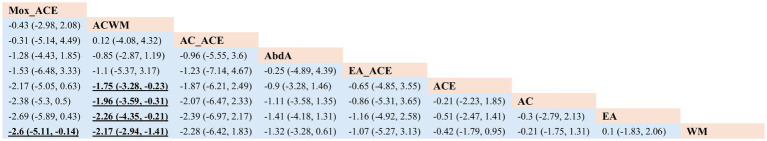
League table of relative treatment effects for BMI. Comparisons between treatments should be read from left to right, and the estimate is shown in the cell in common between the column-defining and row-defining treatments. Effect sizes are presented as MD with 95% CrIs. Mox_ACE, moxibustion plus acupoint catgut embedding; ACWM, acupuncture combined with medication; AC_ACE, acupuncture plus acupoint catgut embedding; AbdA, abdominal acupuncture; EA_ACE, electroacupuncture plus acupoint catgut embedding; ACE, acupoint catgut embedding; AC, manual acupuncture; EA, electroacupuncture; WM, western medicine.

**Figure 5:**
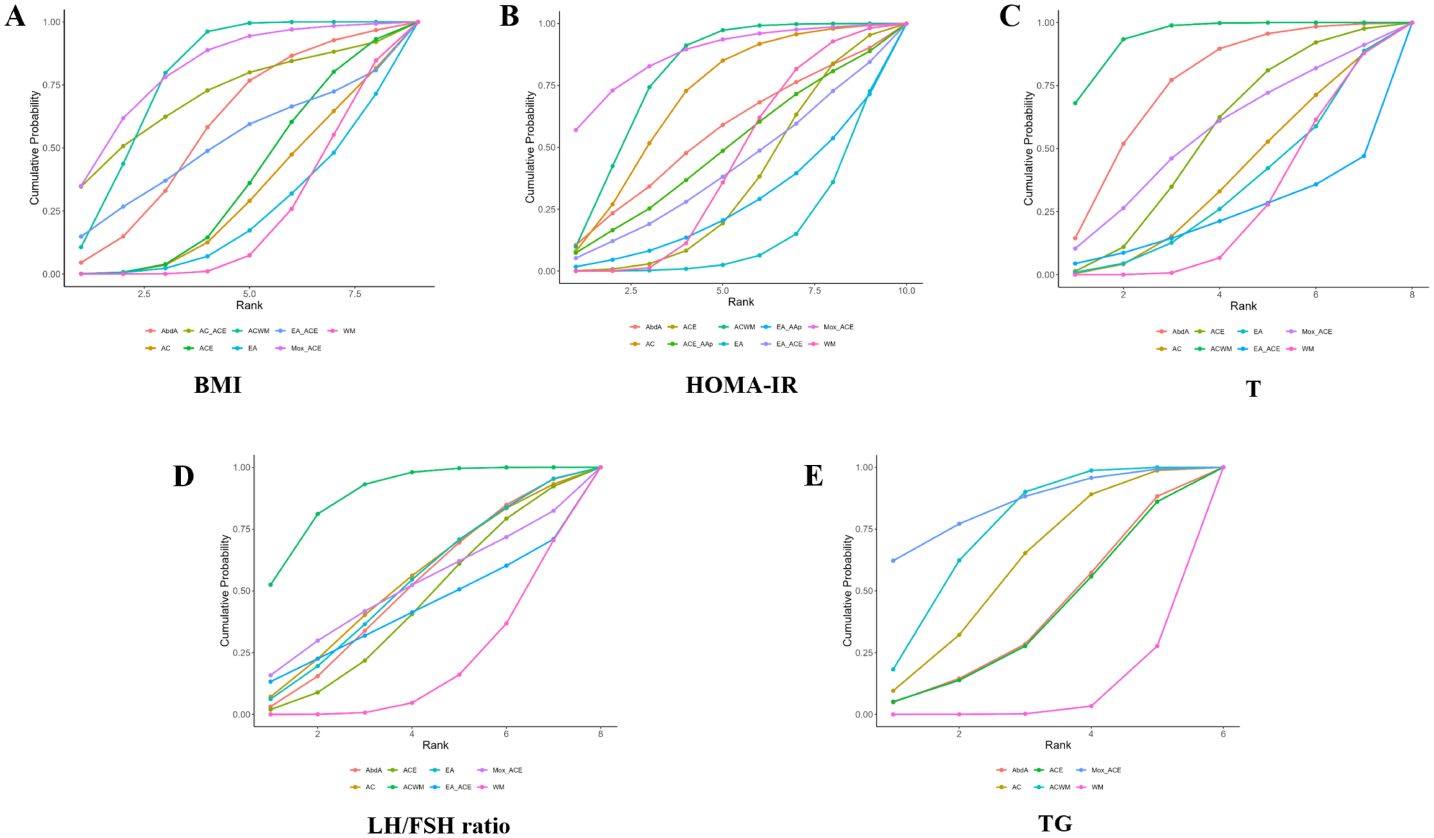
SUCRA values for all outcomes: (A) BMI, (B) HOMA-IR, (C) T, (D) LH/FSH ratio, and (E) TG. Higher SUCRA values indicate a greater probability that an intervention ranks among the most effective treatments.

**Figure 6:**
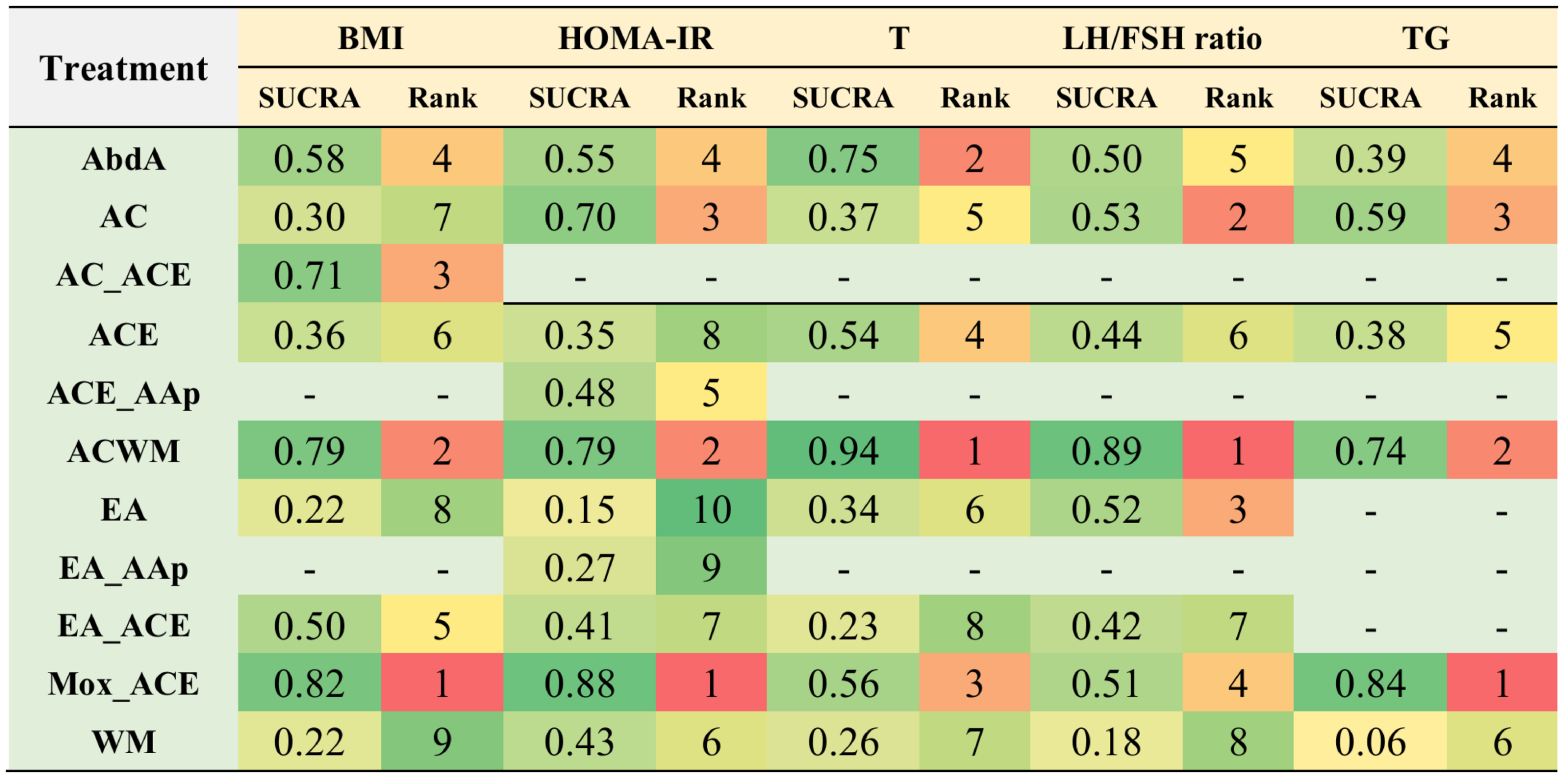
Ranking probabilities and SUCRA values for all interventions across outcomes. AbdA, abdominal acupuncture; AC, manual acupuncture; AC_ACE, acupuncture plus acupoint catgut embedding; ACE, acupoint catgut embedding; ACE_AAp, acupoint catgut embedding plus auricular acupressure; ACWM, ACUPUNCTURE COMBINED WITH MEDication; EA, electroacupuncture; EA_AAp, electroacupuncture plus auricular acupressure; EA_ACE, electroacupuncture plus acupoint catgut embedding; Mox_ACE, moxibustion plus acupoint catgut embedding; WM, western medicine.

The estimated heterogeneity parameters were *τ* = 1.59 (95% CrI: 1.20–2.16), τ^2^ ≈ 2.52, MCID = 1.69, and I^2^ = 0.6%. Funnel plots indicated no asymmetry. (Egger’s test: *p* = 0.529) (Supplementary material S5). Statistically, compared to the WM group, the Mox_ACE and ACWM groups showed greater reductions in BMI (Figure 3). Mox_ACE (MD = −2.6, 95% CrI: −5.11, −0.14), ACWM (MD = −2.17, 95% CrI: −2.94, −1.41), both exceeding the MCID (1.69). Concurrently, the ACWM group showed greater reductions in BMI compared to the following monotherapy acupuncture-related groups: ACE, AC, and EA. ACE (MD = −1.75, 95% CrI: −3.28, −0.23), AC (MD = −1.96, 95% CrI: −3.59, −0.31), EA (MD = −2.26, 95% CrI: −4.35, −0.21). Detailed results of acupuncture-related interventions for BMI are presented in Figure 4. According to SUCRA values, Mox_ACE demonstrated the highest probability of reducing BMI (82%), followed by ACWM (79%) and AC_ACE (71%). (Figures 5A, 6).

#### 3.2.4 Results for HOMA-IR

**Figure 7:**
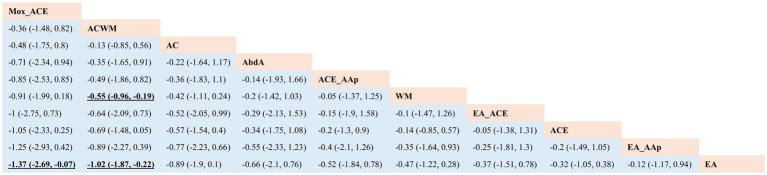
League table of relative treatment effects for HOMA-IR. Comparisons between treatments should be read from left to right, and the estimate is shown in the cell in common between the column-defining and row-defining treatments. Effect sizes are presented as MD with 95% CrIs. Mox_ACE, moxibustion plus acupoint catgut embedding; ACWM, acupuncture combined with medication; AC, manual acupuncture; AbdA, abdominal acupuncture; ACE_AAp, acupoint catgut embedding plus auricular acupressure; WM, western medicine; EA_ACE, electroacupuncture plus acupoint catgut embedding; ACE, acupoint catgut embedding; EA_AAp, electroacupuncture plus auricular acupressure; EA, electroacupuncture.

The estimated heterogeneity parameters were *τ* = 0.48 (95% CrI: 0.27–0.85); *τ*^2^ ≈ 0.23; MCID = 0.79, and *I*^2^ = 1%. Funnel plots indicated no asymmetry. (Egger’s test: *p* = 0.687) (Supplementary material S6). Compared with the WM group, the ACWM group demonstrated a statistically significant reduction in HOMA-IR (MD = −0.55, 95% CrI: −0.96, −0.19) (Figure 3), but did not exceed the MCID (0.79), suggesting limited clinical relevance. Compared with the EA group, both the Mox_ACE and ACWM groups demonstrated greater reductions in HOMA-IR: Mox_ACE (MD = −1.37, 95% CrI: −2.69, −0.07); ACWM (MD = −1.02, 95% CrI: −1.87, −0.22), both exceeding the MCID. Detailed comparative results are presented in Figure 7. Based on SUCRA values, Mox_ACE showed the highest probability of reducing HOMA-IR (88%), followed by ACWM (79%) and AC (70%) (Figures 5B, 6).

#### 3.2.5 Results for T

**Figure 8:**
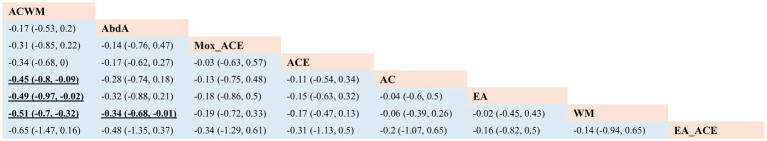
League table of relative treatment effects for T. Comparisons between treatments should be read from left to right, and the estimate is shown in the cell in common between the column-defining and row-defining treatments. Effect sizes are presented as MD with 95% CrIs. ACWM, acupuncture combined with medication; AbdA, abdominal acupuncture; Mox_ACE, moxibustion plus acupoint catgut embedding; ACE, acupoint catgut embedding; AC, manual acupuncture; EA, electroacupuncture; WM, western medicine; EA_ACE, electroacupuncture plus acupoint catgut embedding.

The estimated heterogeneity parameters were *τ* = 0.29 (95% CrI: 0.20–0.44); *τ*^2^ ≈ 0.09; MCID = 0.63, and *I*^2^ = 3%. Funnel plots indicated no asymmetry. (Egger’s test: *p* = 0.525) (Supplementary material S7). Compared with the WM group, both the ACWM group and the AbdA group showed statistically significant reductions in T levels: ACWM (MD = −0.51, 95% CrI: −0.7, −0.32); AbdA (MD = −0.34, 95% CrI: −0.68, −0.01) (Figure 3), but neither exceeded the MCID (0.63), suggesting limited clinical relevance. Additionally, the ACWM group showed greater reductions in T levels compared to the AC and EA group: AC (MD = −0.45, 95% CrI: −0.8, −0.09); EA (MD = −0.49, 95% CrI: −0.97, −0.02). Detailed comparative results are provided in Figure 8. Based on SUCRA values, ACWM showed the highest probability of reducing T (94%), followed by AbdA (75%) and Mox_ACE (56%) (Figures 5C, 6).

#### 3.2.6 Results for LH/FSH ratio

The estimated heterogeneity parameters were *τ* = 0.29 (95% CrI: 0.19–0.46); τ^2^ ≈ 0.08; MCID = 0.41, and I^2^ = 0%. Funnel plots indicated no asymmetry. (Egger’s test: *p* = 0.938) (Supplementary material S8). Compared with the WM group, the ACWM group showed a significant reduction in LH/FSH ratio (MD = −0.49, 95% CrI: −0.7, −0.27) (Figure 3), exceeding the MCID (0.41). Detailed comparative results are provided in Supplementary material S8.5. Based on SUCRA values, ACWM showed the highest probability of reducing the LH/FSH ratio (89%), followed by AC (53%) and EA (52%) (Figures 5D, 6).

#### 3.2.7 Results for TG

The estimated heterogeneity parameters were τ = 0.18 (95% CrI: 0.07–0.41); τ^2^ ≈ 0.03; MCID = 0.40, and I^2^ = 0%. Funnel plots indicated no asymmetry. (Egger’s test: *p* = 0.287) (Supplementary material S9). Compared with the WM group, the Mox_ACE group, ACWM group, and AC group showed statistically significant reductions in TG levels: Mox_ACE (MD = −0.52, 95% CrI: −0.97, −0.09); ACWM (MD = −0.4, 95% CrI: −0.64, −0.19); AC (MD = −0.33, 95% CrI: −0.61, −0.03) (Figure 3). Only the effect of Mox_ACE exceeded the MCID (0.40), while ACWM approached the threshold and AC did not reach it. Detailed comparative results are provided in Supplementary material S9.5. Based on SUCRA values, Mox_ACE demonstrated the highest probability of reducing TG levels (84%), followed by ACWM (74%) and EA (59%) (Figures 5E, 6).

#### 3.2.8 Side effects and adverse reactions of drugs

Among the 41 included studies, 11 groups reported adverse reactions or side effects (Supplementary material S10). The majority of adverse reactions associated with acupuncture and related therapies were localized bleeding or hematoma at the needle insertion sites. For the WM groups, the most common adverse reactions included gastrointestinal discomfort, headaches, hypoglycemia, and others. All symptoms were mild, and most patients tolerated them well. Three studies reported significantly lower adverse reaction rates in the experimental group compared to the control group, with statistically significant differences (*p* < 0.05).

#### 3.2.9 Confidence assessment

According to the CINeMA assessment, most pairwise comparisons were rated as having low or very low certainty of evidence, primarily due to within-study bias, imprecision, heterogeneity, and, in some cases, incoherence. Within-study bias was downgraded mainly due to limited blinding in acupuncture interventions and insufficient reporting of randomization, allocation concealment, and outcome assessment, leading to “some concerns” ratings. Imprecision and heterogeneity were downgraded when confidence or prediction intervals crossed the MCID, indicating uncertainty and variability in effect estimates. For example, imprecision for the LH/FSH ratio in the Mox_ACE vs. WM comparison was rated as “major concerns” (Supplementary material S8.9), while heterogeneity for BMI was rated as “major concerns” in the AC vs. WM and Mox vs. WM comparisons (Supplementary material S5.8). Incoherence was generally low, with no concerns for most comparisons; major concerns were observed only for TG in the AbdA vs. WM comparison due to clinically meaningful inconsistency between direct and indirect evidence (Supplementary material S9.9). Overall, based on the six CINeMA domains, the certainty of evidence was rated as low for Mox_ACE vs. WM and ACWM vs. WM across all five outcomes, except for the LH/FSH ratio in the Mox_ACE vs. WM comparison, which was rated as very low. Detailed CINeMA assessments for key comparisons are presented in Table 2.

**Table 2:** CINeMA assessment of confidence in the evidence for key comparisons.

Comparison	Outcome	Within-study bias	Reporting bias	Indirectness	Imprecision	Heterogeneity	Incoherence	Certainty
Mox_ACE vs WM	BMI	Some concerns	Low risk	No concerns	No concerns	Some concerns	No concerns	Low
	HOMA-IR	Some concerns	Low risk	No concerns	No concerns	Some concerns	No concerns	Low
	T	Some concerns	Low risk	No concerns	Some concerns	No concerns	No concerns	Low
	LH/FSH ratio	Some concerns	Low risk	No concerns	Major concerns	No concerns	No concerns	Very low
	TG	Some concerns	Low risk	No concerns	No concerns	Some concerns	No concerns	Low
ACWM vs WM	BMI	Some concerns	Low risk	No concerns	No concerns	Some concerns	No concerns	Low
	HOMA-IR	Some concerns	Low risk	No concerns	No concerns	Some concerns	No concerns	Low
	T	Some concerns	Low risk	No concerns	No concerns	Some concerns	No concerns	Low
	LH/FSH ratio	Some concerns	Low risk	No concerns	No concerns	Some concerns	No concerns	Low
	TG	Some concerns	Low risk	No concerns	No concerns	Some concerns	No concerns	Low

WM, western medicine; Mox_ACE: moxibustion plus acupoint catgut embedding; ACWM, acupuncture combined with medication.

## 4 Discussion

To our knowledge, this is the first systematic review and NMA comparing the efficacy of acupuncture and its combination therapies on endocrine and metabolic outcomes in obese patients with PCOS. It included data from 41 RCTs involving 3,500 obese PCOS patients randomly assigned to 11 different treatment regimens. The included studies were predominantly of moderate quality, with confidence assessments generally rated as low or very low. Based on the SUCRA-based rankings, our findings indicate that compared with WM, combined acupuncture interventions such as Mox_ACE and ACWM exhibited superior overall therapeutic efficacy. Mox_ACE significantly reduced BMI and metabolic parameters, including HOMA-IR and TG, relative to other treatments. Moreover, ACWM showed the best results in maintaining hormonal stability, particularly by improving T levels and the LH/FSH ratio. AC also showed notable advantages in metabolic regulation, ranking within the top three interventions for reducing HOMA-IR and TG, and achieving the second-highest ranking for improvement in the LH/FSH ratio. AbdA ranked second to ACWM in reducing T. Overall, Mox_ACE and ACWM consistently ranked among the most effective interventions across multiple outcomes, underscoring the therapeutic potential of combination acupuncture strategies. In summary, the present NMA demonstrates that combined acupuncture-based interventions offer comprehensive benefits in metabolic and endocrine modulation. These results strengthen the evidence base for the inclusion of acupuncture as an effective adjunctive therapy in the management of PCOS.

Similar to previous findings, acupuncture-related therapies can improve metabolic and hormonal levels in obese PCOS woman (25, 47), with Mox_ACE and ACWM demonstrating the most effective outcomes (32). However, this study still exhibits certain discrepancies with prior literature (24, 85). Wen et al. observed that acupuncture was less effective than metformin in improving insulin sensitivity but demonstrated superior effects on glucose metabolism and better safety. Wang et al., based on a Bayesian NMA of the general PCOS population, found that EA ranked highest for HOMA-IR improvement, whereas EA ranked relatively lower in our analysis. Several distinctions may account for these differences. The present study focused on obese women with PCOS, a subgroup characterized by more severe baseline insulin resistance and chronic inflammation, which may attenuate the short-term metabolic response to EA; In addition, our analysis categorized interventions into ten nodes, including combined therapies such asACWM and Mox_ACE, forming a more complex network structure; In contrast, Wang included fewer direct comparisons between EA and conventional medication, which may have limited network connectivity; Furthermore, the included trials in our study varied in treatment duration, stimulation frequency, and parameter settings, contributing to greater heterogeneity across studies; As HOMA-IR primarily reflects hepatic insulin sensitivity, it may not fully capture the mechanisms of EA related to autonomic regulation and enhanced skeletal-muscle glucose uptake.

Due to the lack of universally accepted MCID values for metabolic and endocrine-related indicators in PCOS, the existing literature, including the present study, has derived MCID using distribution-based methods with pooled standard deviation (SD). Compared with previous studies, the MCID values in this analysis differed (86, 87), with variations observed for T (0.43 vs. 0.63 nmol/L) and BMI (1.03 vs. 1.69 kg/m^2^). These differences likely reflect variations in population characteristics, diagnostic criteria, baseline variability, methodological approaches, intervention types, and assay methods. Standardized MCID definitions are needed to improve comparability and clinical interpretability. Given this variability, the clinical relevance of treatment effects remains difficult to interpret. To address this, the present study assessed the clinical relevance of treatment effects by comparing the magnitude of MD with predefined MCID thresholds. For BMI and the LH/FSH ratio, treatment effects consistently exceeded the MCID, indicating clinically meaningful improvements. Specifically, both Mox_ACE and ACWM showed clinically meaningful benefits for BMI, while ACWM demonstrated clinically meaningful improvement in the LH/FSH ratio. In contrast, the clinical relevance of effects for HOMA-IR and TG was inconsistent. For HOMA-IR, ACWM did not exceed the MCID (0.79) compared with WM (MD = −0.55), suggesting limited clinical benefit in this comparison and indicating that such sub-MCID effects may not translate into meaningful improvements in clinical practice. In contrast, when compared with EA, both ACWM and Mox_ACE exceeded the MCID threshold, indicating clinically meaningful improvements. This suggests that clinical relevance may be comparator-dependent. For TG, only Mox_ACE exceeded the MCID, whereas ACWM approached the threshold and AC did not reach it. This indicates that only Mox_ACE may achieve clinically meaningful benefit, while the effects of ACWM and AC may be insufficient to support changes in treatment decisions. For T levels, no interventions exceeded the MCID. Although ACWM and AbdA showed statistically significant reductions and high SUCRA rankings, these effects remained below the MCID, indicating limited clinical relevance. These findings suggest that statistical significance or SUCRA rankings alone are insufficient, as statistically significant effects may not translate into clinically meaningful or perceptible benefits. Moreover, clinical relevance may vary across pairwise comparisons, as some interventions exceeded the MCID only in specific comparisons. Overall, these findings have important implications for clinical decision-making, suggesting that statistically significant but sub-MCID effects may be insufficient to justify changes in treatment strategies. Future studies should consider adopting anchor-based MCID approaches to further validate the clinical interpretability of these findings.

Acupuncture-related therapies may exert their therapeutic effects in PCOS through several converging pathways that influence both metabolic and endocrine regulation (88, 89). These biological mechanisms correspond well with key clinical outcomes, including BMI, HOMA-IR, T, the LH/FSH ratio, and TG, and help explain the differential treatment responses observed in our NMA. Among the available modalities, EA has the most consistent mechanistic evidence. Experimental studies show that EA enhances glucose uptake and reduces ovarian insulin resistance via activation of the IRS-1/PI3K/GLUT4 pathway, and improves skeletal muscle insulin signaling through autophagy induction and inhibition of mTOR/4E-BP1 activity (90, 91). These actions collectively contribute to reductions in HOMA-IR and may also modulate sex hormone regulation, reflected in decreases in T and the LH/FSH ratio. EA-induced adipose tissue remodeling, characterized by reduced white adipose tissue, increased brown adipose tissue, and concurrent changes in gut microbiota composition, may also contribute to improvements in BMI and glucose metabolism (92). Other acupuncture-related approaches appear to act through complementary mechanisms. ACE enhances lipid oxidation through the PPAR*γ*–CPT1A pathway and may improve insulin sensitivity by regulating AMPK- and PPAR-γ–related signaling (93, 94). Moxibustion may improve metabolic dysfunction by promoting white-to-brown adipose tissue conversion via activation of the hypothalamic cAMP/PKA/CREB pathway, and by reshaping the gut microbiota–metabolite axis, thereby increasing short-chain fatty acids and enhancing insulin sensitivity (95, 96). Bo’s abdominal acupuncture has been associated with modulation of FGF21 and adipokine profiles, with potential benefits for insulin resistance and gonadotropin balance (97). Taken together, these mechanistic findings are broadly consistent with the favorable SUCRA rankings observed for acupuncture-related therapies, particularly for metabolic outcomes such as BMI, HOMA-IR, and TG. On this basis, these mechanistic and outcome specific differences provide a rationale for interpreting the clinical relevance of acupuncture related interventions from a perspective oriented toward specific treatment goals. Within a treatment-goal–oriented framework, integrating the aforementioned acupuncture-related mechanisms and the SUCRA rankings observed in this study, the potential applicability of different acupuncture-related interventions across heterogeneous PCOS populations appears biologically plausible. In particular, when treatment goals primarily involve improving insulin resistance, correcting metabolic dysfunction, supporting weight management, and preventing long term metabolic complications, Mox-ACE may be more clinically relevant, given its favorable effects on BMI, HOMA-IR, and TG. In contrast, when therapeutic objectives emphasize regulation of menstrual cycles, improvement of ovulatory function and fertility, or alleviation of hyperandrogenic manifestations such as acne and hirsutism, ACWM may offer greater potential benefit due to its effects on T and the LH/FSH ratio. Within this treatment goal oriented perspective, recent PCOS subtyping studies offer a useful clinical backdrop (98). For example, metabolic dominant presentations such as OB-PCOS may be more relevant to treatment goals addressed by Mox-ACE, whereas endocrine dominant presentations including HA-PCOS and LH-PCOS may better reflect the therapeutic focus of ACWM. However, this does not necessarily imply that these acupuncture therapies exhibit clear phenotype-specific efficacy. Overall, these interpretations are treatment goal oriented and intended to inform clinical relevance rather than establish definitive efficacy. Validation through rigorously designed large-scale multicenter RCTs with stratification by treatment goals or clinical outcomes remains necessary.

To date, few systematic reviews have comprehensively compared the therapeutic effects of acupuncture-related interventions on metabolic and endocrine hormone profiles in obese women with PCOS. Most existing investigations have primarily focused on reproductive or pregnancy-related outcomes, whereas research emphasizing metabolic and endocrine parameters remains limited (99). Nevertheless, the clinical priorities of PCOS patients evolve across the lifespan—menstrual regularity in adolescence, fertility during reproductive years, and metabolic and hormonal balance in midlife. Despite these shifting concerns, endocrine and metabolic health persist as lifelong priorities for women with PCOS. With rapid socioeconomic development, obesity has become a major public health issue, contributing not only to metabolic dysfunction but also to the exacerbation of PCOS pathophysiology. However, few systematic evaluations have targeted obese PCOS patients as a distinct subgroup. The present study bridges this gap by providing a comprehensive assessment of the efficacy and safety of acupuncture and acupuncture-related modalities in improving metabolic and endocrine profiles in this population. By incorporating a broad range of acupuncture techniques and combination treatment strategies, the analysis reflects the clinical heterogeneity of real-world practice. To minimize confounding factors, herbal medicine interventions were deliberately excluded, allowing for a more accurate assessment of the relative therapeutic effects of acupuncture-based treatments. Through a Bayesian NMA coupled with SUCRA-based ranking, the comparative advantages of different acupuncture interventions were quantitatively characterized. This approach allowed for the prioritization of various treatment modalities by integrating both direct and indirect evidence, thereby enhancing statistical power and providing robust evidence to guide individualized clinical decision-making. Sensitivity analyses using the node-splitting method within the ACWM framework confirmed the robustness and internal consistency of the network model. This approach not only verified the stability of the findings but also identified the most effective integrated intervention within the ACWM category. These insights provide valuable guidance for optimizing individualized, evidence-based therapeutic strategies for obese women with PCOS. Moreover, the limited but promising safety data suggest that acupuncture-based therapies are associated with fewer and milder adverse effects and greater patient acceptability compared with conventional pharmacotherapy, underscoring their potential as safer adjunctive or alternative options.

However, this study has several limitations. The ACWM node comprised acupuncture-related therapies combined with different pharmacological treatments, which vary in mechanisms. Due to the limited sample size, the small number of studies for certain comparisons, and the complexity of the intervention network, ACWM was not further stratified by drug class in the primary analysis. Although sensitivity analyses disaggregating ACWM showed broadly consistent rankings, this approach has methodological limitations and was not adopted as the main analytical framework. Such aggregation may introduce heterogeneity and affect comparability across studies, and may also hinder isolation of the independent effect of acupuncture, thereby yielding an uninterpretable average effect and limiting the clinical interpretability of pooled estimates and SUCRA rankings. Accordingly, findings related to ACWM should be interpreted as exploratory, representing a major limitation for the direct clinical applicability of its SUCRA rankings. Most included studies were of limited methodological quality. The lack of blinding in acupuncture-related interventions, along with imprecision and heterogeneity, contributed to a low or very low certainty of evidence. Moreover, several key comparisons did not exceed the MCID thresholds, indicating limited clinical relevance. Therefore, the findings should be interpreted with caution and require confirmation in high-quality, multicenter, and internationally collaborative trials.

## 5 Conclusion

The available evidence suggests that acupuncture and related therapies may serve as viable alternative treatment options for obese patients with PCOS, with Mox_ACE and ACWM potentially representing the most effective modalities. Compared with conventional Western pharmacotherapy, combined approaches integrating acupuncture with medication may demonstrate improved therapeutic efficacy relative to monotherapy. However, the overall quality of the existing literature remains suboptimal, with limitations including insufficient methodological rigor, inadequate assessment of the certainty of evidence, and limited clinical relevance. Therefore, these findings should be interpreted with caution. Future large-scale, high-quality RCTs are warranted to further substantiate these findings and clarify the clinical value of acupuncture-based interventions in this patient population.

## Data Availability

The original contributions presented in the study are included in the article/Supplementary material, further inquiries can be directed to the corresponding author.

## Glossary

AAp: Auricular acupressure
AbdA: Abdominal acupuncture
AC: Manual acupuncture
AC_ACE: Acupuncture plus acupoint catgut embedding
ACE: Acupoint catgut embedding
ACE_AAp: Acupoint catgut embedding plus auricular acupressure
ACWM: Acupuncture combined with medication
BMI: Body mass index
CINeMA: Confidence in network meta-analysis
CC: Clomiphene citrate
COCs: Combined oral contraceptives
DIC: Deviance information criterion
EA: Electroacupuncture
EA_AAp: Electroacupuncture plus auricular acupressure
EA_ACE: Electroacupuncture plus acupoint catgut embedding
HOMA-IR: Homeostatic model assessment of insulin resistance
LH/FSH ratio: Luteinizing hormone to follicle-stimulating hormone ratio
MD: Mean difference
Mox_ACE: Moxibustion plus acupoint catgut embedding
NMA: Network meta-analysis
PCOS: Polycystic ovary syndrome
RCTs: Randomized controlled trials
SUCRA: Surface under the cumulative ranking curve
T: Total testosterone
TCM: Traditional Chinese medicine
TG: Triglycerides
WM: Western medicine
95% CrI: 95% credible interval
