# Supplementary material for: Effects of acupuncture-related therapies on endocrine and metabolic outcomes in obese women with polycystic ovary syndrome: a systematic review and network meta-analysis
Source: Front Med (Lausanne). 2026 May 28;13:1758475. doi: 10.3389/fmed.2026.1758475 (PMC13253698; doi:10.3389/fmed.2026.1758475)
Supplement: Supplementary file 1 [file Data_Sheet_1.docx]

**Supplementary Information**

[S1 PRISMA 2020 Checklist 2](#_Toc213694336)

[S2. Search strategies 4](#_Toc213694337)

[S3. Acupoints of acupuncture groups 8](#_Toc213694338)

[S4. Risk of bias summary 14](#_Toc213694339)

[S5. Network meta-analysis of BMI outcomes 17](#_Toc213694340)

[S6. Network meta-analysis of HOMA-IR outcomes 22](#_Toc213694341)

[S7. Network meta-analysis of T outcomes 27](#_Toc213694342)

[S8. Network meta-analysis of LH/FSH ratio outcomes 32](#_Toc213694343)

[S9. Network meta-analysis of TG outcomes 37](#_Toc213694344)

[S10 Side Effects and Adverse Reactions of Drugs 42](#_Toc213694345)

# S1 PRISMA 2020 Checklist

| **Section and Topic** | **Item #** | **Checklist item** | **Location where item is reported** |
| --- | --- | --- | --- |
| **TITLE** | | |  |
| Title | 1 | Identify the report as a systematic review. | 1 |
| **ABSTRACT** | | |  |
| Abstract | 2 | See the PRISMA 2020 for Abstracts checklist. | 1 |
| **INTRODUCTION** | | |  |
| Rationale | 3 | Describe the rationale for the review in the context of existing knowledge. | 2 |
| Objectives | 4 | Provide an explicit statement of the objective(s) or question(s) the review addresses. | 2-3 |
| **METHODS** | | |  |
| Eligibility criteria | 5 | Specify the inclusion and exclusion criteria for the review and how studies were grouped for the syntheses. | 3 |
| Information sources | 6 | Specify all databases, registers, websites, organisations, reference lists and other sources searched or consulted to identify studies. Specify the date when each source was last searched or consulted. | 3 |
| Search strategy | 7 | Present the full search strategies for all databases, registers and websites, including any filters and limits used. | 4 |
| Selection process | 8 | Specify the methods used to decide whether a study met the inclusion criteria of the review, including how many reviewers screened each record and each report retrieved, whether they worked independently, and if applicable, details of automation tools used in the process. | 4 |
| Data collection process | 9 | Specify the methods used to collect data from reports, including how many reviewers collected data from each report, whether they worked independently, any processes for obtaining or confirming data from study investigators, and if applicable, details of automation tools used in the process. | 4 |
| Data items | 10a | List and define all outcomes for which data were sought. Specify whether all results that were compatible with each outcome domain in each study were sought (e.g. for all measures, time points, analyses), and if not, the methods used to decide which results to collect. | 4 |
|  | 10b | List and define all other variables for which data were sought (e.g. participant and intervention characteristics, funding sources). Describe any assumptions made about any missing or unclear information. | 4 |
| Study risk of bias assessment | 11 | Specify the methods used to assess risk of bias in the included studies, including details of the tool(s) used, how many reviewers assessed each study and whether they worked independently, and if applicable, details of automation tools used in the process. | 4 |
| Effect measures | 12 | Specify for each outcome the effect measure(s) (e.g. risk ratio, mean difference) used in the synthesis or presentation of results. | 3 |
| Synthesis methods | 13a | Describe the processes used to decide which studies were eligible for each synthesis (e.g. tabulating the study intervention characteristics and comparing against the planned groups for each synthesis (item #5)). | 3 |
|  | 13b | Describe any methods required to prepare the data for presentation or synthesis, such as handling of missing summary statistics, or data conversions. | 4 |
|  | 13c | Describe any methods used to tabulate or visually display results of individual studies and syntheses. | 4 |
|  | 13d | Describe any methods used to synthesize results and provide a rationale for the choice(s). If meta-analysis was performed, describe the model(s), method(s) to identify the presence and extent of statistical heterogeneity, and software package(s) used. | 4 |
|  | 13e | Describe any methods used to explore possible causes of heterogeneity among study results (e.g. subgroup analysis, meta-regression). | 4 |
|  | 13f | Describe any sensitivity analyses conducted to assess robustness of the synthesized results. | 4 |
| Reporting bias assessment | 14 | Describe any methods used to assess risk of bias due to missing results in a synthesis (arising from reporting biases). | 4 |
| Certainty assessment | 15 | Describe any methods used to assess certainty (or confidence) in the body of evidence for an outcome. | 4 |
| **RESULTS** | | |  |
| Study selection | 16a | Describe the results of the search and selection process, from the number of records identified in the search to the number of studies included in the review, ideally using a flow diagram. | 5 |
|  | 16b | Cite studies that might appear to meet the inclusion criteria, but which were excluded, and explain why they were excluded. | 5 |
| Study characteristics | 17 | Cite each included study and present its characteristics. | 6-8 |
| Risk of bias in studies | 18 | Present assessments of risk of bias for each included study. | 5 |
| Results of individual studies | 19 | For all outcomes, present, for each study: (a) summary statistics for each group (where appropriate) and (b) an effect estimate and its precision (e.g. confidence/credible interval), ideally using structured tables or plots. | 9-12 |
| Results of syntheses | 20a | For each synthesis, briefly summarise the characteristics and risk of bias among contributing studies. | 9-12 |
|  | 20b | Present results of all statistical syntheses conducted. If meta-analysis was done, present for each the summary estimate and its precision (e.g. confidence/credible interval) and measures of statistical heterogeneity. If comparing groups, describe the direction of the effect. | 9-12 |
|  | 20c | Present results of all investigations of possible causes of heterogeneity among study results. | 9 |
|  | 20d | Present results of all sensitivity analyses conducted to assess the robustness of the synthesized results. | 9 |
| Reporting biases | 21 | Present assessments of risk of bias due to missing results (arising from reporting biases) for each synthesis assessed. | 9 |
| Certainty of evidence | 22 | Present assessments of certainty (or confidence) in the body of evidence for each outcome assessed. | 12 |
| **DISCUSSION** | | |  |
| Discussion | 23a | Provide a general interpretation of the results in the context of other evidence. | 13 |
|  | 23b | Discuss any limitations of the evidence included in the review. | 15 |
|  | 23c | Discuss any limitations of the review processes used. | 15 |
|  | 23d | Discuss implications of the results for practice, policy, and future research. | 14 |
| **OTHER INFORMATION** | | |  |
| Registration and protocol | 24a | Provide registration information for the review, including register name and registration number, or state that the review was not registered. | 15 |
|  | 24b | Indicate where the review protocol can be accessed, or state that a protocol was not prepared. | 15 |
|  | 24c | Describe and explain any amendments to information provided at registration or in the protocol. | 15 |
| Support | 25 | Describe sources of financial or non-financial support for the review, and the role of the funders or sponsors in the review. | 15 |
| Competing interests | 26 | Declare any competing interests of review authors. | 15 |
| Availability of data, code and other materials | 27 | Report which of the following are publicly available and where they can be found: template data collection forms; data extracted from included studies; data used for all analyses; analytic code; any other materials used in the review. | 15 |

# S2. Search strategies

**PubMed**

**#1:**

(Polycystic Ovary Syndrome[MeSH Terms]) OR (Polycystic Ovarian Syndrome[Title/Abstract] OR Sclerocystic Ovarian Degeneration[Title/Abstract] OR Sclerocystic Ovary Syndrome[Title/Abstract] OR Stein-Leventhal Syndrome[Title/Abstract] OR Stein Leventhal Syndrome[Title/Abstract] OR Sclerocystic Ovaries[Title/Abstract] OR Sclerocystic Ovary[Title/Abstract] OR polycystic ovary[Title/Abstract] OR PCOS[Title/Abstract] OR PCOD[Title/Abstract])

#2

(acupuncture[MeSH Terms] OR Pharmacopuncture[Title/Abstract] OR acupuncture therapy[MeSH Terms] OR "Acupuncture Treatment*"[Title/Abstract] OR Pharmacoacupuncture Treatment[Title/Abstract] OR Pharmacoacupuncture Therapy[Title/Abstract] OR Acupotom*[Title/Abstract] OR acupuncture points[MeSH Terms] OR Acupuncture Point[Title/Abstract] OR Acupoints[Title/Abstract] OR Acupoint[Title/Abstract] OR acupressure[MeSH Terms] OR Shiatsu[Title/Abstract] OR Zhi Ya[Title/Abstract] OR Chih Ya[Title/Abstract] OR moxibustion[MeSH Terms] OR Moxabustion[Title/Abstract] OR acupuncture, ear[MeSH Terms] OR Ear Acupuncture*[Title/Abstract] OR "Auricular Acupuncture*"[Title/Abstract] OR Electroacupuncture[Title/Abstract] OR electro-acupuncture[Title/Abstract] OR electric acupuncture[Title/Abstract] OR Needling[Title/Abstract] OR acupoint embedding[Title/Abstract] OR catgut embedding[Title/Abstract] OR body acupuncture[Title/Abstract] OR warm needling[Title/Abstract] OR fire needling[Title/Abstract] OR scalp acupuncture[Title/Abstract] OR plum blossom needle[Title/Abstract] OR Acupuncture Analgesia[Title/Abstract] OR Acupuncture Anesthesia[Title/Abstract] OR "acupoint injection"[Title/Abstract] OR "acupoint moxibustion" [Title/Abstract] OR acupoint catgut embedding[Title/Abstract])

#3

"Randomized Controlled Trials as Topic"[Mesh] OR (randomized controlled trial[Publication Type]) OR (controlled clinical trial[Publication Type]) OR (randomized controlled trial[Title/Abstract]) OR (controlled clinical trial[Title/Abstract]) OR (RCT[Title/Abstract]) OR (randomized[Title/Abstract]) OR (clinical trials[Title/Abstract]) OR (placebo[Title/Abstract]) OR (randomly[Title/Abstract]) OR (trial[Title/Abstract]) OR (groups[Title/Abstract])

**#1 AND #2 AND #3**

N=180

**Web of Science**

#1

TS=("Polycystic Ovary Syndrome" OR "Polycystic Ovary Syndromes" OR "Polycystic Ovarian Syndrome" OR "Polycystic Ovarian Disease" OR "Sclerocystic Ovarian Degeneration" OR "Sclerocystic Ovary Syndrome" OR "Stein-Leventhal Syndrome" OR "Stein Leventhal Syndrome" OR "Sclerocystic Ovaries" OR "Sclerocystic Ovary" OR "polycystic ovary" OR PCOS OR PCOD)

#2

TS=(acupuncture OR Pharmacopuncture OR Acup OR "acupuncture therapy" OR "Acupuncture Treatment*" OR "Pharmacoacupuncture Treatment" OR "Pharmacoacupuncture Therapy" OR Acupotom* OR "acupuncture points" OR "Acupuncture Point" OR Acupoints OR Acupoint OR acupressure OR Shiatsu OR "Zhi Ya" OR "Chih Ya" OR moxibustion OR Mox OR "Ear Acupuncture*" OR "Auricular Acupuncture*" OR Electroacupuncture OR electro-acupuncture OR electric acupuncture OR Needling OR "acupoint embedding" OR “catgut embedding” OR "body acupuncture" OR "warm needling" OR "fire needling" OR "scalp acupuncture" OR "plum blossom needle" OR "Acupuncture Analgesia" OR "Acupuncture Anesthesia" OR "acupoint injection" OR "acupoint moxibustion")

#3

TS=(“randomized controlled trial” OR “controlled clinical trial” OR RCT OR randomized OR clinical trials OR placebo OR randomly OR trial OR groups)

**#1 AND #2 AND #3**

N=318

**EMBASE**

#1:

('ovary polycystic disease'/exp OR 'cystic ovary':ab,ti OR 'micropolycystic ovary':ab,ti OR 'multiple follicle cyst':ab,ti OR 'ovary polycystic syndrome':ab,ti OR 'ovary, micropolycystic':ab,ti OR 'ovary, polycystic':ab,ti OR 'polycystic ovarian disease':ab,ti OR 'polycystic ovary':ab,ti OR 'polycystic ovary disease':ab,ti OR 'polycystic ovary syndrome':ab,ti OR 'stein cohen leventhal syndrome':ab,ti OR 'stein leventhal disease':ab,ti OR 'stein leventhal syndrome':ab,ti OR 'syndrome stein leventhal':ab,ti OR 'ovary polycystic disease':ab,ti OR 'PCOS':ab,ti OR 'PCOD':ab,ti)

#2

('acupuncture'/exp OR 'acupuncture':ab,ti OR 'acupuncture therapy':ab,ti OR 'pharmacopuncture'/exp OR 'pharmacopuncture':ab,ti OR 'pharmacoacupuncture':ab,ti OR 'pharmaco-acupuncture':ab,ti OR 'pharmaco-puncture':ab,ti OR 'herbal acupuncture':ab,ti OR 'herb acupuncture':ab,ti OR 'acupotomy'/exp OR 'acupotomy':ab,ti OR 'needle knife':ab,ti OR 'needle-knife therapy':ab,ti OR 'small needle-knife therapy':ab,ti OR 'acupuncture point'/exp OR 'acupuncture point':ab,ti OR 'acupoint':ab,ti OR 'acupoints':ab,ti OR 'acupuncture points':ab,ti OR 'acupressure'/exp OR 'acupressure':ab,ti OR 'moxibustion'/exp OR 'moxibustion':ab,ti OR 'auricular acupuncture'/exp OR 'auricular acupuncture':ab,ti OR 'ear acupuncture':ab,ti OR 'auriculotherapy':ab,ti OR 'electroacupuncture'/exp OR 'electroacupuncture':ab,ti OR 'electro-acupuncture':ab,ti OR 'electrical acupuncture':ab,ti OR 'electrical acupoint stimulation':ab,ti OR 'acupuncture analgesia'/exp OR 'acupuncture analgesia':ab,ti OR 'acupuncture anesthesia':ab,ti OR 'acupuncture anaesthesia':ab,ti OR 'acupoint embedding':ab,ti OR 'warm needling':ab,ti OR 'fire needling':ab,ti OR 'fire needle':ab,ti OR 'scalp acupuncture':ab,ti OR 'plum blossom needle':ab,ti OR 'catgut embedding':ab,ti OR 'acupoint injection':ab,ti OR 'catgut embedding'/exp OR 'ACET (acupoint catgut embedding therapy)' OR 'acupoint catgut embedding therapy' OR 'catgut implantation' OR 'catgut embedding')

#3

'randomized controlled trial'/exp OR 'randomized controlled trial':ab,ti OR 'controlled trial, randomized':ab,ti OR 'randomised controlled study':ab,ti OR 'randomised controlled trial':ab,ti OR 'randomized controlled study':ab,ti OR 'trial, randomized controlled':ab,ti OR 'randomized controlled trial':ab,ti OR 'rct' OR 'randomized' OR 'clinical trials'/exp OR 'clinical trials' OR 'placebo'/exp OR 'placebo' OR 'randomly' OR 'trial'/exp OR 'trial' OR 'groups'

**#1 AND #2 AND #3**

**N=346**

**Cochrane libraray**

#1 MeSH descriptor: [Polycystic Ovary Syndrome] explode all trees

#2 (Stein-Leventhal Syndrome):ti,ab,kw OR (Stein Leventhal Syndrome):ti,ab,kw OR (Polycystic Ovary Syndrome 1):ti,ab,kw OR (Polycystic Ovarian Syndrome):ti,ab,kw OR (Ovary Syndrome, Polycystic):ti,ab,kw OR (Ovarian Degeneration, Sclerocystic):ti,ab,kw OR (Sclerocystic Ovary Syndrome):ti,ab,kw OR (Syndrome, Polycystic Ovary):ti,ab,kw OR (Syndrome, Stein-Leventhal):ti,ab,kw OR (Ovarian Syndrome, Polycystic):ti,ab,kw OR (Sclerocystic Ovarian Degeneration):ti,ab,kw OR (Sclerocystic Ovary):ti,ab,kw OR (Ovary, Sclerocystic):ti,ab,kw OR (Sclerocystic Ovaries):ti,ab,kw OR (PCOS):ti,ab,kw OR (PCOD):ti,ab,kw

#3 #1 OR #2

#4 MeSH descriptor: [Acupuncture] explode all trees

#5 (Pharmacopuncture):ti,ab,kw

#6 MeSH descriptor: [Acupuncture Therapy] explode all trees

#7 (Acupotomy):ti,ab,kw OR (Acupotomies):ti,ab,kw OR (Treatment, Acupuncture):ti,ab,kw OR (Therapy, Acupuncture):ti,ab,kw OR (Acupuncture Treatment):ti,ab,kw OR (Acupuncture Treatments):ti,ab,kw OR (Therapy, Pharmacoacupuncture):ti,ab,kw OR (Pharmacoacupuncture Therapy):ti,ab,kw OR (Treatment, Pharmacoacupuncture):ti,ab,kw OR (Pharmacoacupuncture Treatment):ti,ab,kw

#8 MeSH descriptor: [Acupuncture Points] explode all trees

#9 (Acupoint):ti,ab,kw OR (Point, Acupuncture):ti,ab,kw OR (Acupuncture Point):ti,ab,kw OR (Points, Acupuncture):ti,ab,kw OR (Acupoints):ti,ab,kw 12308

#10 MeSH descriptor: [Acupressure] explode all trees

#11 (Zhi Ya):ti,ab,kw OR (Chih Ya):ti,ab,kw OR (Shiatzu):ti,ab,kw OR (Shiatsu):ti,ab,kw

#12 MeSH descriptor: [Moxibustion] explode all trees

#13 (Moxabustion):ti,ab,kw

#14 MeSH descriptor: [Acupuncture, Ear] explode all trees

#15 (Acupunctures, Auricular):ti,ab,kw OR (Acupuncture, Auricular):ti,ab,kw OR (Auricular Acupuncture):ti,ab,kw OR (Auricular Acupunctures):ti,ab,kw OR (Ear Acupuncture):ti,ab,kw OR (Ear Acupunctures):ti,ab,kw OR (Acupunctures, Ear):ti,ab,kw

#16 MeSH descriptor: [Electroacupuncture] explode all trees

#17 (electro-acupuncture):ti,ab,kw OR (electric acupuncture):ti,ab,kw OR electroacupuncture

#18 MeSH descriptor: [Acupuncture Analgesia] explode all trees

#19 (Acupuncture Anesthesia):ti,ab,kw OR (Anesthesia, Acupuncture):ti,ab,kw OR (Analgesia, Acupuncture):ti,ab,kw

#20 (Needling):ti,ab,kw OR (acupoint embedding):ti,ab,kw OR (catgut embedding):ti,ab,kw OR (body acupuncture):ti,ab,kw OR (warm needling):ti,ab,kw OR (fire needling):ti,ab,kw OR (scalp acupuncture):ti,ab,kw OR (plum blossom needle):ti,ab,kw OR (acupoint injection):ti,ab,kw OR (acupoint moxibustion):ti,ab,kw

#21 #4 OR #5 OR #6 OR #7 OR #8 OR #9 OR #10 OR #11 OR #12 OR #13 OR #14 OR #15 OR #16 OR #17 OR #18 OR #19 OR #20 25716

#22 (randomized controlled trial):ti,ab,kw OR (controlled clinical trial):ti,ab,kw OR (RCT):ti,ab,kw OR (Randomized):ti,ab,kw OR (clinical trials):ti,ab,kw OR (placebo):ti,ab,kw OR (randomly):ti,ab,kw OR (trial):ti,ab,kw OR (groups):ti,ab,kw

**#23 #3 AND #21 AND #22**

**N=181**

**Sinomed**

("随机对照试验"[常用字段:智能] OR "随机对照研究"[常用字段:智能] OR "RCT"[常用字段:智能] OR "随机"[常用字段:智能] OR "对照"[常用字段:智能]) AND ("多囊卵巢综合征"[常用字段:智能] OR "多囊"[常用字段:智能] OR "PCOS"[常用字段:智能]) AND ("针刺"[常用字段:智能] OR "针灸"[常用字段:智能] OR "针"[常用字段:智能] OR "穴位"[常用字段:智能] OR "灸"[常用字段:智能] OR "电针"[常用字段:智能] OR "耳针"[常用字段:智能] OR "体针"[常用字段:智能] OR "温针"[常用字段:智能] OR "火针"[常用字段:智能] OR "头针"[常用字段:智能] OR "梅花针"[常用字段:智能] OR "针刺镇痛"[常用字段:智能] OR "埋线"[常用字段:智能] OR "穴位注射"[常用字段:智能])

**N=981**

**CNKI**

SU=（多囊卵巢综合征+多囊+PCOS） AND SU=（针刺+针灸+针+穴位+灸+电针+耳针+耳穴+体针+温针+火针+头针+梅花针+针灸镇痛+埋线+穴位注射） AND AB=（随机对照试验+随机对照研究+RCT+随机+对照）

**N=727**

**Wanfang**

主题:( 多囊卵巢综合征 or多囊 or PCOS ) and 主题:(针刺 or 针灸 or 针 or 穴位or 灸or 电针 or 耳针 or 耳穴or 体针or 温针灸or 火针 or 头针or梅花针or针灸镇痛or 埋线 or 穴位注射) and 摘要:(随机对照试验 or 随机对照研究 or RCT or 随机 or 对照 )

**N=553**

**VIP**

M=(多囊卵巢综合征 or 多囊 or PCOS) and M=(针刺 or 针灸 or 针 or 穴位or 灸or 电针 or 耳针 or 耳穴or 体针or 温针灸or 火针 or 头针or梅花针or针灸镇痛or 埋线 or 穴位注射) and R= (随机对照试验 or 随机对照研究 or RCT or 随机 or 对照 )

**N=704**

# S3. Acupoints of acupuncture groups

| **Study** | **Acupoint** | **Detailed Intervention Procedure** | **Acupuncture Frequency** |
| --- | --- | --- | --- |
| Yan-HuaZheng 2013 | CV4 (Guanyuan)、CV6 (Qihai)、CV10 (Xiawan)、CV12 (Zhongwan)、ST21(Liangmen)、ST25 (Tianshu)、ST28 (Shuidao) | Disposable, single-use, sterilized needles made of stainless steel were inserted to a depth of 15–30 mm in segmental acupuncture points located in abdominal muscles with innervations corresponding to the ovaries; conception vessel and stomach meridian bilaterally. All needles were retained manually (de qi) once inserted and remained in place for 30 minutes at each treatment. | once a day |
| Jie Cao2023 | NA | NA | 3 times a week |
| Raden Muharam2022 | CV3 (Zhongji), CV4 (Guanyuan), CV6 (Qihai), ST25 (Tianshu), ST28 (Shuidao), ST36 (Zusanli), BL57 (Chengsan), SP6 (Sanyinjiao bilateral) | The acupoints were punctured and stimulated with a continuous wave at a frequency of 2 Hz. The intensity was adjusted to the patient's comfort level, and the stimulation lasted for 30 minutes. The electroacupuncture (EA) needles were connected to an SDZ-V nerve and muscle electrostimulator. | 3 times a week |
| Tianyu Wu2024 | The first acupoint formula comprises GV20 (Baihui), GV24 (Shenting), GB13 (Benshen), CV12 (Zhongwan), ST25 (Tianshu), CV4 (Guanyuan), EX-CA1 (Zigong), KI12 (Dahe), SP6 (Sanyinjiao), and LR3 (Taichong). The second acupoint formula comprises BL23 (Shenshu), BL32 (Ciliao), SP6 (Sanyinjiao), and KI3 (Taixi). | Disposable, single-use, sterilized needles of sizes 0.25mm × 25mm, 0.25mm × 40mm, and 0.25mm × 50mm will be inserted into the acupoints, and a Deqi sensation obtained by manipulating the needles. Each treatment will last for 30 min; no manipulation will be used once the Deqi sensation is achieved. | 2-3 times a week |
| Yue Jin2020 | In the supine position, the selected acupoints include:CV4 (Guanyuan), CV3 (Zhongji), ST25 (Tianshu, bilateral), SP15 (Daheng, bilateral), ST36 (Zusanli, bilateral), and SP6 (Sanyinjiao, bilateral).  In the prone position, the selected acupoints include:BL20 (Pishu, bilateral), BL23 (Shenshu, bilateral), BL26 (Guanyuanshu, bilateral), and BL32 (Ciliao, bilateral). | Each treatment session begins with acupoint therapy in the supine position, followed by acupoint therapy in the prone position.After obtaining Deqi using the routine needle insertion technique, apply the even reinforcing-reducing method (ping bu ping xie).Retain the needle for 30 minutes. During needle retention, perform gentle moxibustion on SP6 combined with either ST25 or BL26 for 15 minutes, alternating between Tianshu and Guanyuan Shu each session. | once a day |
| Yue Jin2021 | In the supine position, the selected acupoints include:CV4 (Guanyuan), CV3 (Zhongji), ST25 (Tianshu, bilateral), SP15 (Daheng, bilateral), ST36 (Zusanli, bilateral), SP6 (Sanyinjiao, bilateral)  In the prone position, the selected acupoints include:BL20 (Pishu, bilateral), BL23 (Shenshu, bilateral), BL26 (Guanyuanshu, bilateral), BL32 (Ciliao, bilateral) | Patients were treated in both supine and prone positions, with the two posture groups alternating treatment on alternate days. After routine disinfection of the selected acupoints, filiform needles of 1.5 cun (0.25 mm × 25–40 mm) were inserted. Following needle insertion and the arrival of deqi, a neutral reinforcing-reducing manipulation technique was applied. The needles were retained for 30 minutes. | once a day |
| Zhang Zeli 2016 | CV3 (Zhongji), CV4 (Guanyuan), EX-CA1 (Zigong), SP6 (Sanyinjiao), KI7 (Fuliu), ST36 (Zusanli), KI13 (Qixue) | Acupuncture was performed at the designated acupoints. Needles were inserted perpendicularly or obliquely to a depth of 4-5 mm until the patient experienced a sensation of distension, tightness, or heaviness (deqi). The needles were retained for 30 minutes, and a reinforcing manipulation technique achieved by rotating and twirling the needle was applied every 10 minutes to reinforce the needling sensation. | once a day |
| Yu Xiaoli2023 | CV4 (Guanyuan), CV6 (Qihai), BL23 (Shenshu), BL20 (Pishu), BL32 (Ciliao), ST40 (Fenglong), SP6 (Sanyinjiao), ST36 (Zusanli) | Following needle insertion and the arrival of deqi, the needles were retained for 30 minutes. | once every two days |
| Wang Jiali 2009 | AC:Main Acupoints:ST21 (Liangmen, bilateral), ST25 (Tianshu, bilateral), GB26 (Daimai, bilateral), ST29 (Guilai, bilateral), SP10 (Xuehai, bilateral), SP6 (Sanyinjiao, bilateral);Adjunctive Acupoints:SP9 (Yinlingquan), LI11 (Quchi), TE6 (Zhigou), ST44 (Neiting), LR3 (Taichong), KI7 (Fuliu), KI3 (Taixi), selected based on the patient's condition.  ACE:Main Acupoints: CV12 (Zhongwan), ST25 (Tianshu, bilateral), CV6 (Qihai), ST37 (Shangjuxu,bilateral);Adjunctive Acupoints:BL20 (Pishu), BL25 (Dachangshu), BL18 (Ganshu), BL20 (Pishu), BL23 (Shenshu), selected based on the patient's condition. | AC:After routine disinfection of the acupoints, disposable sterile acupuncture needles were used for rapid insertion. Upon the arrival of deqi, the reducing method was applied for excess syndrome patterns, while the reinforcing method was applied for deficiency syndrome patterns.  ACE: After strict aseptic disinfection of the selected acupoint areas and the operator's hands, the catgut suture was placed into the needle tube using a hemostat. The disposable injection needle was then inserted into the acupoint to a depth of approximately 1.5 cm. After the arrival of deqi, the stylet was pushed to embed the catgut suture into the acupoint tissue. The needle was subsequently withdrawn, and the puncture point was pressed with a sterile dry cotton ball for a moment to prevent bleeding. | AC: once two days;  ACE: once ten days |
| He Danjuan  2020 | Group 1:BL18 (Ganshu), CV3 (Zhongji), BL17 (Geshu), ST36 (Zusanli), SP6 (Sanyinjiao), GB26 (Daimai), CV4 (Guanyuan)  Group 2: BL23 (Shenshu), BL20 (Pishu), ST25 (Tianshu), CV9 (Shuifen), SP9 (Yinlingquan), ST40 (Fenglong), EX-CA2 (Luanchao) | The patient was placed in the supine position. After disinfecting the local skin around the acupoints with povidone-iodine, the catheter needle embedding method was employed. An absorbable surgical suture was loaded into the tip of the catheter needle, followed by attachment of the stylet. The operator stretched the skin around the acupoint with the left hand and swiftly inserted the embedding needle with the right hand. Upon the arrival of deqi, the stylet was advanced gently while simultaneously withdrawing the needle sleeve, leaving the suture embedded within the acupoint. | once a week |
| Yan Huili2023 | Group A:BL27 (Xiaochangshu), ST25 (Tianshu), LR13 (Zhangmen), CV4 (Guanyuan), ST36 (Zusanli), SP6 (Sanyinjiao)  Group B:BL21 (Weishu), BL23 (Shenshu), CV12 (Zhongwan), CV9 (Shuifen), GB25 (Jingmen), EX-CA2 (Luanchao) | Disposable intradermal suture needles and medically absorbable sutures were used. The suture was loaded into the needle. The operator stretched the skin at the puncture site with the thumb and index finger of the left hand, while swiftly inserting the needle with the right hand to accurately reach the acupoint. Upon the patient's report of a deqi sensation, the needle was withdrawn slowly, leaving the suture embedded within the acupoint. | Every15~20 day |
| Su Ting2025 | Group A:BL18 (Ganshu, bilateral), CV3 (Zhongji), BL17 (Geshu, bilateral), ST36 (Zusanli, bilateral), SP6 (Sanyinjiao, bilateral), GB26 (Daimai, bilateral), CV4 (Guanyuan)  Group B:EX-CA2 (Luanchao, bilateral), ST40 (Fenglong, bilateral), SP9 (Yinlingquan, bilateral), CV9 (Shuifen), ST25 (Tianshu, bilateral), BL20 (Pishu, bilateral), BL23 (Shenshu, bilateral) | The two treatment groups were administered alternately. Following disinfection, a 1 cm segment of absorbable suture was loaded into the tip of a hollow needle. After selecting the appropriate acupoint, the needle was inserted slowly. Upon the arrival of deqi, the stylet was pushed forward while the needle sleeve was withdrawn slowly, leaving the suture embedded between the subcutaneous tissue and the muscle layer. The needle was then completely removed. The puncture site was compressed with a sterile cotton ball for hemostasis, and an adhesive bandage was applied. | Mox: once ten days;  ACE: once five days |
| Wang Limei  2025 | Main Acupoints:CV12 (Zhongwan), ST25 (Tianshu, bilateral), ST40 (Fenglong), SP9 (Yinlingquan), SP6 (Sanyinjiao)  Adjunctive Acupoints:CV3 (Zhongji), BL20 (Pishu, bilateral), SP15 (Daheng), ST36 (Zusanli), ST29 (Guilai), GB26 (Daimai) | The patient was placed in a supine position. The skin at the acupoints was disinfected with povidone-iodine. Using the catheter needle embedding technique, a disposable embedding needle was employed. An absorbable suture was loaded into the tip of the needle, followed by placement of the stylet. The skin around the acupoint was stretched taut, and the embedding needle was swiftly inserted. After the patient experienced deqi, the stylet was advanced slowly while the needle sleeve was withdrawn simultaneously, leaving the absorbable suture embedded within the acupoint. | once two weeks |
| Ma Guizhi  2020 | BL20 (Pishu), BL23 (Shenshu), ST25 (Tianshu), SP9 (Yinlingquan), ST40 (Fenglong), CV9 (Shuifen), ST36 (Zusanli), SP6 (Sanyinjiao), CV4 (Guanyuan), GB26 (Daimai) | After local disinfection of the acupoints, a No. 7 disposable intradermal suture needle and absorbable collagen suture were selected. The collagen suture was threaded into the needle, which was then swiftly inserted through the skin at the aforementioned acupoints. Upon the arrival of deqi, the stylet was slowly advanced while the needle sleeve was withdrawn, leaving the suture embedded. On the day of the procedure, the acupoint sites were kept dry to avoid contact with water. | once ten days |
| He Danjuan  2020 | Group 1:BL18 (Ganshu, bilateral), CV3 (Zhongji), BL17 (Geshu, bilateral), ST36 (Zusanli, bilateral), SP6 (Sanyinjiao, bilateral), GB26 (Daimai, bilateral), CV4 (Guanyuan)  Group 2:BL23 (Shenshu, bilateral), BL20 (Pishu, bilateral), ST25 (Tianshu, bilateral), CV9 (Shuifen), SP9 (Yinlingquan, bilateral), ST40 (Fenglong, bilateral), EX-CA2 (Luanchao, bilateral) | For each treatment, a single set of acupoints was selected, with the two groups alternating between sessions. The patient was placed in a supine position, and the local skin around the acupoints was disinfected with povidone-iodine. Using the catheter needle embedding method, an absorbable surgical suture was placed into the tip of the catheter needle, followed by connecting the stylet. The operator stretched the skin around the acupoint with the left hand and swiftly inserted the embedding needle with the right hand. After the patient experienced deqi, the stylet was pushed gently while simultaneously withdrawing the needle sleeve, leaving the suture embedded within the acupoint. | once a week |
| Zhang Jianfeng  2016 | CV3 (Zhongji), CV4 (Guanyuan), CV12 (Zhongwan), ST36 (Zusanli), ST25 (Tianshu), SP15 (Daheng), ST40 (Fenglong), BL25 (Dachangshu), ST28 (Shuidao), BL21 (Weishu), BL20 (Pishu) | After routine local disinfection of the acupoints, a 3–0 gauge catgut suture (1.5 cm in length) was loaded into the lumen of a disposable No. 8 needle. The needle was then inserted into the corresponding acupoint. Upon the arrival of deqi, the stylet was slowly advanced while simultaneously withdrawing the needle sleeve, thereby embedding the catgut suture into the tissue. | once fifteen days |
| Chen Li2018 | Group 1:BL18 (Ganshu, bilateral), CV3 (Zhongji), BL17 (Geshu, bilateral), ST36 (Zusanli, bilateral), SP6 (Sanyinjiao, bilateral), GB26 (Daimai, bilateral), CV4 (Guanyuan)  Group 2:BL23 (Shenshu, bilateral), BL20 (Pishu, bilateral), ST25 (Tianshu, bilateral), CV9 (Shuifen), SP9 (Yinlingquan, bilateral), ST40 (Fenglong, bilateral), EX-CA2 (Luanchao, bilateral) | For each treatment session, a single set of acupoints was selected, with the two sets alternated consecutively. After routine local disinfection, a sterilized 3-0 catgut suture (approximately 7 cm in length) was loaded into the tip of the catheter needle. The needle was quickly inserted through the skin toward the selected acupoint, followed by slow advancement. Upon the arrival of deqi, the stylet was gently advanced while simultaneously withdrawing the needle sleeve, leaving the catgut suture embedded within the acupoint. The needle insertion depth was maintained between 1.5 and 2 cm. After needle removal, a disinfected cotton ball was applied to press the puncture site to prevent bleeding. | once ten days |
| Lu jin2014 | Group 1:BL18 (Ganshu), BL20 (Pishu), BL23 (Shenshu), GB34 (Yanglingquan), SP6 (Sanyinjiao), ST40 (Fenglong)  Group 2:CV12 (Zhongwan), CV10 (Xiawan), ST25 (Tianshu), ST29 (Guilai), CV4 (Guanyuan), EX-CA1 (Zigong), SP9 (Yinlingquan) | The two sets of acupoints were used alternately. The medical catgut sutures were embedded into the subcutaneous tissue at the locations of the aforementioned acupoints, following the standard procedure for minimally invasive acupoint catgut embedding. | once a week |
| Li Yu′e2024 | Main Acupoints: CV3 (Zhongji), ST36 (Zusanli), SP6 (Sanyinjiao), CV4 (Guanyuan), ST40 (Fenglong, bilateral), SP9 (Yinlingquan, bilateral)  Adjunctive Acupoints:ST25 (Tianshu), EX-CA1 (Zigong), ST24 (Huaroumen), SP15 (Daheng), EX-B3 (Yishu), CV6 (Qihai) | After the patient emptied the bladder and assumed a supine position, the selected acupoints were strictly disinfected. A penetrating needle technique was applied at CV4 towards CV6 . The remaining acupoints were perpendicularly needled. After insertion, a rotating and twirling manipulation with neutral reinforcement-reduction technique and moderate stimulation was applied until deqi was achieved. The needles were retained for 30 minutes, and the needle manipulation was repeated every 10 minutes. | once every two days |
| Gan Xiaoli  2022 | Main Acupoints:CV4 (Guanyuan), SP6 (Sanyinjiao), ST25 (Tianshu), ST40 (Fenglong), CV6 (Qihai), CV12 (Zhongwan), EX-CA1 (Zigong), ST21 (Liangmen)  Adjunctive Acupoints: BL23 (Shenshu), KI7 (Fuliu), CV2 (Qugu), BL32 (Ciliao), LR3 (Taichong), KI3 (Taixi), SP9 (Yinlingquan), selected based on syndrome differentiation | ACE:The absorbable suture was embedded into the acupoint using a catgut implantation needle. Medical adhesive tape was applied to cover the puncture site.  Mox:Governor Vessel Moxibustion: The patient was placed in a prone position.Du moxibustion powderwas evenly sprinkled over the area along the Governor Vessel. A layer of heated ginger pulp, approximately 2–3 cm thick and 10 cm wide, was spread over the powder. Moxa cones with a base diameter of about 2 cm were shaped from moxa wool and evenly placed on the corresponding acupoints of the Governor Vessel and the Bladder Meridian. The moxa cones were ignited and allowed to burn completely. This process was repeated twice, for a total of three consecutive rounds of moxibustion. After the final round, the ginger pulp was removed and the area was disinfected. Conception Vessel Moxibustion:The patient was placed in a supine position. The same method as described above was applied along the Conception Vessel. | Mox: once a week  ACE: once ten days |
| Chen Danshan  2019 | CV12 (Zhongwan), CV4 (Guanyuan), CV3 (Zhongji), ST25 (Tianshu), ST29 (Guilai), ST36 (Zusanli), SP6 (Sanyinjiao), BL20 (Pishu), BL23 (Shenshu), BL32 (Ciliao) | Mox：A 10 cm Thunder-Fire moxa stick was ignited and placed inside a specialized moxibustion box. A towel was spread over the patient's abdomen and lower limbs. The moxibustion box was positioned over CV12，CV4,CV3, ST25, ST29 , and ST36. Another towel was placed on top of the box, with the burning end maintained approximately 5 cm from the skin. The procedure was alternated among SP6 , BL20 , BL23, and BL32 , with 15 minutes of moxibustion applied to each acupoint.  ACE：A 1.5–2 cm segment of PGLA suture was loaded into a No. 7 injection needle, with 0.5 cm of the suture exposed beyond the needle tip. For abdominal points, perpendicular insertion was applied; for back points, subcutaneous insertion was used; and for lower limb points, perpendicular needling was performed. The needle was inserted to a depth of 1.8–2.3 cm. The suture was then embedded into the muscular layer of the acupoint by withdrawing the needle while simultaneously rotating the needle body. | Mox: Once five days;  ACE: once ten days |
| Pang Hongmei2021 | Group 1：CV8 (Shenque), DU16 (Fengfu), GB20 (Fengchi, bilateral)  Group 2：BL20 (Pishu, bilateral), BL32 (Ciliao, bilateral), SP6 (Sanyinjiao, bilateral), BL23 (Shenshu, bilateral) | A towel was placed over the patient's abdomen, back, or lower limbs. A 10-cm Thunder-Fire moxa stick was ignited and placed into a specialized large-hole moxibustion box. The box was first positioned over the patient's abdomen and both ears, targeting acupoints CV8, GV16, and GB20. Another towel was placed on top of the moxibustion box to ensure the burning end maintained a distance of approximately 5 cm from the skin. Each session lasted 15 minutes. Then, the treatment was alternated to the areas of BL20 (bilateral), BL32 (bilateral), SP6 (bilateral), and BL23 (bilateral), with each session also lasting 15 minutes. | once ten days |
| Sheng Wenzhen2021 | Main Acupoints:ST25 (Tianshu), SP15 (Daheng), GB26 (Daimai), BL23 (Shenshu), BL18 (Ganshu), CV3 (Zhongji), CV4 (Guanyuan)  Adjunctive Acupoints:ST36 (Zusanli), SP6 (Sanyinjiao), BL20 (Pishu), ST40 (Fenglong), ST37 (Shangjuxu) | The patient was placed in an appropriate position, and the local skin for suture embedding was disinfected with povidone-iodine. Using the catheter needle embedding technique, a 4-0 absorbable surgical suture of appropriate length was threaded into the needle tube, with the other half of the suture folded back toward the tube. The needle was then inserted into the acupoint, successfully implanting the double-stranded suture into the body. Medical adhesive tape was applied to cover the puncture site. | once twelve days |
| Li Ning2016 | ST25 (Tianshu), CV12 (Zhongwan), CV4 (Guanyuan), CV6 (Qihai), SP15 (Daheng), ST24 (Huaroumen), ST28 (Shuidao), SP16 (Fu‘ai), SP14 (Fujie), ST29 (Guilai) | 0.40 mm × 75 mm disposable acupuncture needles were inserted into the acupoints. After the arrival of deqi, the needles​were retained for 45 minutes. During this period, the needles were manipulated three times using the even reinforcing-reducing technique. Simultaneously, TDP lamp irradiation was applied to the abdominal area. | three times a week |
| Lu Ling2020 | CV10 (Xiawan), CV12 (Zhongwan), CV4 (Guanyuan), CV6 (Qihai), ST25 (Tianshu), ST21 (Liangmen, bilateral), ST28 (Shuidao, bilateral) | Following acupoint localization, filiform needles measuring 0.25 mm × 40 mm were swiftly inserted subcutaneously. The needles were then advanced slowly until tissue resistance was encountered, at which point needle insertion was halted. This technique was performed without employing additional needle manipulation methods such as lifting-thrusting or rotating. The needles were retained for a total of 30 minutes. | twice a week |
| Lai Maohua  2010 | CV12 (Zhongwan), CV10 (Xiawan), CV6 (Qihai), CV4 (Guanyuan), ST21 (Liangmen, bilateral), ST25 (Tianshu), ST28 (Shuidao) | Acupoints were punctured subcutaneously using 0.25 mm × 40 mm filiform needles with rapid insertion, followed by slow advancement to the deep layer. Needle insertion was halted when a slight resistance was felt, without applying additional manipulation techniques such as lifting, thrusting, or rotating. The needles were retained for 30 minutes. | twice a week |
| Wu Jia2020 | Main Acupoints:CV4 (Guanyuan), SP6 (Sanyinjiao), ST25 (Tianshu), ST40 (Fenglong), CV6 (Qihai), CV12 (Zhongwan), EX-CA1 (Zigong), ST21 (Liangmen) Adjunctive Acupoints :BL23 (Shenshu), KI7 (Fuliu), CV2 (Qugu), BL32 (Ciliao), LR3 (Taichong), KI3 (Taixi), SP9 (Yinlingquan)，selected based on syndrome differentiation | Absorbable sutures and an implantation needle were used. The sutures were immersed in alcohol for disinfection. Guided by the implantation needle, the suture was embedded into the acupoint. Medical adhesive tape was applied to cover the puncture site. | once ten days |
| Zhai Zhenyuan2017 | Group1:BL18 (Ganshu, bilateral), BL17 (Geshu, bilateral), CV3 (Zhongji), GB26 (Daimai, bilateral), CV4 (Guanyuan), ST36 (Zusanli, bilateral), SP6 (Sanyinjiao, bilateral)  Group2:BL23 (Shenshu, bilateral), BL20 (Pishu, bilateral), CV9 (Shuifen), EX-CA2 (Luanchao, bilateral), ST40 (Fenglong, bilateral), ST25 (Tianshu, bilateral), SP9 (Yinlingquan, bilateral) | The two treatment groups were alternated. A sterilized 3-0 gauge medical catgut suture (0.7 cm in length) was loaded into the tip of a catheter needle. The needle was swiftly inserted through the skin and then advanced slowly into the acupoint. Upon the arrival of deqi, both the stylet and needle sleeve were withdrawn, leaving the catgut suture embedded within the acupoint. After needle removal, routine pressure was applied to the puncture site for hemostasis, followed by disinfection. | once a week |
| Huang Chengyi  2024 | Main Acupoints:SP6 (Sanyinjiao, bilateral), ST40 (Fenglong, bilateral), ST25 (Tianshu, bilateral), CV3 (Zhongji), BL23 (Shenshu), CV12 (Zhongwan), CV4 (Guanyuan)  Adjunctive Acupoints：ST36 (Zusanli, bilateral), KI3 (Taixi, bilateral), EX-CA1 (Zigong), ST28 (Shuidao), ST29 (Guilai), GV4 (Mingmen), CV6 (Qihai)，selected based on menstrual cycle | The patient was placed in a supine position. The skin at the acupoints was routinely disinfected with 75% alcohol. Using a single-handed technique, a 0.30 mm × 40 mm filiform needle was rapidly inserted to a depth of approximately 0.5–1.2 cm. This was followed by lifting, thrusting, and rotating techniques until deqi was achieved. | three times a week |
| Fu Wenhui  2020 | Group 1:ST40 (Fenglong, bilateral), BL20 (Pishu, bilateral), ST36 (Zusanli, bilateral), BL23 (Shenshu, bilateral), SP6 (Sanyinjiao, bilateral), BL18 (Ganshu, bilateral), KI3 (Taixi, bilateral)  Group 2:ST25 (Tianshu, bilateral), EX-CA1 (Zigong, bilateral), CV4 (Guanyuan), SP10 (Xuehai, bilateral), ST36 (Zusanli, bilateral), ST40 (Fenglong, bilateral), SP6 (Sanyinjiao, bilateral), KI3 (Taixi, bilateral) | EA：For Group 1 acupoints: KI3 is perpendicularly inserted 0.5 cun, BL20 and BL18 are inserted obliquely downward 0.5 cun, BL23 is perpendicularly inserted 1 cun, ST36, ST40, and SP6 are perpendicularly inserted 1.5 cun. For Group 2 acupoints: CV4 and EX-CA1 are inserted obliquely downward 1 cun, SP10 and ST25 are perpendicularly inserted 1.5 cun. The needling methods for KI3, ST36, ST40, and SP6 are the same as in Group 1. After deqi is achieved, select the filiform needles at BL23, ST40, and SP6 from Group 1 or ST25, ST40, and SP6 from Group 2 to connect to the electroacupuncture device. Use a continuous wave at 2 Hz and treat for 30 minutes.  Moving cupping：Moving cupping was applied along the Belt Vessel (Daimai) and Governor Vessel (Du Mai). Vaseline was applied to the patient's back or abdomen. A glass fire cup with an inner diameter of 4.0 cm was used. An ignited alcohol cotton ball was swirled inside the cup for 2-3 revolutions, after which the cup was quickly placed on the skin to create suction. Holding the base of the cup with one hand, the practitioner moved it back and forth until the skin became erythematous. | once a day |
| Cai Xianbing2016 | EA:Main Acupoints:CV12 (Zhongwan), ST21 (Liangmen, bilateral), ST25 (Tianshu, bilateral), GB26 (Daimai, bilateral), CV6 (Qihai), CV4 (Guanyuan), ST28 (Shuidao, bilateral), SP10 (Xuehai, bilateral), ST34 (Liangqiu, bilateral), ST36 (Zusanli, bilateral), ST37 (Shangjuxu, bilateral), SP6 (Sanyinjiao, bilateral);Adjunctive Acupoints:LI4 (Hegu, bilateral), LR3 (Taichong, bilateral), SP9 (Yinlingquan, bilateral), ST40 (Fenglong, bilateral), LI11 (Quchi, bilateral), ST44 (Neiting, bilateral),selected based on syndrome differentiation  ACE:Main Acupoints:CV12 (Zhongwan), ST21 (Liangmen, bilateral), ST25 (Tianshu, bilateral), GB26 (Daimai, bilateral), CV4 (Guanyuan), ST28 (Shuidao, bilateral), SP10 (Xuehai, bilateral), ST36 (Zusanli, bilateral), SP6 (Sanyinjiao, bilateral), BL25 (Dachangshu, bilateral), DU14 (Dazhui);Adjunctive Acupoints:TE6 (Zhigou, bilateral), ST37 (Shangjuxu, bilateral), BL57 (Chengshan, bilateral), BL20 (Pishu, bilateral), SP9 (Yinlingquan, bilateral), ST40 (Fenglong, bilateral), LI11 (Quchi, bilateral), ST34 (Liangqiu, bilateral), BL23 (Shenshu, bilateral), BL18 (Ganshu, bilateral),selected based on syndrome differentiation | EA:After acupoint localization, needles were inserted rapidly. Upon the arrival of deqi, the reducing method was applied for excess patterns, while the reinforcing method was applied for deficiency patterns. Following the reinforcement-reduction manipulation, electroacupuncture leads were connected to five pairs of acupoints: CV12 , CV4 , ST25 (bilateral), GB26 (bilateral), ST36 , and SP6 . A continuous wave was used, with the intensity set to the maximum tolerance level of the patient. The needles were retained for 30 minutes before being withdrawn.  ACE: After strict disinfection of the selected acupoint areas, a medical catgut suture was loaded into a disposable sterile embedding needle using sterile forceps. The needle was quickly inserted through the skin toward the selected acupoint to a depth of approximately 1.5 cm. Upon the arrival of deqi, the stylet was advanced to push the catgut suture into the acupoint, leaving it embedded between the subcutaneous tissue and muscle layer. After needle withdrawal, the puncture site was pressed with a sterile dry cotton ball for a moment to prevent bleeding, followed by application of a sterile medical adhesive tape to protect the area. | EA: three times a week;  ACE: once fifteen days |
| Zhang Yaqin  2017 | CV3 (Zhongji), CV4 (Guanyuan), EX-CA1 (Zigong), SP6 (Sanyinjiao), KI7 (Fuliu), ST36 (Zusanli), KI13 (Qixue), SP10 (Xuehai), GV7 (Zhongshu) | The needles were inserted perpendicularly and retained for 30 minutes. | once a day |
| Wu Dan2020 | CV10 (Xiawan), CV12 (Zhongwan), CV4 (Guanyuan), CV6 (Qihai), ST25 (Tianshu), ST28 (Shuidao), ST21 (Liangmen, bilateral) | After routine disinfection, a disposable acupuncture needle (0.25 mm × 40 mm) was swiftly inserted perpendicularly into the subcutaneous tissue to a depth of 0.5–1.0 cun. No supplementary techniques, such as the neutral reinforcement-reduction method, lifting-thrusting, or rotating, were applied. The needle was slowly withdrawn when a slight resistance was felt and was then retained for 30 minutes. | twice a week |
| Li Juan2019 | CV3 (Zhongji), CV6 (Qihai), ST29 (Guilai), SP6 (Sanyinjiao), SP9 (Yinlingquan), LI4 (Hegu, bilateral), GV20 (Baihui) | The skin was tightened by pressing the surrounding area, and the needles were inserted gently. After all filiform needles were in place, manual stimulation was applied to elicit deqi. | once a day |
| Tang Liangying2015 | CV3 (Zhongji), CV4 (Guanyuan), CV12 (Zhongwan), ST36 (Zusanli), ST25 (Tianshu), SP15 (Daheng), ST40 (Fenglong), ST29 (Guilai), BL25 (Dachangshu), ST28 (Shuidao), BL21 (Weishu), BL20 (Pishu) | After routine local disinfection, a 3-0 medical catgut suture (1.5 cm in length) was loaded into the lumen of a disposable catheter needle. The needle was quickly inserted through the skin toward the acupoint and then advanced slowly. Upon the arrival of deqi, the stylet was gently advanced while simultaneously withdrawing the needle sleeve, leaving the catgut suture embedded within the acupoint. | once 15 days |
| Zhang Tong2013 | CV12 (Zhongwan), ST25 (Tianshu, bilateral), SP15 (Daheng, bilateral), GB26 (Daimai, bilateral), CV6 (Qihai), CV4 (Guanyuan), ST28 (Shuidao, bilateral), EX-CA1 (Zigong, bilateral), ST36 (Zusanli, bilateral), SP9 (Yinlingquan, bilateral), ST40 (Fenglong, bilateral), KI3 (Taixi, bilateral) | ACE：The patient was placed in a supine position, and routine disinfection was performed on the selected acupoints. A catgut suture was fully loaded into the tip of the catheter needle. The needle was quickly inserted through the skin toward the acupoint, then advanced slowly. Upon the arrival of deqi, the stylet was gently advanced while simultaneously withdrawing the needle sleeve, leaving the catgut suture embedded within the acupoint.  EA：After rapid needle insertion, the reducing method was applied for excess syndrome patterns, while the reinforcing method was applied for deficiency syndrome patterns upon the arrival of deqi. Electroacupuncture leads were connected to the ipsilateral ST25 (negative electrode) and GB26 (positive electrode). A dense-disperse wave was used, with the intensity set to the maximum tolerance level of the patient. The needles were retained for 30 minutes before withdrawal. | ACE: once a week;  EA: five times a week |
| Yin Guochao  2021 | CV12 (Zhongwan), CV6 (Qihai), EX-CA1 (Zigong), SP6 (Sanyinjiao), SP10 (Xuehai), ST36 (Zusanli), KI3 (Taixi), KI6 (Zhaohai), ST28 (Shuidao), CV3 (Zhongji), CV4 (Guanyuan), KI12 (Dahe), LR13 (Zhangmen), LR14 (Qimen), GB26 (Daimai), ST25 (Tianshu), SP15 (Daheng), ST40 (Fenglong), LI11 (Quchi), ST37 (Shangjuxu) | The appropriate needling technique was selected based on clinical pattern differentiation, with needle retention time ranging from 40 to 50 minutes per session. | 2~3times a week |
| Yang Xing2023 | ACE：ST25 (Tianshu), CV9 (Shuifen), ST26 (Wailing), CV7 (Yinjiao), ST24 (Huaroumen)  AAp：CO18 (Neifenmi), CO12 (Gan), CO10 (Shen), TF4 (Shenmen), TF2 (Zigong) | ACE: After disinfection, a disposable catgut-embedding needle was used for insertion. Upon elicitation of the needling sensation, the stylet was advanced while simultaneously withdrawing the needle sleeve, pushing the suture into the acupoint. During needle insertion, the depth and angle were tailored to the patient's specific condition.  AAp：Decorticated Wangbuliuxing seeds were applied to the selected auricular points. The points were then kneaded and pressed until the patient experienced soreness, numbness, or distension. The procedure was performed on alternating ears, with each point stimulated for one minute. | once two weeks |
| Li Yuchang  2020 | Group 1:TF2 (Zigong), AT3 (Luanchao), CO18 (Neifenmi), CO10 (Shen), CO13 (Pi)  Group 2:CO18 (Neifenmi), CO17 (Sanjiao), CO13 (Pi), CO7 (Dachang), TG2 (Jidian) | The treatment was alternated between the two groups. Selected Wangbuliuxing seeds were used for auricular point therapy. After affixing the seeds to the corresponding auricular points with medical adhesive tape, auricular point pressing was performed. Each session was conducted on a single ear, with the ears alternated between sessions. Each point was pressed 50 times per session. | 3times a day |
| Li Linan  2015 | EA：CV4 (Guanyuan), CV3 (Zhongji), SP6 (Sanyinjiao), EX-CA1 (Zigong)  AAp:TF2 (Zigong), AT3 (Luanchao), AT1 (Xiaqiumao), AT2 (Naochuiti), CO10 (Shen), CO18 (Neifenmi) | EA: Electrical stimulation was delivered by a G6805 electroacupuncture device. The parameters were set as follows: frequency of 3–4 Hz, intensity of 2–3 mA, and an intermittent pulse wave. The intensity was adjusted to the patient's tolerance level.  AAp: Wangbuliuxing seeds were placed on 0.5 cm × 0.5 cm adhesive tape and applied to the corresponding auricular points. The pressure was applied until the patient experienced sensations of heat, soreness, numbness, or distension. | once a week |
| Chen Jiaxin  2019 | EA:Main Acupoints:CV12 (Zhongwan), ST25 (Tianshu), CV6 (Qihai), EX-CA1 (Luanchao), SP6 (Sanyinjiao), PC6 (Neiguan);Adjunctive Acupoints:ST40 (Fenglong), LR3 (Taichong), SP10 (Xuehai),selected based on syndrome differentiation  ACE:Main Acupoints:CV12(Zhongwan), ST25 (Tianshu), CV6(Qihai),SP6(Sanyinjiao),BL23(Shenshu);Adjunctive Acupoints:ST40(Fenglong),BL18(Ganshu),BL17 (Geshu), selected based on syndrome differentiation | EA:All acupoints were routinely needled. Upon the arrival of deqi, an even reinforcing-reducing manipulation was applied. The needles were retained for 30 minutes. Electroacupuncture was then applied specifically to EX-CA1 and SP6, with a stimulation frequency of approximately 3 Hz. The intensity was set to a moderate level, ensuring it was tolerable for the patient and avoiding excessive stimulation.  ACE:A 1 cm segment of absorbable surgical suture was loaded into the tip of a disposable No. 8 injection needle. The needle was quickly inserted through the skin toward the selected acupoint and then advanced slowly. After reaching the required depth, the stylet was pushed while simultaneously withdrawing the needle sleeve, embedding the suture into the muscular layer or subcutaneous tissue at the acupoint. | EA: three times a week;  ACE: once two weeks |

# S4. Risk of bias summary

| **Study** | **Random sequence generation (selection bias)** | **Allocation concealment (selection bias)** | **Blinding of participants and personnel (performance bias)** | **Blinding of outcome assessment (detection bias)** | **Incomplete outcome data (attrition bias)** | **Selective reporting (reporting bias)** | **Other bias** |
| --- | --- | --- | --- | --- | --- | --- | --- |
| Yan-Hua Zheng 2013 | Low risk | Low risk | Unclear risk | Unclear risk | Low risk | Low risk | Unclear risk |
| Jie Cao2023 | Low risk | Unclear risk | Low risk | Unclear risk | Unclear risk | Low risk | Unclear risk |
| Raden Muharam2022 | Low risk | Low risk | Low risk | Low risk | Low risk | Low risk | Unclear risk |
| Tianyu Wu2024 | Low risk | Low risk | Unclear risk | Low risk | Low risk | Low risk | Unclear risk |
| Yue Jin2020 | Low risk | Unclear risk | Unclear risk | Unclear risk | Low risk | Low risk | Unclear risk |
| Yue Jin2021 | Low risk | Low risk | Unclear risk | Unclear risk | Low risk | Low risk | Unclear risk |
| Zhang Zeli 2016 | Low risk | Unclear risk | Unclear risk | Unclear risk | Low risk | Low risk | Unclear risk |
| Yu Xiaoli2023 | Low risk | Unclear risk | Unclear risk | Unclear risk | Low risk | Low risk | Unclear risk |
| Wang Jiali 2009 | Low risk | Unclear risk | Unclear risk | Unclear risk | Low risk | Low risk | Unclear risk |
| He Danjuan  2020 | Low risk | Low risk | Unclear risk | Unclear risk | Low risk | Low risk | Unclear risk |
| Yan Huili2023 | Low risk | Unclear risk | Unclear risk | Unclear risk | Low risk | Low risk | Unclear risk |
| Su Ting2025 | Low risk | Low risk | Unclear risk | Unclear risk | Low risk | Low risk | Unclear risk |
| Wang Limei  2025 | Low risk | Low risk | Unclear risk | Unclear risk | Low risk | Low risk | Unclear risk |
| Ma Guizhi  2020 | Low risk | Unclear risk | Unclear risk | Unclear risk | Low risk | Low risk | Unclear risk |
| He Danjuan  2020 | Low risk | Low risk | Unclear risk | Unclear risk | Low risk | Low risk | Unclear risk |
| Zhang Jianfeng  2016 | Low risk | Unclear risk | Unclear risk | Unclear risk | Low risk | Low risk | Unclear risk |
| Chen Li2018 | Low risk | Unclear risk | Unclear risk | Unclear risk | Low risk | Low risk | Unclear risk |
| Lu jin2014 | Unclear risk | Unclear risk | Unclear risk | Unclear risk | Low risk | Low risk | Unclear risk |
| Li Yu′e2024 | Low risk | Unclear risk | Unclear risk | Unclear risk | Low risk | Low risk | Unclear risk |
| Gan Xiaoli  2022 | Low risk | Unclear risk | Unclear risk | Unclear risk | Low risk | Low risk | Unclear risk |
| Chen Danshan  2019 | Low risk | Unclear risk | Unclear risk | Unclear risk | Low risk | Low risk | Unclear risk |
| Pang Hongmei2021 | Low risk | Unclear risk | Unclear risk | Unclear risk | Low risk | Low risk | Unclear risk |
| Sheng Wenzhen2021 | Low risk | Unclear risk | Unclear risk | Unclear risk | Low risk | Low risk | Unclear risk |
| Li Ning2016 | Low risk | Unclear risk | Unclear risk | Unclear risk | Low risk | Low risk | Unclear risk |
| Lu Ling2020 | High risk | High risk | Unclear risk | Unclear risk | Low risk | Low risk | Unclear risk |
| Lai Maohua  2010 | Low risk | Unclear risk | Unclear risk | Unclear risk | Low risk | Low risk | Unclear risk |
| Wu Jia2020 | Low risk | Unclear risk | Unclear risk | Unclear risk | Low risk | Low risk | Unclear risk |
| Zhai Zhenyuan2017 | Low risk | Unclear risk | Unclear risk | Unclear risk | Low risk | Low risk | Unclear risk |
| Huang Chengyi  2024 | Low risk | Unclear risk | Unclear risk | Unclear risk | Low risk | Low risk | Unclear risk |
| Fu Wenhui  2020 | Low risk | Unclear risk | Unclear risk | Unclear risk | Low risk | Low risk | Unclear risk |
| Cai Xianbing2016 | Low risk | Unclear risk | Unclear risk | Unclear risk | Low risk | Low risk | Unclear risk |
| Zhang Yaqin  2017 | Low risk | Unclear risk | Unclear risk | Unclear risk | Low risk | Low risk | Unclear risk |
| Wu Dan2020 | Low risk | Unclear risk | Unclear risk | Unclear risk | Low risk | Low risk | Unclear risk |
| Li Juan2019 | Low risk | Unclear risk | Low risk | Unclear risk | Low risk | Low risk | Unclear risk |
| Tang Liangying2015 | Low risk | Unclear risk | Unclear risk | Unclear risk | Low risk | Low risk | Unclear risk |
| Zhang Tong2013 | Low risk | Unclear risk | Unclear risk | Unclear risk | Low risk | Low risk | Unclear risk |
| Yin Guochao  2021 | Low risk | Unclear risk | Unclear risk | Unclear risk | Low risk | Low risk | Unclear risk |
| Yang Xing2023 | High risk | High risk | Unclear risk | Unclear risk | Low risk | Low risk | Unclear risk |
| Li Yuchang  2020 | Low risk | Unclear risk | Unclear risk | Unclear risk | Low risk | Low risk | Unclear risk |
| Li Linan  2015 | Low risk | Unclear risk | Unclear risk | Unclear risk | Low risk | Low risk | Unclear risk |
| Chen Jiaxin  2019 | Low risk | Unclear risk | Unclear risk | Unclear risk | Low risk | Low risk | Unclear risk |

# S5. Network meta-analysis of BMI outcomes

S5.1. Comparisons of consistency model and inconsistency model

|  | **τ^2^** | **I^2^** | **DIC** | **‾D_res_^*^** |
| --- | --- | --- | --- | --- |
| Consistency model | 2.57 | 0.6% | 146.44 | 75.44 |
| Inconsistency model | 3.04 | 0.4% | 147.32 | 75.34 |

*Compared with 76 data points.

S5.2. Heterogeneity

| **Median** $\boldsymbol{\tau}$ **(95%CI)** | **Median τ^2^ (95%CI)** | **MCID** |
| --- | --- | --- |
| 1.59(1.20,2.16) | 2.52(1.44,4.66) | 1.69 |

MICD: Pooling all baseline BMI standard deviations included in the studies yielded an overall pooled standard deviation of 3.37 kg/m². Based on the distribution-based approach, the MCID threshold was defined as 0.5 × SD = 1.69 kg/m².


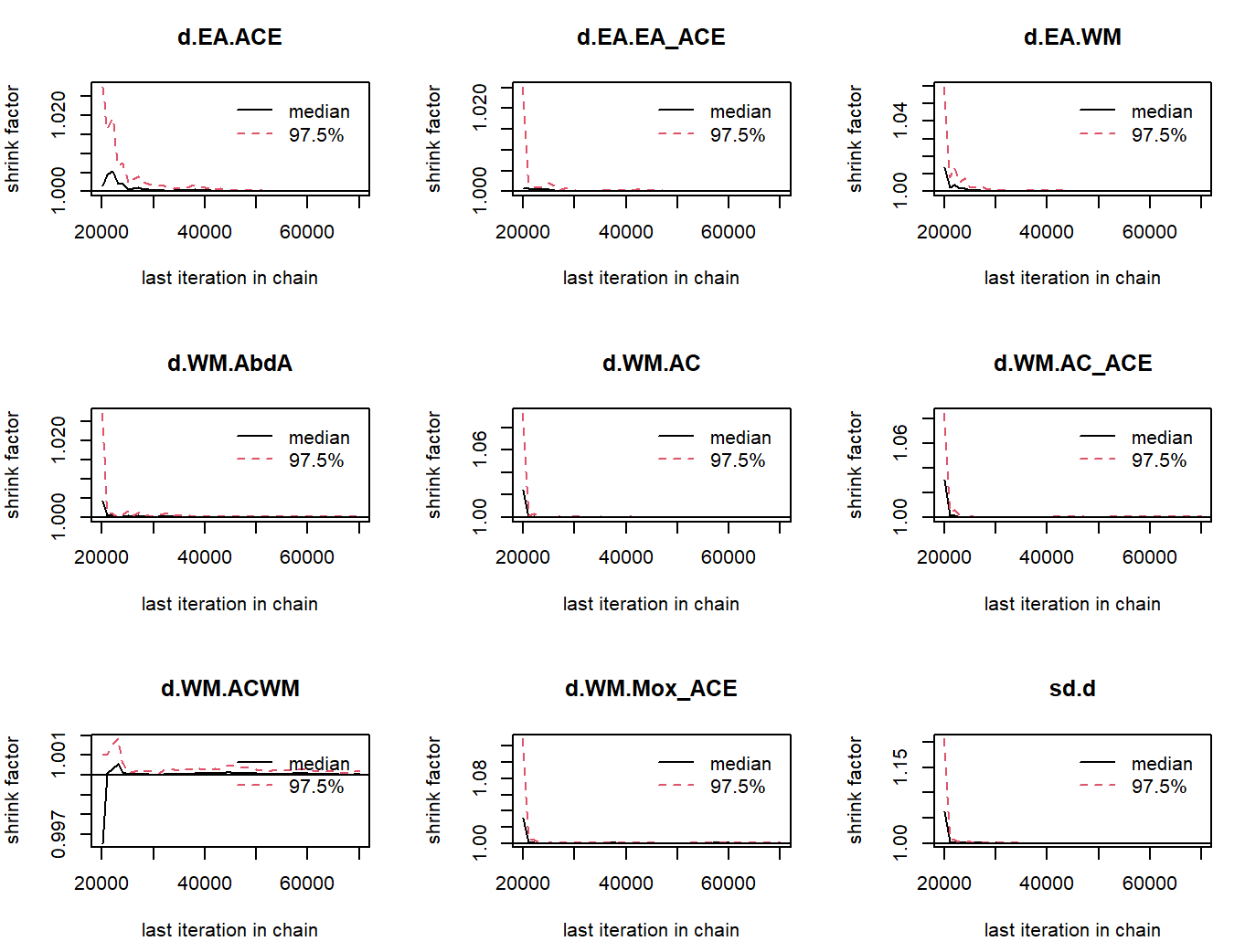
S5.3. Convergence assessment using the potential scale reduction factors

S5.4. Node-splitting results using Bayesian approach


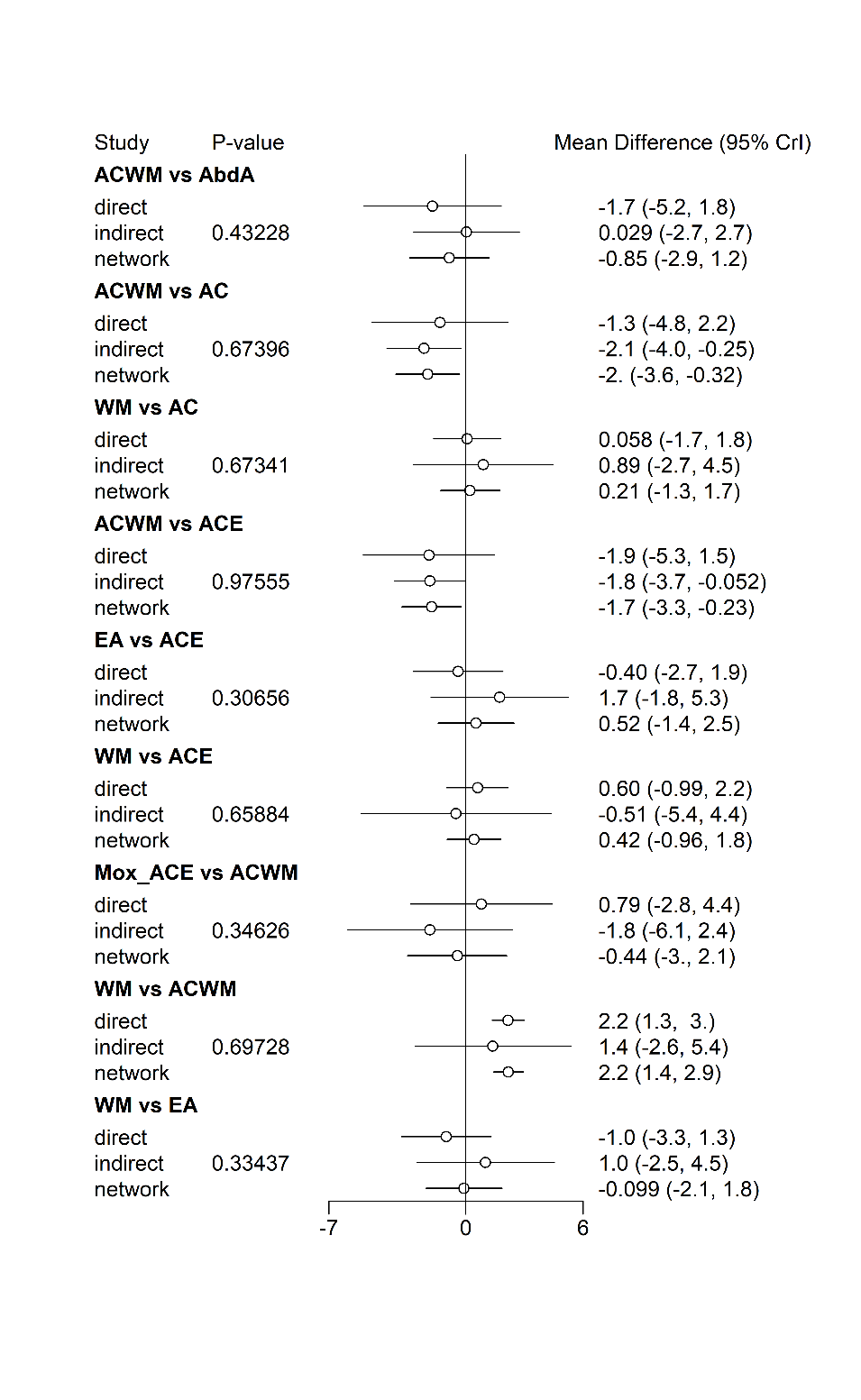


**Mox_ACE**,Moxibustion plus Acupoint Catgut Embedding; **ACWM,** Acupuncture Combined with Medication; **AbdA,** Abdominal Acupuncture; **ACE,** Acupoint Catgut Embedding; **AC,** Acupuncture; **EA,** Electroacupuncture; **WM,** Western Medicine.

S5.5.1. Sensitivity analysis treating each acupuncture-medication combination (ACWM) as an independent node

**AC_Letrozole,** Acupuncture plus Letrozole; **Mox_ACE_COC_M,** Moxibustion plus Acupoint Catgut Embedding plus Combined Oral Contraceptive plus Metformin; **Mox_ACE**,Moxibustion plus Acupoint Catgut Embedding; **ACE_COC_M,** Acupoint Catgut Embedding plus Combined Oral Contraceptive plus Metformin; **Orlistat_ACE,** Orlistat plus Acupoint Catgut Embedding; **ACE_COC,** Acupoint Catgut Embedding plus Combined Oral Contraceptive; **Mox_M,** Moxibustion plus Metformin; **AC_M,** Acupuncture plus Metformin; **ACE_M,** Acupoint Catgut Embedding plus Metformin; **AC_ACE,** Acupuncture plus Acupoint Catgut Embedding; **EA_Mox_M,** Electroacupuncture plus Moxibustion plus Metformin; **AC_acarbose,** Acupuncture plus **acarbose; AbdA_M,** Abdominal Acupuncture plus Metformin; **EA_ACE,** Electroacupuncture plus Acupoint Catgut Embedding; **AbdA,** Abdominal Acupuncture; **AAp_M,** Acupoint Catgut Embedding plus Metformin; **ACE,** Acupoint Catgut Embedding; **EA_M,**Electroacupuncture plus Metformin; **AC,** Manual Acupuncture; **EA,** Electroacupuncture; **WM,** Western Medicine.

S5.5.2. League table after excluding the study based on BMI ≥23 kg/m² cut-off

**Mox_ACE**, Moxibustion plus Acupoint Catgut Embedding; **ACWM,** Acupuncture Combined with Medication; **AC_ACE,** Acupuncture plus Acupoint Catgut Embedding; **AbdA,** Abdominal Acupuncture; **EA_ACE,** Electroacupuncture plus Acupoint Catgut Embedding; **ACE,** Acupoint Catgut Embedding; **AC,** Manual Acupuncture; **EA,** Electroacupuncture; **WM,** Western Medicine.

S5.6. funnel plot

| **Covariates** | **B (95%CI)** | **τ^2^** | **I^2^** | **DIC** | **‾D_res_^*^** |
| --- | --- | --- | --- | --- | --- |
| Publication year | -0.69(-2.34, 0.95) | 2.62 | 0.2% | 146.16 | 75.13 |
| Age (years) | 0.04 (-1.16, 1.28) | 2.69 | 0.4% | 146.39 | 75.28 |
| Sample size | -0.89 (-2.13, 0.41) | 2.50 | 0.2% | 146.10 | 75.15 |
| Acupuncture frequency (frequency/week) | 0.97 (-0.15, 2.07) | 2.37 | 0.6% | 146.26 | 75.47 |
| Period of treatment (month) | -1.39 (-2.87, 0.05) | 2.31 | 0.5% | 146.08 | 75.35 |


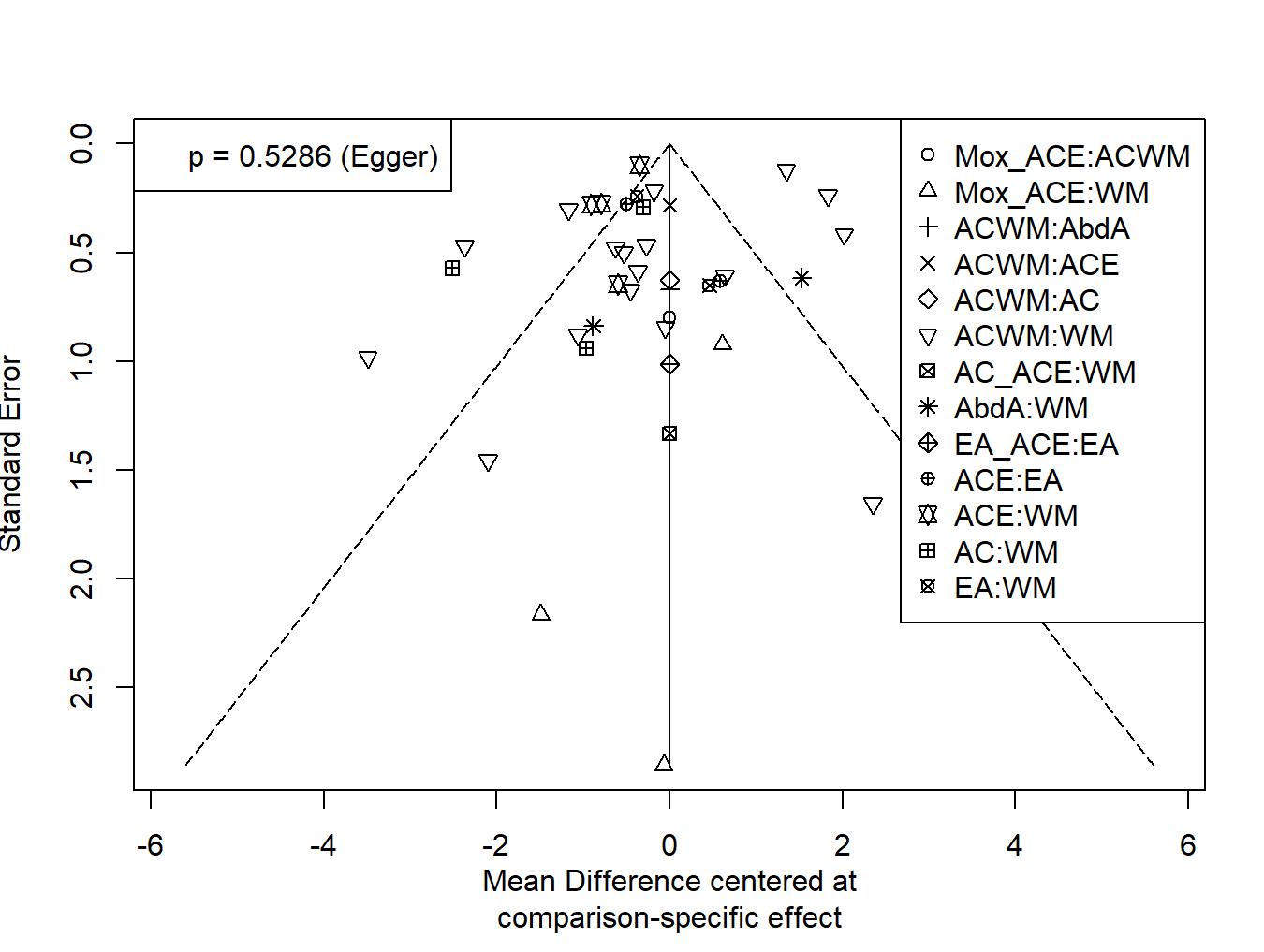


S5.7. Results for meta-regression

*Compared with 76 data points.

S5.8. Confidence assessment


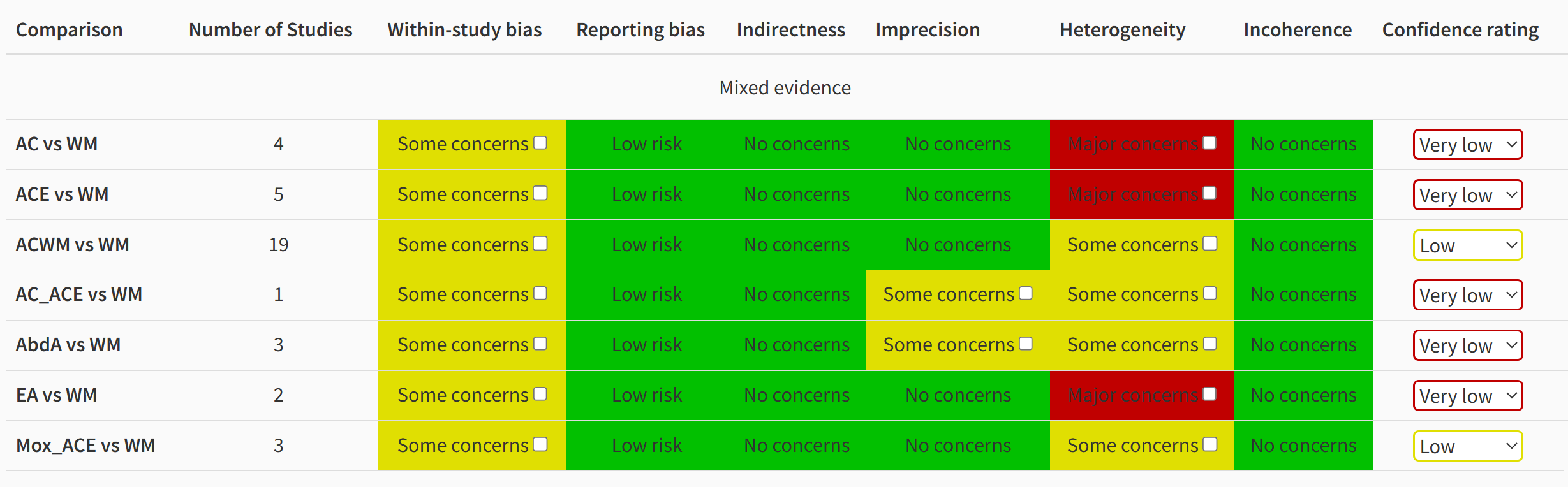


# S6. Network meta-analysis of HOMA-IR outcomes

S6.1. Comparisons of consistency model and inconsistency model

|  | **τ^2^** | **I^2^** | **DIC** | **‾D_res_^*^** |
| --- | --- | --- | --- | --- |
| Consistency model | 0.25 | 1% | 83.31 | 43.52 |
| Inconsistency model | 0.34 | 1% | 84.65 | 43.51 |

*Compared with 44 data points.

S6.2. Heterogeneity

| **Median** $\boldsymbol{\tau}$ **(95%CI)** | **Median τ^2^ (95%CI)** | **MCID** |
| --- | --- | --- |
| 0.48(0.27,0.85) | 0.23(0.07,0.72) | 0.79 |

MICD: Pooling all baseline HOMA-IR standard deviations included in the studies yielded an overall pooled standard deviation of 1.57. Based on the distribution-based approach, the MCID threshold was defined as 0.5 × SD = 0.79.

S6.3. Convergence assessment using the potential scale reduction factors


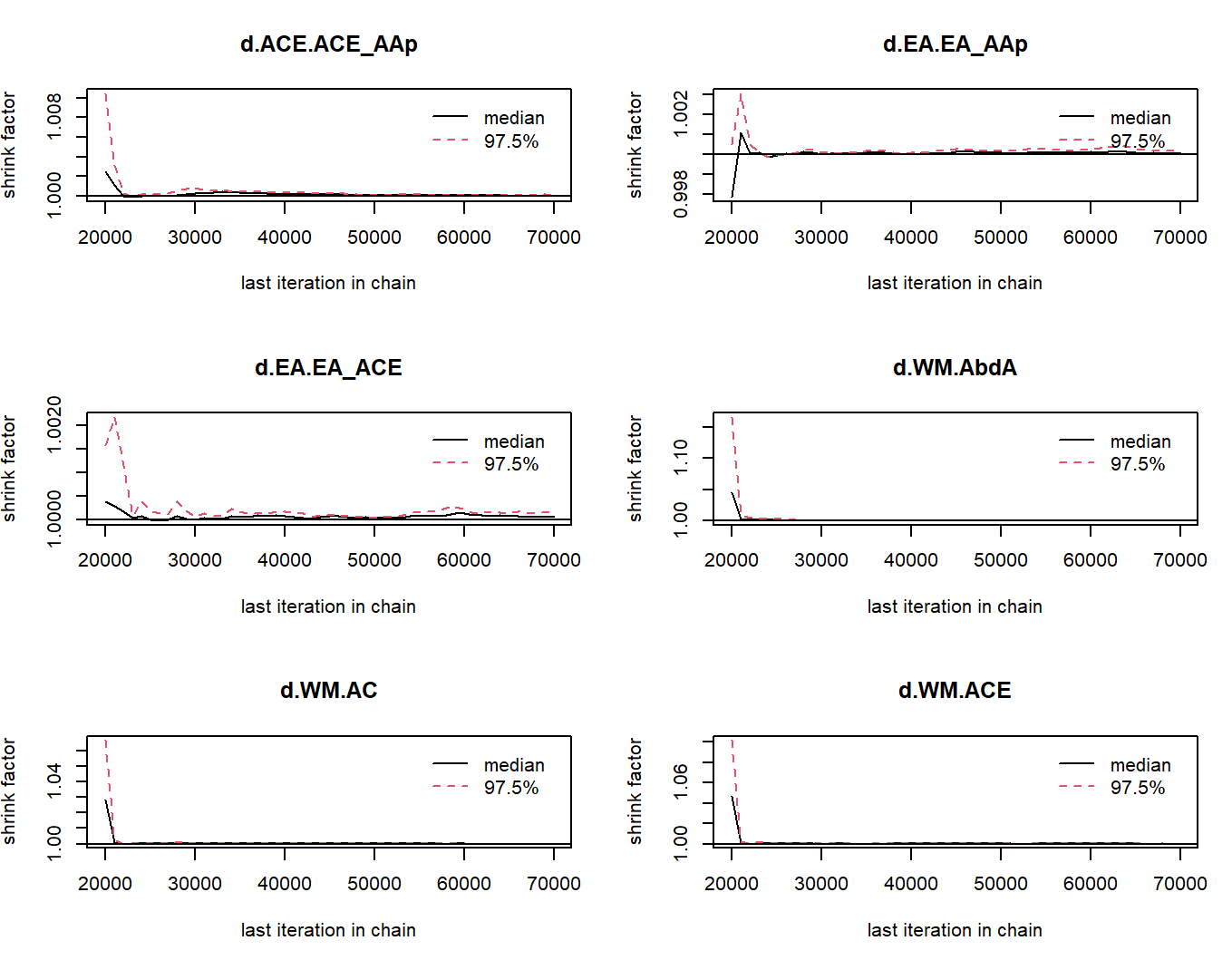

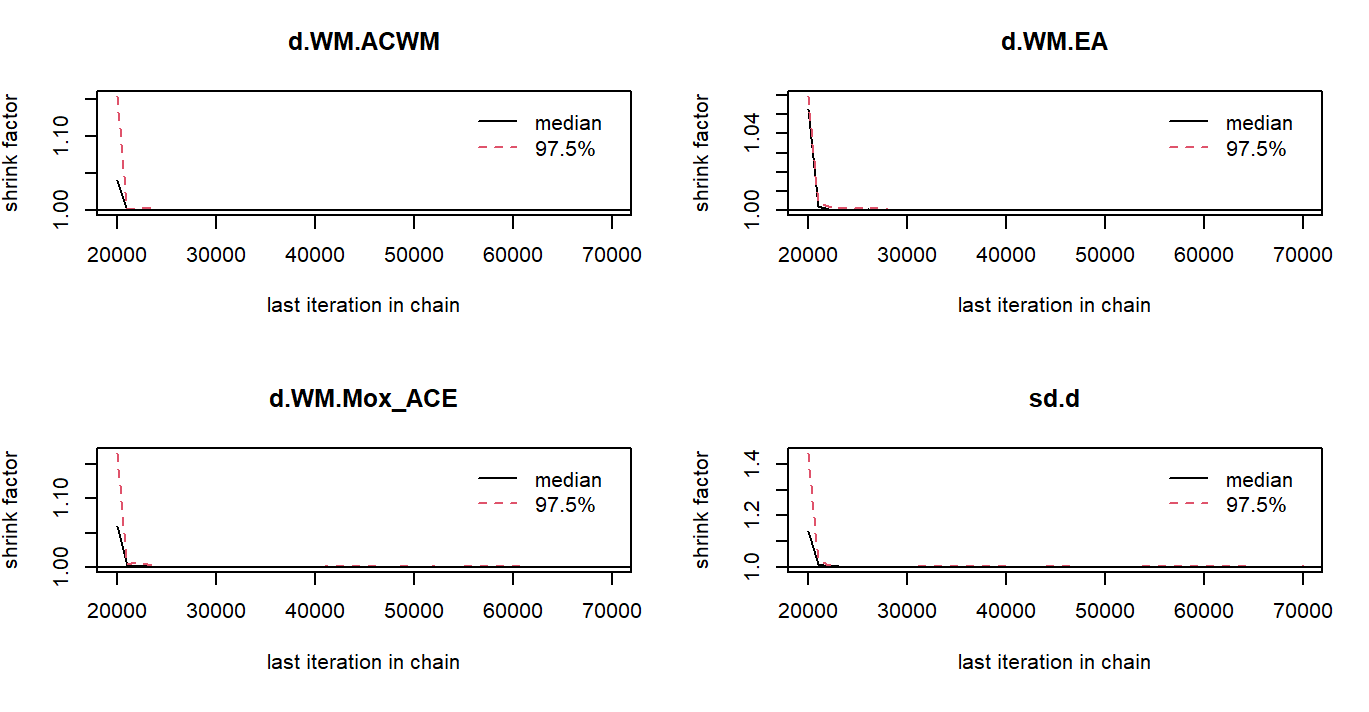


S6.4. Node-splitting results using Bayesian approach


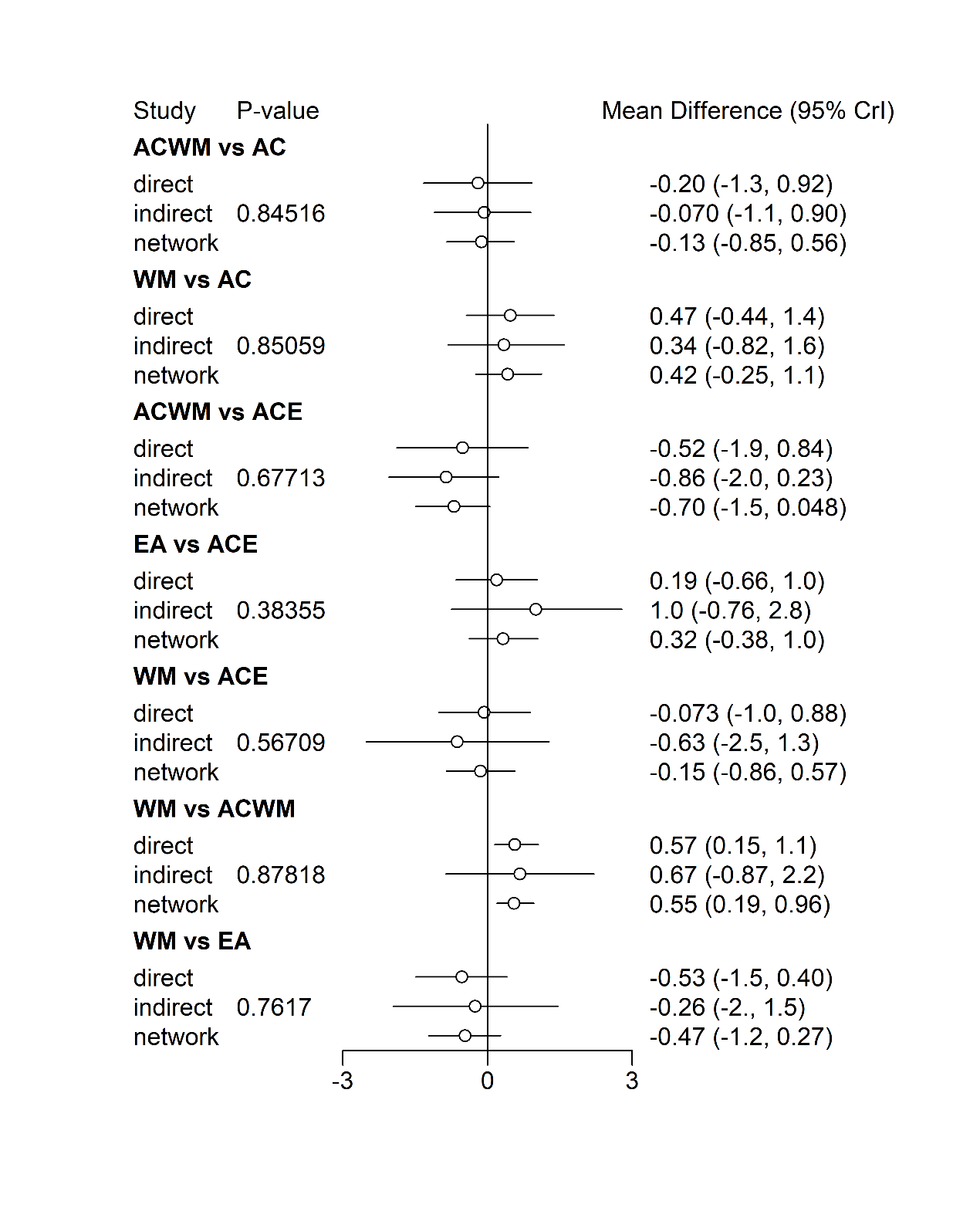


**ACWM,** Acupuncture Combined with Medication; **AC,** Acupuncture; **WM,** Western Medicine; **ACE,** Acupoint Catgut Embedding; **EA,** Electroacupuncture.

S6.5.1. Sensitivity analysis treating each acupuncture-medication combination (ACWM) as an independent node

**Mox_M,** Moxibustion plus Metformin; **AC_CC,** Acupuncture plus Clomiphene Citrate; **Mox_ACE**, Moxibustion plus Acupoint Catgut Embedding; **EA_M,** Electroacupuncture plus Metformin; **AC_acarbose,** Acupuncture plus acarbose; **ACE_M,** Acupoint Catgut Embedding plus Metformin; **AC,** Manual Acupuncture; **AAp_M,** Auricular Acupressure plus Metformin; **AbdA,** Abdominal Acupuncture; **ACE_AAp,** Acupoint Catgut Embedding plus Auricular Acupressure; **WM,** Western Medicine; **EA_ACE,** Electroacupuncture plus Acupoint Catgut Embedding; **ACE,** Acupoint Catgut Embedding; **EA_AAp,** Electroacupuncture plus Auricular Acupressure**; AC_M,** Acupuncture plus Metformin; **EA,** Electroacupuncture.

S6.5.2. League table after excluding the study based on BMI ≥23 kg/m² cut-off

**Mox_ACE**, Moxibustion plus Acupoint Catgut Embedding; **ACWM,** Acupuncture Combined with Medication; **AC,** Manual Acupuncture; **AbdA,** Abdominal Acupuncture; **ACE_AAp,** Acupoint Catgut Embedding plus Auricular Acupressure; **WM,** Western Medicine; **EA_ACE,** Electroacupuncture plus Acupoint Catgut Embedding; **ACE,** Acupoint Catgut Embedding; **EA_AAp,** Electroacupuncture plus Auricular Acupressure; **EA,** Electroacupuncture.

S6.6. funnel plot

| **Covariates** | **B (95%CI)** | **τ^2^** | **I^2^** | **DIC** | **‾D_res_^*^** |
| --- | --- | --- | --- | --- | --- |
| Publication year | -0.46( -1.18, 0.31) | 0.21 | 3% | 84.07 | 44.23 |
| Age (years) | -0.03 (-0.83, 0.77) | 0.27 | 1% | 83.86 | 43.59 |
| Sample size | 0.27 (-0.38, 1.01) | 0.26 | 0% | 83.04 | 43.00 |
| Acupuncture frequency (frequency/week) | 0.32 ( -0.17, 0.88) | 0.23 | 0.3% | 82.76 | 43.14 |
| Period of treatment (month) | -0.07 (-1.06, 0.94) | 0.27 | 1% | 83.99 | 43.64 |
| Baseline HOMA-IR value | -0.24 (-0.99, 0.45) | 0.27 | 0.0% | 83.73 | 43.40 |


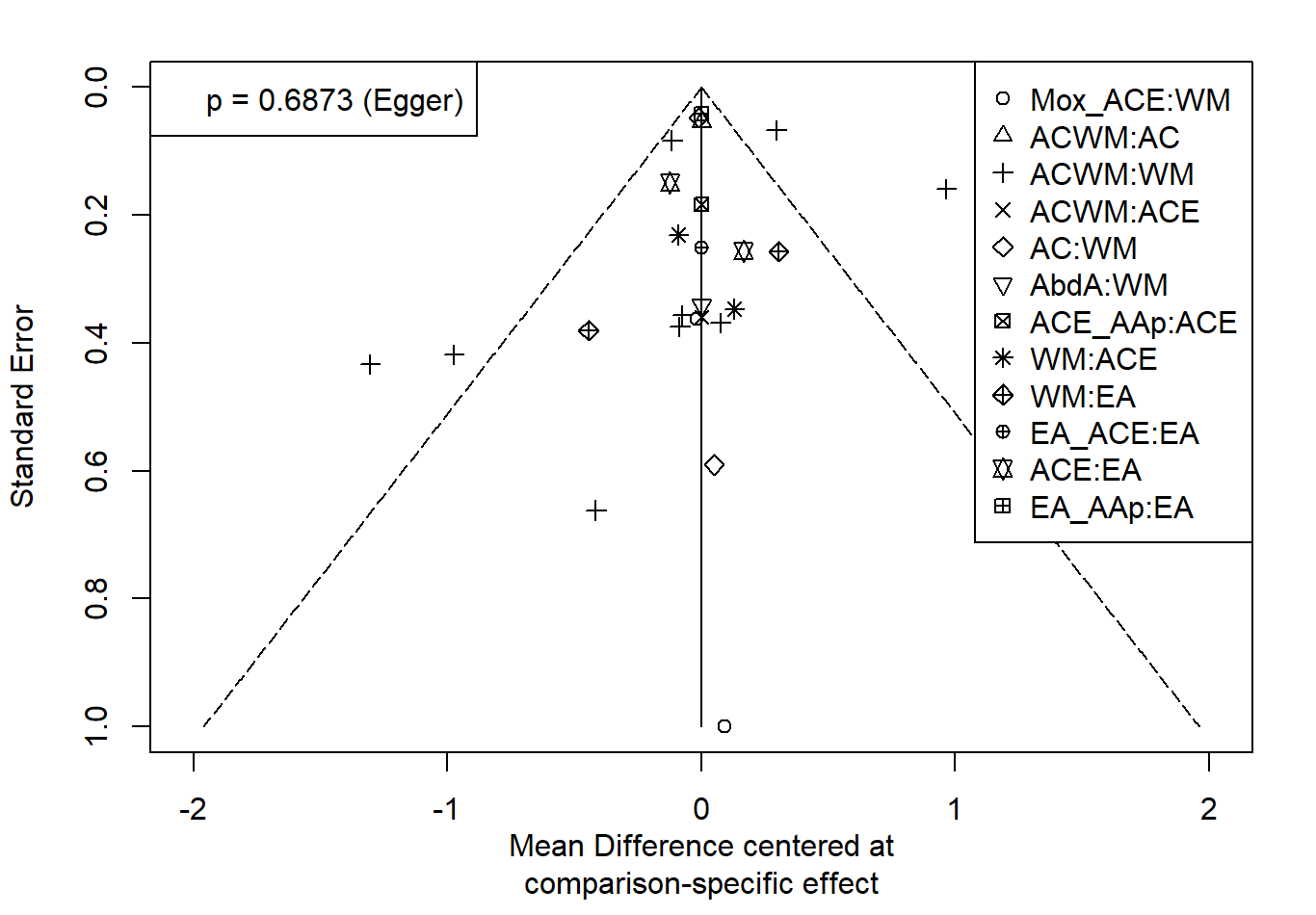


S6.7. Results for meta-regression

*Compared with 44 data points.

S6.8. Confidence assessment


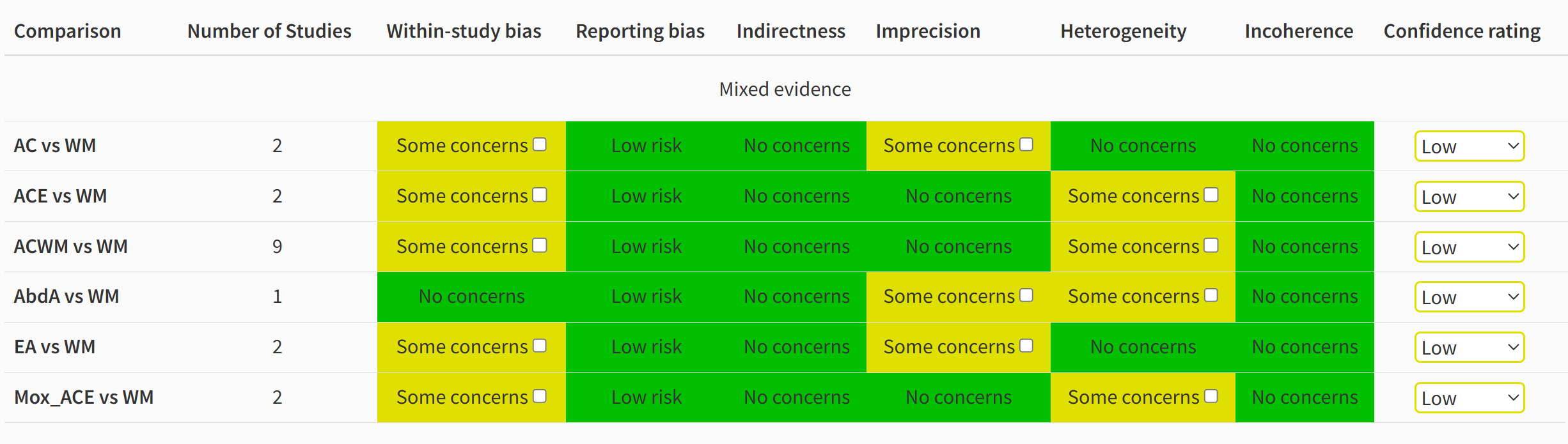


# S7. Network meta-analysis of T outcomes

**S7.1. Comparisons of consistency model and inconsistency model**

|  | **τ^2^** | **I^2^** | **DIC** | **‾D_res_^*^** |
| --- | --- | --- | --- | --- |
| Consistency model | 0.09 | 3% | 107.45 | 56.85 |
| Inconsistency model | 0.09 | 3% | 108.17 | 56.60 |

*Compared with 56 data points.

**S7.2. Heterogeneity**

| **Median** $\boldsymbol{\tau}$ **(95%CI)** | **Median τ^2^ (95%CI)** | **MCID** |
| --- | --- | --- |
| 0.29(0.20,0.44) | 0.09(0.04,0.20) | 0.63 |

MICD: Pooling all baseline T standard deviations included in the studies yielded an overall pooled standard deviation of 1.25nmol/L. Based on the distribution-based approach, the MCID threshold was defined as 0.5 × SD = 0.63 nmol/L.

**S7.3. Convergence assessment using the potential scale reduction factors**


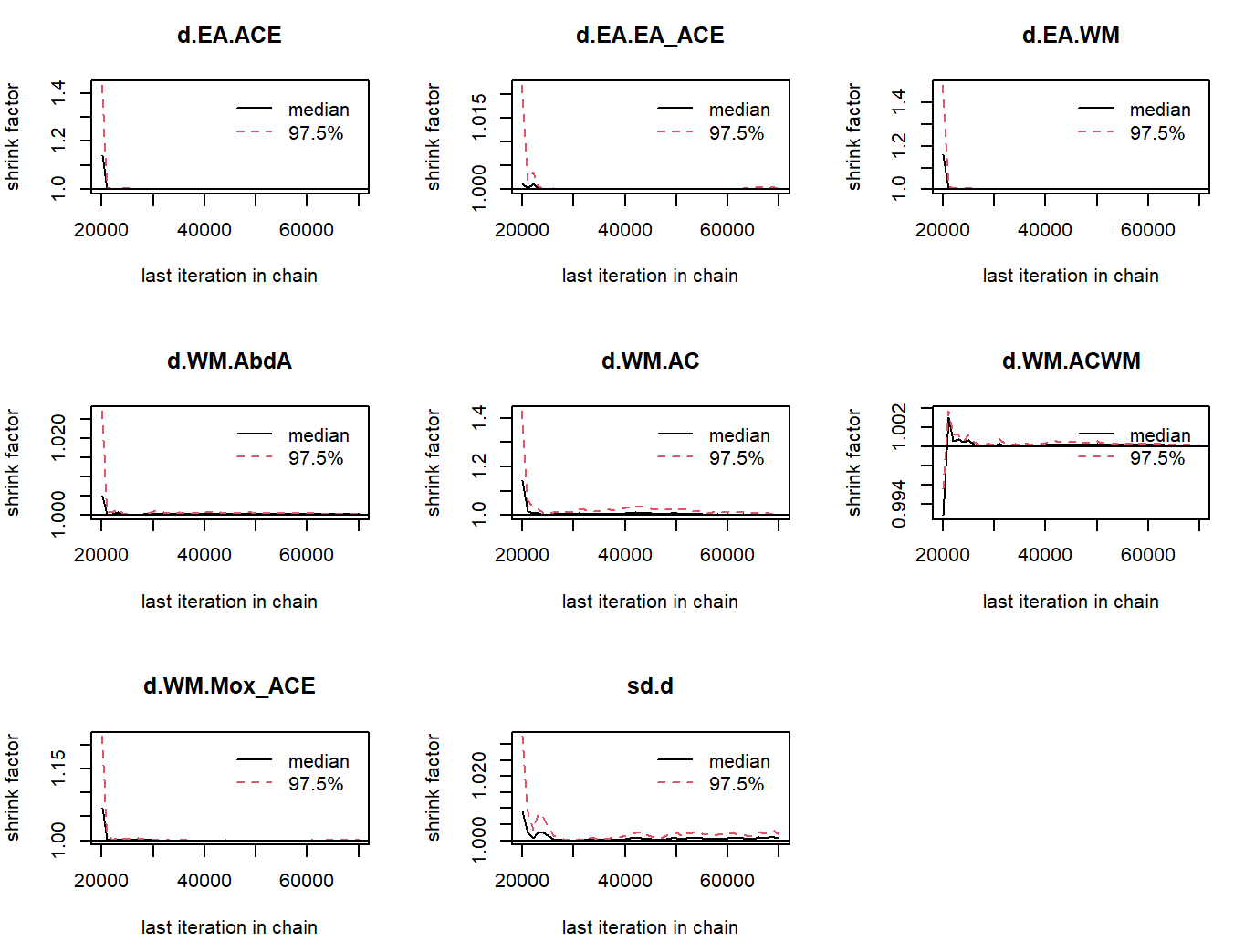


S7.4. Node-splitting results using Bayesian approach


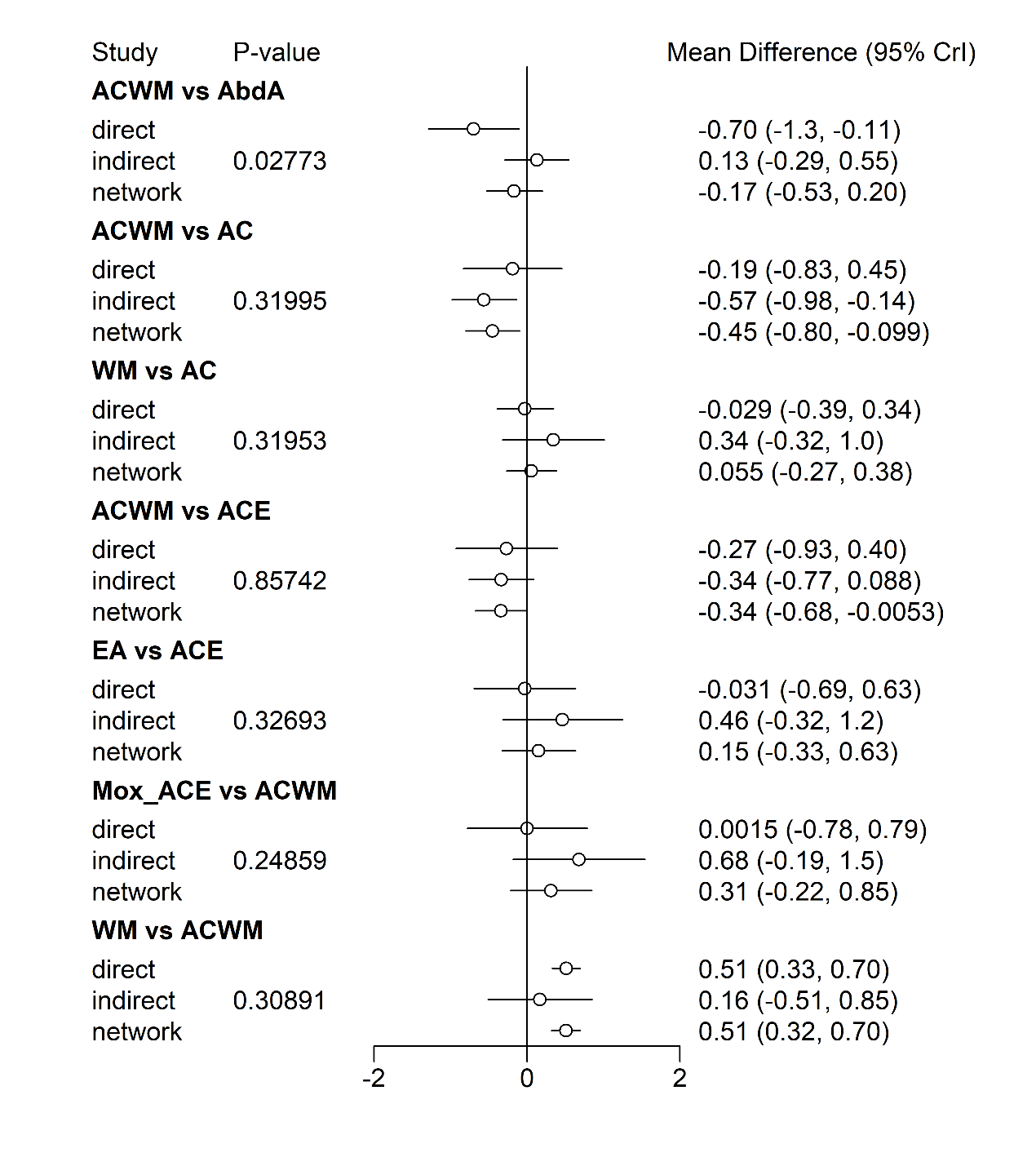


**ACWM,** Acupuncture Combined with Medication; **AbdA,** Abdominal Acupuncture; **Mox_ACE,**, Moxibustion plus Acupoint Catgut Embedding; **ACE,** Acupoint Catgut Embedding; **AC,** Acupuncture; **EA,** Electroacupuncture; **WM,** Western Medicine.

**S7.5. Sensitivity analysis treating each acupuncture-medication combination (ACWM) as an independent node**

**AC_M,** Acupuncture plus Metformin; **AC_CC,** Acupuncture plus Clomiphene Citrate; **AbdA_M,,** Abdominal Acupuncture plus Metformin; **Mox_M,** Moxibustion plus Metformin; **ACE_COC,** Acupoint Catgut Embedding plus Combined Oral Contraceptive**; ACE_M,** Acupoint Catgut Embedding plus Metformin; **AbdA,** Abdominal Acupuncture; **Orlistat_ACE,** Orlistat plus Acupoint Catgut Embedding**;** **Mox_ACE_COC_M,** Moxibustion plus Acupoint Catgut Embedding plus Combined Oral Contraceptive plus Metformin; **AC_acarbose,** Acupuncture plus acarbose**; ACE,** Acupoint Catgut Embedding; **Mox_ACE,**, Moxibustion plus Acupoint Catgut Embedding; **EA,**Electroacupuncture; **WM,** Western Medicine; **AC,** Manual Acupuncture; **EA_ACE,** Electroacupuncture plus Acupoint Catgut Embedding; **EA_Mox_M,** Electroacupuncture plus Moxibustion plus Metformin.

**S7.6. funnel plot**


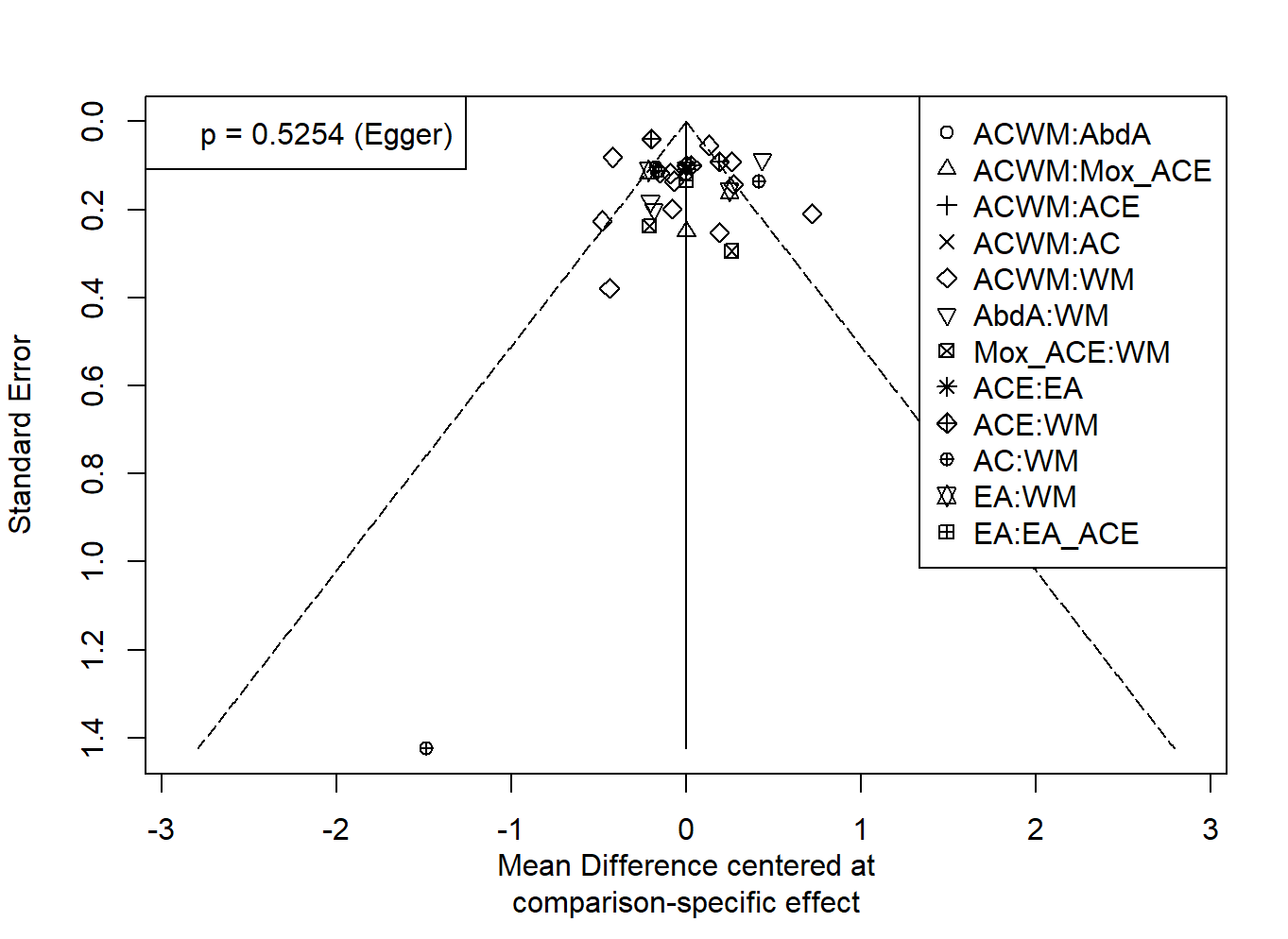


**S7.7. Results for meta-regression**

| Covariates | B (95%CI) | τ^2^ | I^2^ | DIC | ‾D_res_^*^ |
| --- | --- | --- | --- | --- | --- |
| Publication year | 0.20 (-0.10, 0.51) | 0.08 | 4% | 107.80 | 57.17 |
| Age (years) | -0.02 ( -0.31, 0.28) | 0.10 | 3% | 107.75 | 56.76 |
| Sample size | -0.08 ( -0.39, 0.22) | 0.10 | 3% | 107.51 | 56.61 |
| Acupuncture frequency (frequency/week) | -0.01(-0.34, 0.35) | 0.10 | 3% | 107.82 | 56.79 |
| Period of treatment (month) | -0.31 (-0.68, 0.07) | 0.09 | 2% | 106.61 | 56.06 |

*Compared with 56 data points.

**S7.8. Confidence assessment**


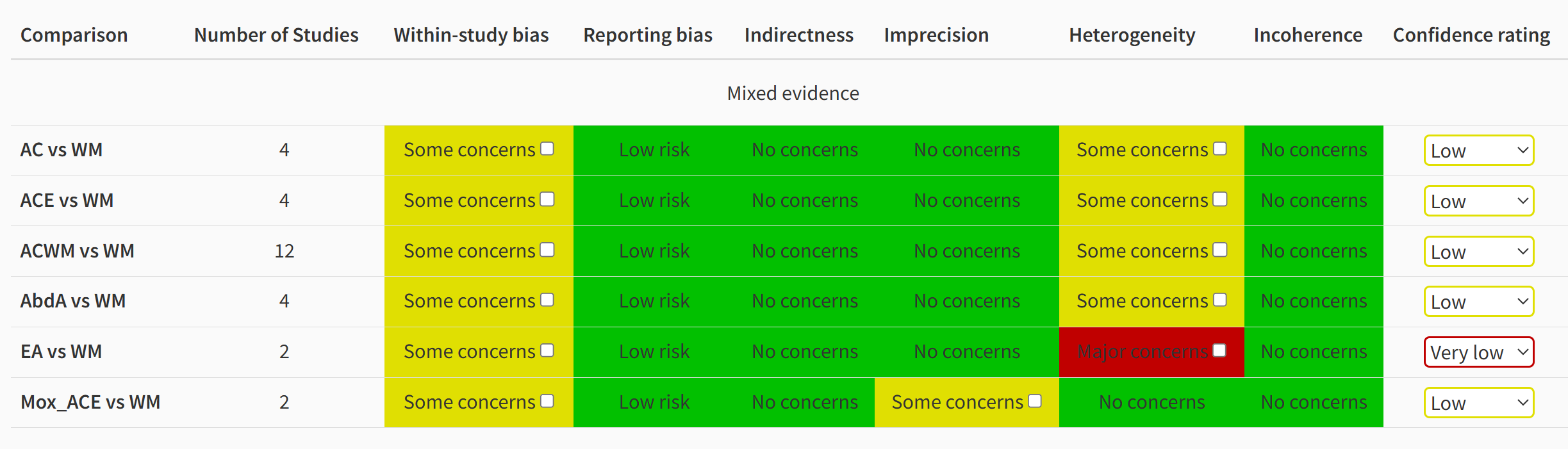


# S8. Network meta-analysis of LH/FSH ratio outcomes

**S8.1. Comparisons of consistency model and inconsistency model**

|  | **τ^2^** | **I^2^** | **DIC** | **‾D_res_^*^** |
| --- | --- | --- | --- | --- |
| Consistency model | 0.09 | 0% | 81.66 | 42.20 |
| Inconsistency model | 0.10 | 0% | 83.79 | 42.93 |

*Compared with 44 data points.

**S8.2. Heterogeneity**

| **Median** $\boldsymbol{\tau}$ **(95%CI)** | **Median τ^2^ (95%CI)** | **MCID** |
| --- | --- | --- |
| 0.29(0.19,0.46) | 0.08(0.04,0.20) | 0.41 |

MICD: Pooling all baseline LH/FSH ratio standard deviations included in the studies yielded an overall pooled standard deviation of 0.82. Based on the distribution-based approach, the MCID threshold was defined as 0.5 × SD = 0.41.

**S8.3. Convergence assessment using the potential scale reduction factors**


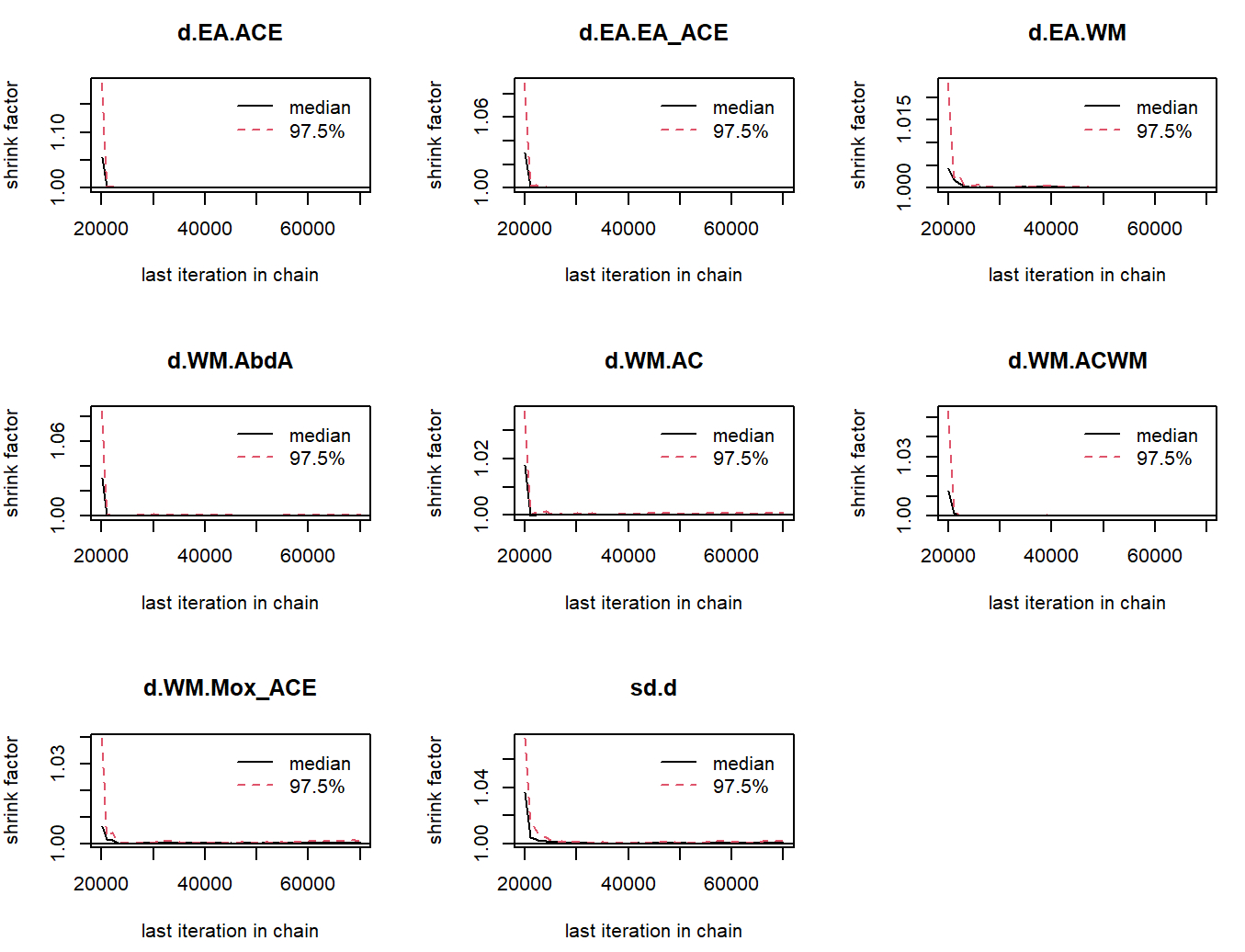


S8.4. Node-splitting results using Bayesian approach


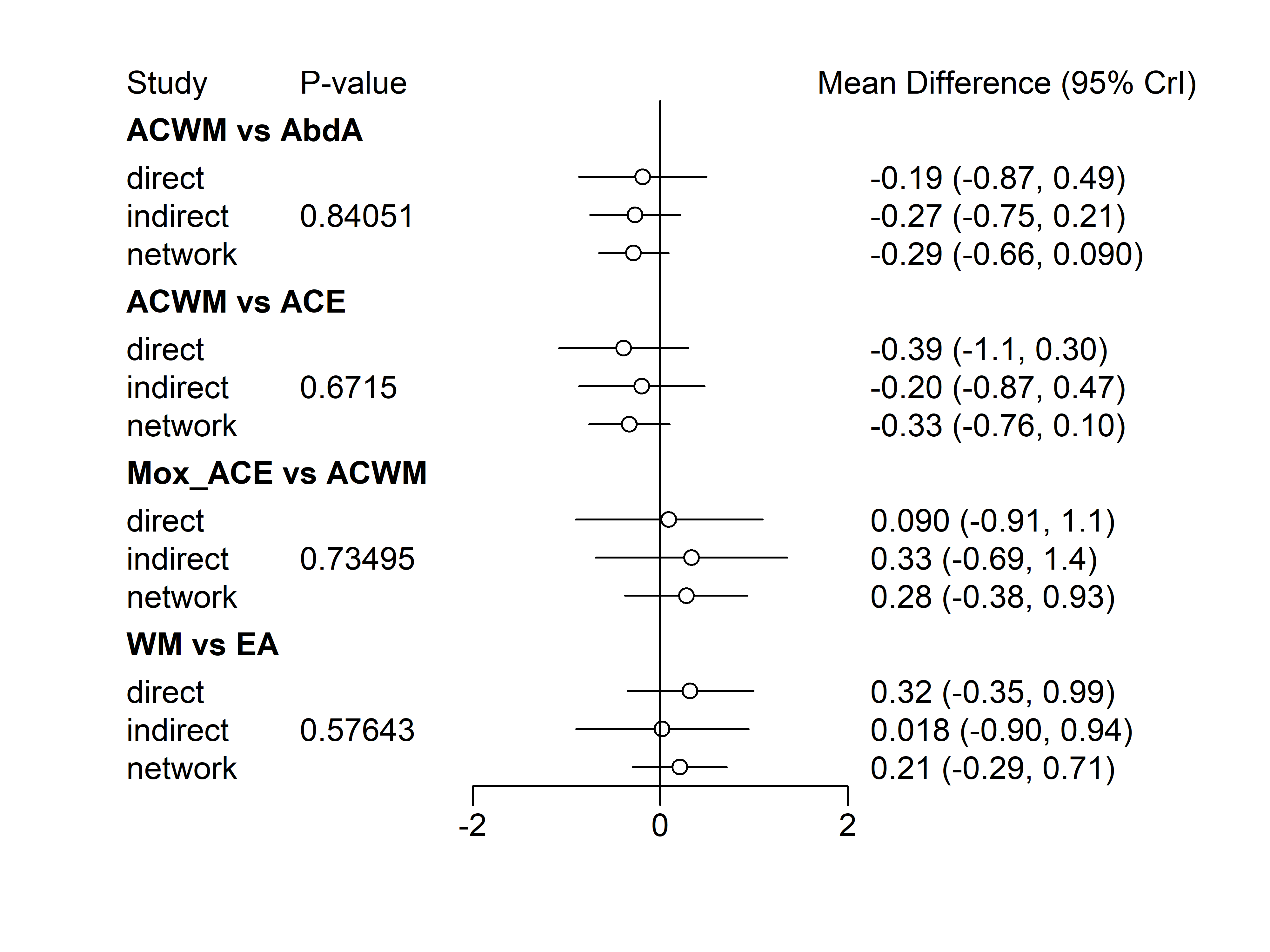


**ACWM,** Acupuncture Combined with Medication; **Mox_ACE,**, Moxibustion plus Acupoint Catgut Embedding; **AbdA,** Abdominal Acupuncture; **ACE,** Acupoint Catgut Embedding; **EA,** Electroacupuncture; **WM,** Western Medicine.

**S8.5.** **League table**

Treatments are ranked according to their surface under the curve cumulative ranking (SUCRA). Comparisons between treatments should be read from left to right and the estimate is in the cell in common between the column-defining treatment and the rowdefining treatment. For all figures, effect sizes were presented as MD with 95% CIs.

| **ACWM** |  |  |  |  |  |  |  |
| --- | --- | --- | --- | --- | --- | --- | --- |
| -0.27 (-0.73, 0.18) | **AC** |  |  |  |  |  |  |
| -0.28 (-0.81, 0.25) | -0.01 (-0.64, 0.64) | **EA** |  |  |  |  |  |
| -0.28 (-0.93, 0.38) | -0.01 (-0.76, 0.75) | 0 (-0.81, 0.81) | **Mox_ACE** |  |  |  |  |
| -0.29 (-0.66, 0.09) | -0.02 (-0.53, 0.51) | -0.01 (-0.6, 0.59) | -0.01 (-0.72, 0.7) | **AbdA** |  |  |  |
| -0.33 (-0.77, 0.1) | -0.07 (-0.62, 0.52) | -0.05 (-0.48, 0.37) | -0.05 (-0.81, 0.7) | -0.04 (-0.57, 0.47) | **ACE** |  |  |
| -0.37 (-1.19, 0.46) | -0.1 (-1, 0.82) | -0.09 (-0.72, 0.54) | -0.09 (-1.11, 0.94) | -0.08 (-0.96, 0.78) | -0.03 (-0.8, 0.72) | **EA_ACE** |  |
| **-0.49 (-0.7, -0.27)** | -0.22 (-0.61, 0.19) | -0.21 (-0.71, 0.29) | -0.21 (-0.85, 0.43) | -0.2 (-0.53, 0.13) | -0.15 (-0.56, 0.25) | -0.12 (-0.93, 0.69) | **WM** |

**ACWM,** Acupuncture Combined with Medication; **AC,** Manual Acupuncture; **EA,** Electroacupuncture; **Mox_ACE,**, Moxibustion plus Acupoint Catgut Embedding; **AbdA,** Abdominal Acupuncture; **ACE,** Acupoint Catgut Embedding; **EA_ACE,** Electroacupuncture plus Acupoint Catgut Embedding; **WM,** Western Medicine.

**S8.6. Sensitivity analysis treating each acupuncture-medication combination (ACWM) as an independent node**

**AC_Letrozole,** Acupuncture plus Letrozole**; AC_CC,** Acupuncture plus Clomiphene Citrate; **AC_M,** Acupuncture plus Metformin; **ACE_COC_M,** Acupoint Catgut Embedding plus Combined Oral Contraceptive plus Metformin**;** **ACE_M,** Acupoint Catgut Embedding plus Metformin; **AbdA_M,** Abdominal Acupuncture plus Metformin; **AC,** Manual Acupuncture; **Mox_ACE_COC_M,** Moxibustion plus Acupoint Catgut Embedding plus Combined Oral Contraceptive plus Metformin; **EA,** Electroacupuncture; **Mox_ACE,**, Moxibustion plus Acupoint Catgut Embedding; **AbdA,** Abdominal Acupuncture; **ACE,** Acupoint Catgut Embedding; **EA_ACE,** Electroacupuncture plus Acupoint Catgut Embedding; **WM,** Western Medicine.

**S8.7. funnel plot**


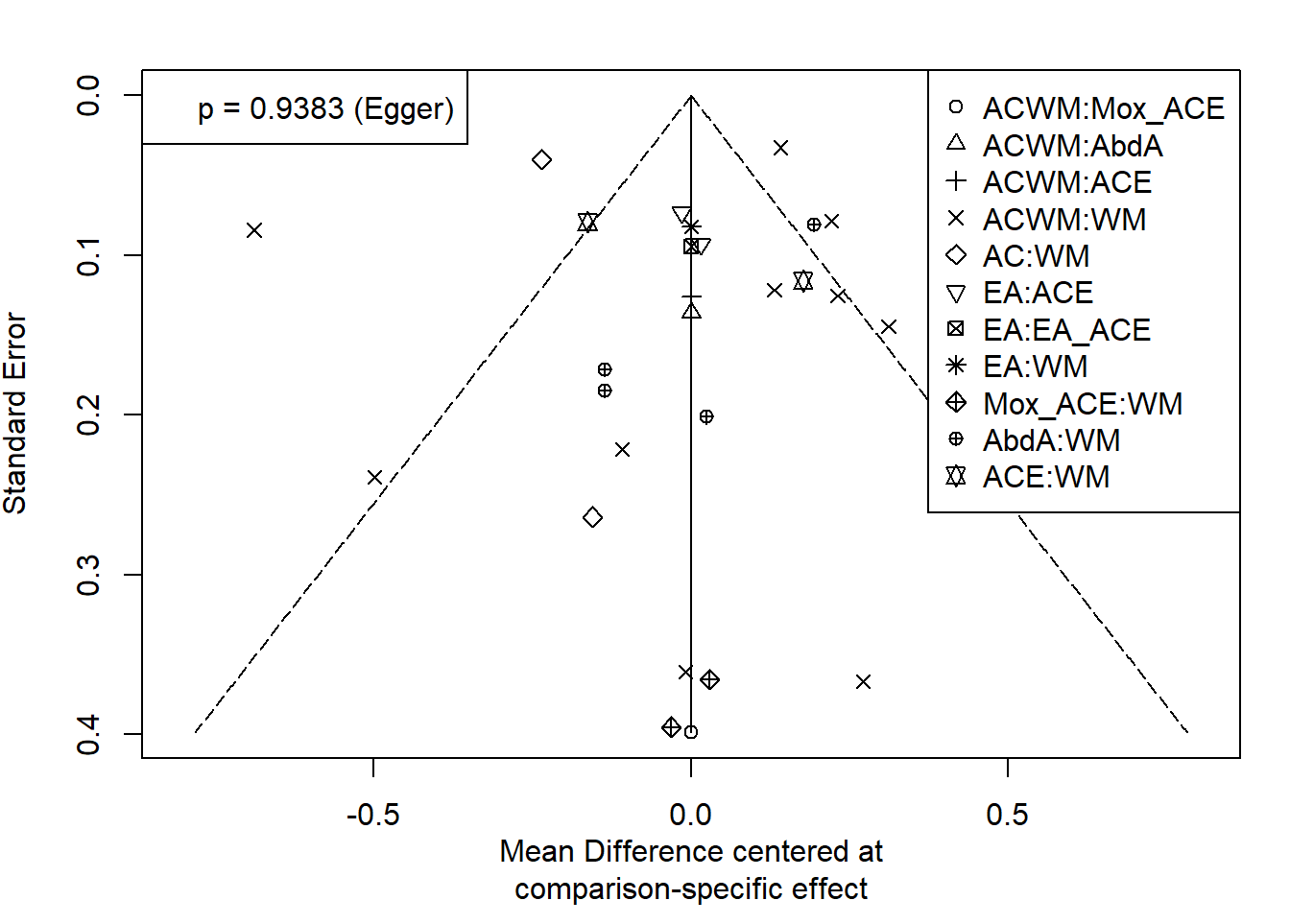


**S8.8. Results for meta-regression**

| **Covariates** | **B (95%CI)** | **τ^2^** | **I^2^** | **DIC** | **‾D_res_^*^** |
| --- | --- | --- | --- | --- | --- |
| Publication year | 0.02 (-0.39, 0.45) | 0.10 | 0% | 82.21 | 42.30 |
| Age (years) | -0.23 (-0.53, 0.10) | 0.07 | 1% | 82.73 | 43.42 |
| Sample size | -0.18 ( -0.57, 0.14) | 0.09 | 0% | 81.94 | 42.22 |
| Acupuncture frequency (frequency/week) | -0.08 (-0.45, 0.28) | 0.09 | 0% | 82.05 | 42.17 |
| Period of treatment (month) | -0.16(-0.58, 0.23) | 0.09 | 0% | 81.78 | 42.05 |

*Compared with 44 data points.

**S8.9. Confidence assessment**


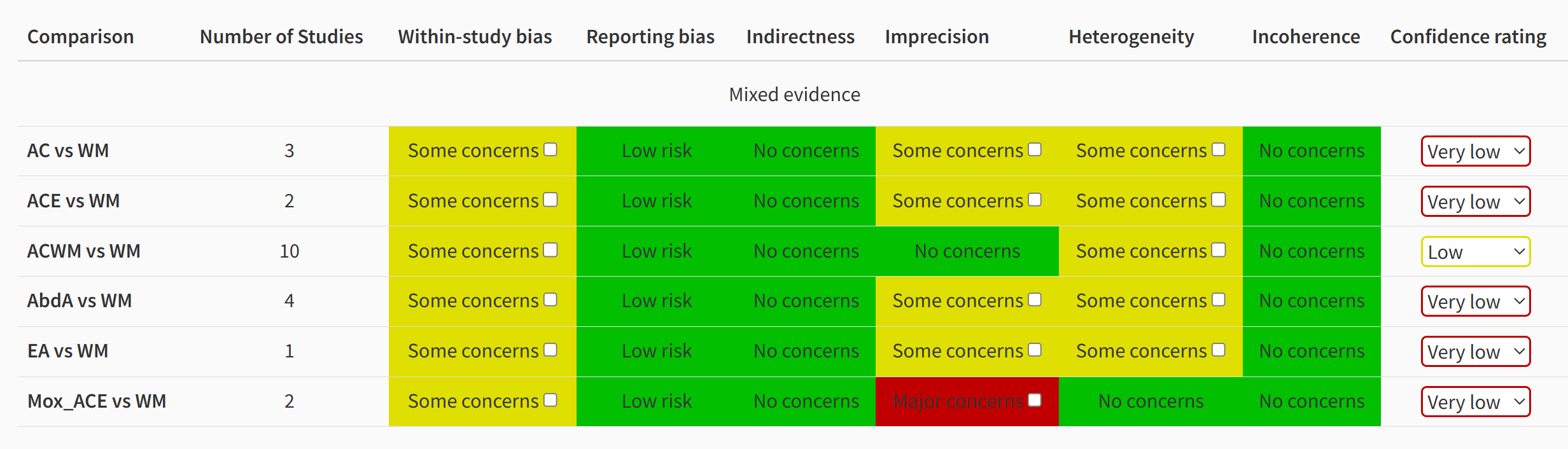


# S9. Network meta-analysis of TG outcomes

**S9.1. Comparisons of consistency model and inconsistency model**

|  | **τ^2^** | **I^2^** | **DIC** | **‾D_res_^*^** |
| --- | --- | --- | --- | --- |
| Consistency model | 0.04 | 0% | 46.32 | 24.56 |
| Inconsistency model | 0.05 | 0% | 49.50 | 26.10 |

*Compared with 26 data points.

**S9.2. Heterogeneity**

| **Median** $\boldsymbol{\tau}$ **(95%CI)** | **Median τ^2^ (95%CI)** | **MCID** |
| --- | --- | --- |
| 0.18(0.07,0.41) | 0.03 (0.01,0.17) | 0.40 |

MICD: Pooling all baseline HOMA-IR standard deviations included in the studies yielded an overall pooled standard deviation of 0.79. Based on the distribution-based approach, the MCID threshold was defined as 0.5 × SD = 0.40.

**S9.3. Convergence assessment using the potential scale reduction factors**


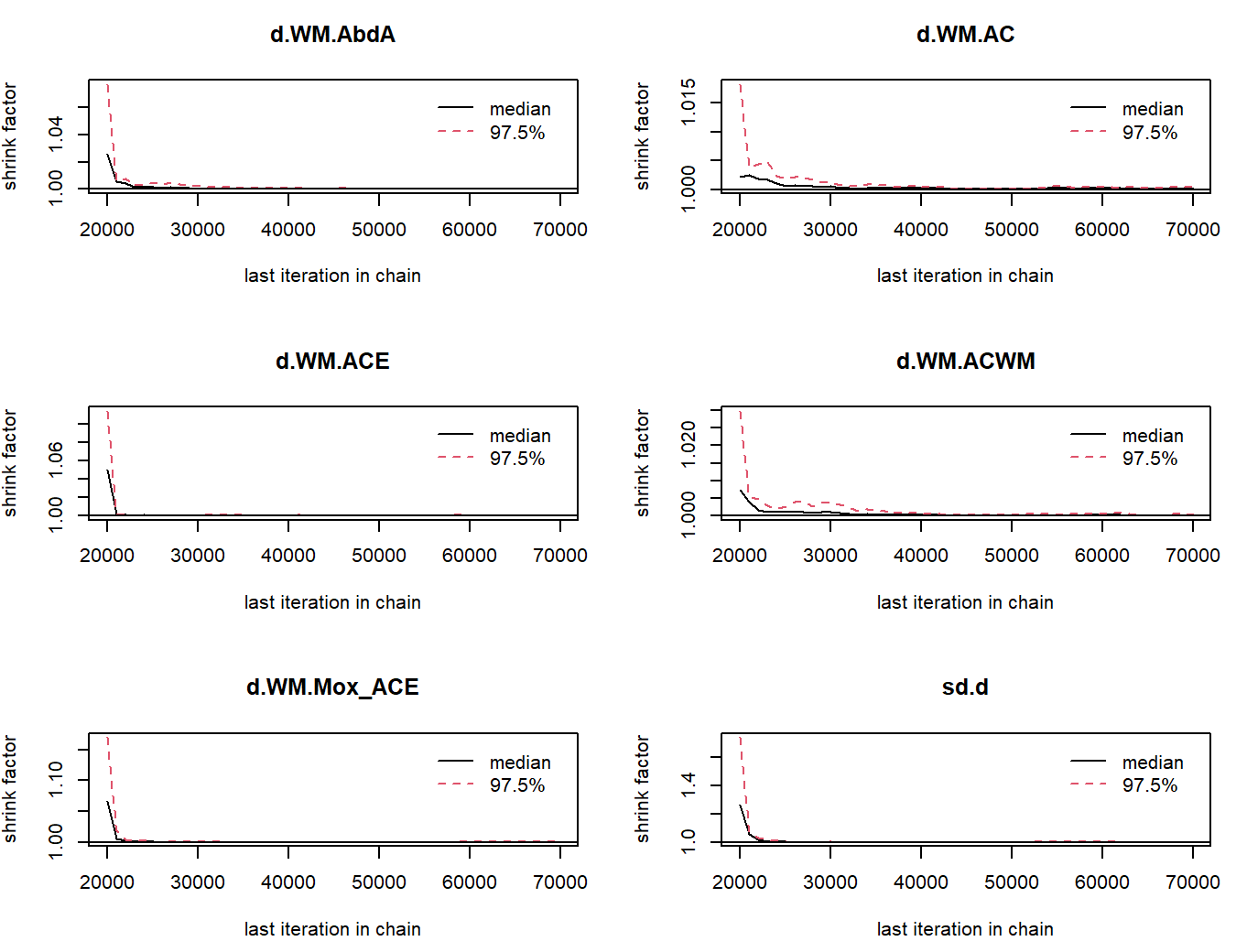


S9.4. Node-splitting results using Bayesian approach


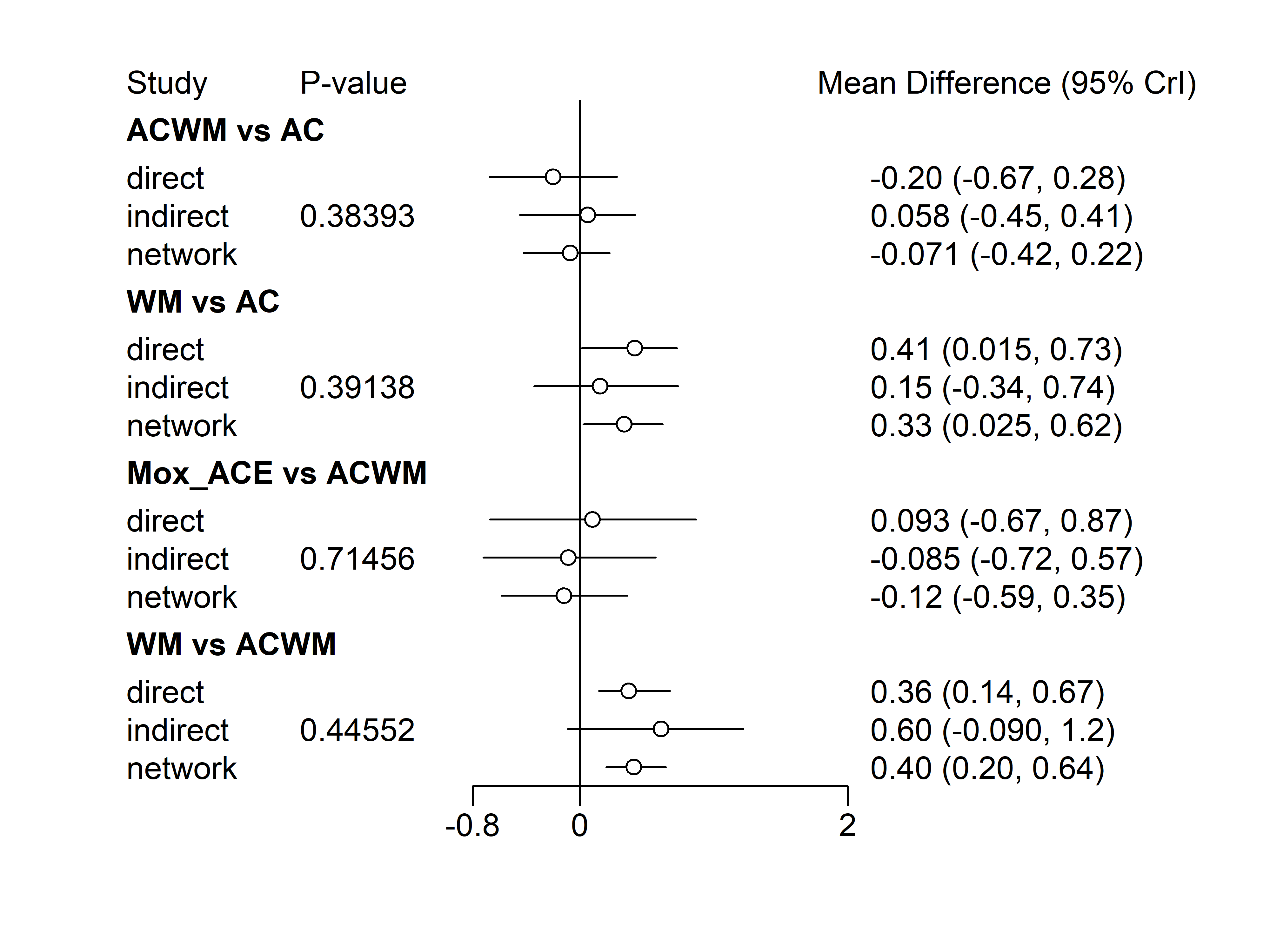


**Mox_ACE**, Moxibustion plus Acupoint Catgut Embedding; **ACWM,** Acupuncture Combined with Medication; **AC,** Acupuncture; **WM,** Western Medicine.

**S9.5.** **League table**

Treatments are ranked according to their surface under the curve cumulative ranking (SUCRA). Comparisons between treatments should be read from left to right and the estimate is in the cell in common between the column-defining treatment and the rowdefining treatment. For all figures, effect sizes were presented as MD with 95% CIs.

| **Mox_ACE** |  |  |  |  |  |
| --- | --- | --- | --- | --- | --- |
| -0.12 (-0.59, 0.36) | **ACWM** |  |  |  |  |
| -0.2 (-0.73, 0.31) | -0.07 (-0.42, 0.22) | **AC** |  |  |  |
| -0.32 (-0.91, 0.25) | -0.2 (-0.65, 0.22) | -0.13 (-0.6, 0.36) | **AbdA** |  |  |
| -0.33 (-0.93, 0.28) | -0.2 (-0.64, 0.21) | -0.13 (-0.61, 0.38) | 0 (-0.56, 0.57) | **ACE** |  |
| **-0.52 (-0.97, -0.09)** | **-0.4 (-0.64, -0.19)** | **-0.33 (-0.61, -0.03)** | -0.2 (-0.57, 0.18) | -0.19 (-0.63, 0.22) | **WM** |

**Mox_ACE**, Moxibustion plus Acupoint Catgut Embedding; **ACWM,** Acupuncture Combined with Medication; **AC,** Manual Acupuncture; **AbdA,** Abdominal Acupuncture; **ACE,** Acupoint Catgut Embedding; **WM,** Western Medicine.

**S9.6. Sensitivity analysis treating each acupuncture-medication combination (ACWM) as an independent node**

**Mox_ACE_COC_M,** Moxibustion plus Acupoint Catgut Embedding plus Combined Oral Contraceptive plus Metformin; **Mox_ACE**, Moxibustion plus Acupoint Catgut Embedding; **AC_acarbose,** Acupuncture plus acarbose; **EA_Mox_M,** Electroacupuncture plus Moxibustion plus Metformin; **AC,** Manual Acupuncture; **AAp_M,** Auricular Acupressure plus Metformin; **AC_CC,** Acupuncture plus Clomiphene Citrate; **ACE_M,** Acupoint Catgut Embedding plus Metformin; **AbdA,** Abdominal Acupuncture; **ACE,** Acupoint Catgut Embedding; **WM,** Western Medicine.

**S9.7. funnel plot**


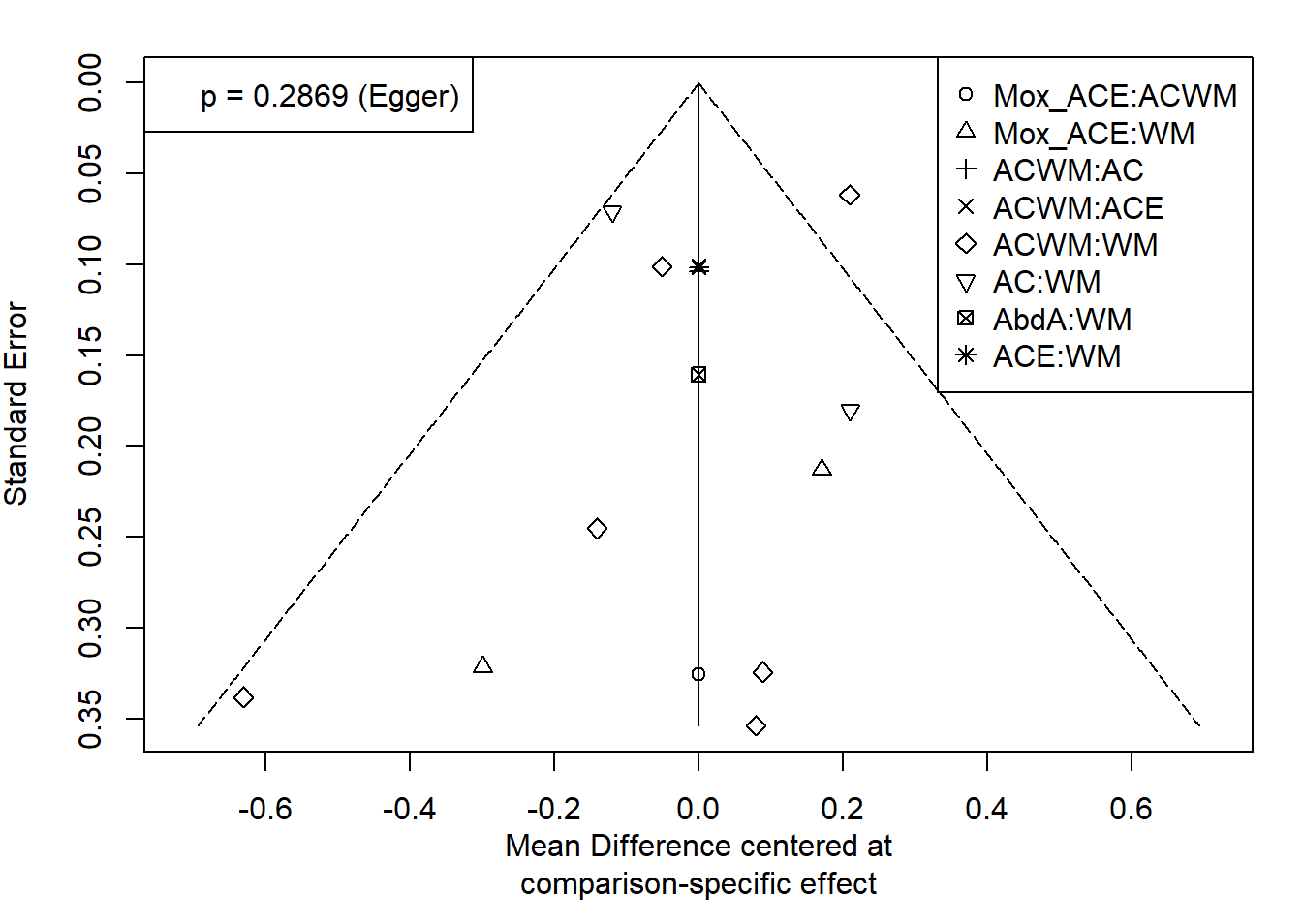


**S9.8. Results for meta-regression**

| **Covariates** | **B (95%CI)** | **τ^2^** | **I^2^** | **DIC** | **‾D_res_^*^** |
| --- | --- | --- | --- | --- | --- |
| Publication year | -0.14(-1.00, 0.72) | 0.04 | 0.8% | 47.42 | 25.20 |
| Age (years) | -0.36 (-0.69, 0.02) | 0.02 | 0% | 44.19 | 23.10 |
| Sample size | -0.20 (-0.49, 0.15) | 0.02 | 2% | 46.51 | 25.40 |
| Acupuncture frequency (frequency/week) | -0.00 (-0.44, 0.47) | 0.04 | 0.7% | 47.64 | 25.18 |
| Period of treatment (month) | -0.30 (-0.73, 0.17) | 0.02 | 0.3% | 46.34 | 25.07 |

*Compared with 26 data points.

**S9.9. Confidence assessment**


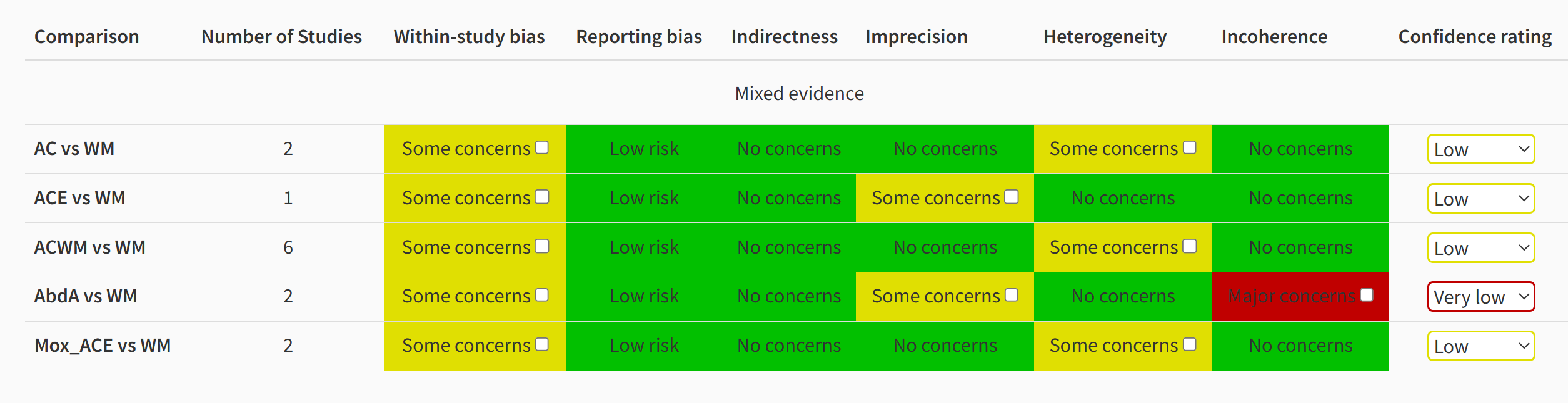


# S10 Side Effects and Adverse Reactions of Drugs

| **Study** | **Experimental group AEs** | **Control group AEs** | **Summary/Notes** |
| --- | --- | --- | --- |
| Wu Jia2020 | Not mentioned. | 2 cases of nausea and vomiting. | Probability of 6.90%, P > 0.05, The incidence of adverse reactions is not highly comparable. |
| Lai Maohua2010 | Not mentioned. | 21 cases experienced nausea, vomiting, diarrhea, and dizziness. | The incidence rate was 40.9%, with mild symptoms; after increasing the dose, symptoms became slightly more pronounced but remained tolerable. |
| Tianyu Wu2024 | 3 cases of localized bruising at acupoints 7 cases of localized pain after needling 1 case of localized hematoma at acupoint. | Not mentioned. | These adverse events are commonly observed phenomena in clinical acupuncture practice. |
| Li Yu′e2024 | Not mentioned. | 14 patients experienced nausea and vomiting after treatment, and 3 patients developed diarrhea. | Both groups of patients were able to adhere to treatment. |
| Yue Jin2021 | 1 case of subcutaneous hematoma. | 2 cases of elevated transaminases, 2 cases of irregular vaginal bleeding. | All groups experienced discomfort symptoms that resolved after rest. |
| Sheng Wenzhen2021 | 4 cases of skin discomfort with an overall incidence rate of 7.02%. | Four cases of gastrointestinal reactions, two cases of hypoglycemia, two cases of headache, and seven cases of breast tenderness, with an overall incidence rate of 25.86%. | The incidence of adverse reactions was significantly lower in the intervention group than in the control group, with a statistically significant difference (P < 0.05). |
| He Danjuan  2020 | Experimental Group 1: 2 cases of gastrointestinal reactions, 4 cases of skin discomfort, with an overall incidence rate of 10%. Experimental Group 2: 4 cases of gastrointestinal reactions, 3 cases of skin discomfort, 2 cases of hypoglycemia, 1 case of headache, 6 cases of breast tenderness, with an overall incidence rate of 27.4%. | Four cases of gastrointestinal reactions, two cases of hypoglycemia, one case of headache, and seven cases of breast tenderness, with an overall incidence rate of 22.6%. | The incidence of adverse reactions in the control group was statistically significantly different from that in the experimental group (P < 0.05). |
| Raden Muharam2022 | 2 cases of cephalic hematoma resolved within one week. | Not mentioned. | Compared to drug therapy alone, electroacupuncture has milder adverse reactions and is better tolerated by patients. |
| Jie Cao2023 | A small number of subjects experienced mild local pain or bruising at the injection site, which resolved without requiring special treatment. | Frequent gastrointestinal side effects include: Nausea,  Diarrhea, Abdominal distension, Loss of appetite. | Acupuncture appears to be a safe and well-tolerated alternative for PCOS patients, particularly those who are intolerant to metformin. |
| Yan-Hua Zheng 2013 | No uncomfortable symptoms occurred in the abdominal acupuncture group. | A total of 21 cases (approximately 49% of subjects in the group) experienced mild adverse reactions: 7 cases of nausea or vomiting, 10 cases of mild diarrhea, and 4 cases of mild dizziness or fatigue. | These symptoms all occurred during the initial treatment period and were classified as mild, transient gastrointestinal adverse reactions that did not require discontinuation of the medication. |
| Wu Dan2020 | 3 cases of redness and swelling; adverse reaction incidence rate: 5.36%. | 3 cases of gastrointestinal discomfort, 3 cases of dizziness and headache, 1 case each of muscle pain, hypotension, and rash; adverse reaction incidence rate: 16.07%. | The incidence of adverse reactions in the experimental group was significantly lower than that in the control group, with a statistically significant difference (P < 0.05). |
